# A scalable variational inference approach for increased mixed-model association power

**DOI:** 10.1038/s41588-024-02044-7

**Published:** 2025-01-09

**Authors:** Hrushikesh Loya, Georgios Kalantzis, Fergus Cooper, Pier Francesco Palamara

**Affiliations:** 1 Department of Statistics, University of Oxford, Oxford, UK; 2 Centre for Human Genetics, University of Oxford, Oxford, UK; 3 Wellcome Sanger Institute, Wellcome Genome Campus, Hinxton, UK; 4 Doctoral Training Centre, University of Oxford, Oxford, UK

**Keywords:** Genome-wide association studies, Software

## Abstract

The rapid growth of modern biobanks is creating new opportunities for large-scale genome-wide association studies (GWASs) and the analysis of complex traits. However, performing GWASs on millions of samples often leads to trade-offs between computational efficiency and statistical power, reducing the benefits of large-scale data collection efforts. We developed Quickdraws, a method that increases association power in quantitative and binary traits without sacrificing computational efficiency, leveraging a spike-and-slab prior on variant effects, stochastic variational inference and graphics processing unit acceleration. We applied Quickdraws to 79 quantitative and 50 binary traits in 405,088 UK Biobank samples, identifying 4.97% and 3.25% more associations than REGENIE and 22.71% and 7.07% more than FastGWA. Quickdraws had costs comparable to REGENIE, FastGWA and SAIGE on the UK Biobank Research Analysis Platform service, while being substantially faster than BOLT-LMM. These results highlight the promise of leveraging machine learning techniques for scalable GWASs without sacrificing power or robustness.

## Abstract (web summary)

Quickdraws is a mixed-model association tool with a noninfinitesimal prior for analyzing binary and quantitative traits, using a scalable variational inference that allows analysis of biobank-scale cohorts.

## Main

Genome-wide association studies (GWASs) are key in analyzing complex traits and diseases^1^, facilitating applications such as polygenic risk prediction^2–4^. These studies have been driven by the rapid growth of modern biobanks^5–10^, which contain vast amounts of genomic, phenotypic and environmental data across diverse groups^11^. The scale of these datasets, however, creates computational challenges, which have fostered the development of scalable linear-mixed-model (LMM) algorithms^12–23^. Among these, BOLT-LMM^21,22^ uses a Bayesian mixture prior to provide state-of-the-art association power but is computationally demanding, especially for multiple traits, and has limited applicability to binary traits, because it may result in inflated association statistics when case–control ratios are imbalanced. More recent methods, including SAIGE^24^, FastGWA^23,25^ and REGENIE^26^ are highly scalable and allow analysis of both quantitative and binary traits. These algorithms have enabled GWASs on millions of variants and individuals^27^, but rely on modeling approximations, such as block-wise ridge regression, which assumes that genetic effects are normally distributed, or sparse genetic matrices, which account only for close genetic relationships. As a result, current GWAS methods are either highly scalable and resource efficient or highly powered, but not both.

We describe a method, called Quickdraws, that uses machine learning to simultaneously achieve state-of-the-art association power and computational efficiency for both quantitative and binary traits. Quickdraws uses a Bayesian regression model with a spike-and-slab prior on variant effects, efficiently trained using stochastic variational inference, transfer learning and graphics processing unit (GPU) matrix operations. In simulations, Quickdraws matches the power of BOLT-LMM for quantitative traits while requiring substantially less computation. For binary traits, it achieves higher power than SAIGE, REGENIE and FastGWA-GLMM, equivalent to analyzing up to 13.4% more samples with comparable speed. We applied Quickdraws and other methods to 13.3 million variants for 79 quantitative and 50 disease traits in ~405,000 UK Biobank individuals^5^. Quickdraws identifies 4.97% and 22.71% more associations than REGENIE and FastGWA for quantitative traits and 3.25% and 7.07% more for disease traits. These results lead to similar gains in replicated signals in Biobank Japan^6^ and FinnGen^10^. Despite these power gains, we observed Quickdraws’ costs on the UK Biobank Research Analysis Platform (RAP) platform to be comparable to other highly scalable methods.

## Results

### Overview of the Quickdraws algorithm

Quickdraws is a mixed-model association algorithm that models phenotypes as a combination of fixed effects, random genetic effects and random environmental effects (Methods and equations (5) and (9)). Like other modern GWAS algorithms^21–26^, Quickdraws detects associations using two steps, which we refer to as model fitting and testing.

In the model fitting step, Quickdraws first estimates genetic and environmental variance components. For quantitative traits, this is done using randomized Haseman–Elston regression (RHE), a fast method-of-moments approach^28–30^. For binary traits, we instead performed a grid search over heritability values. Quickdraws then used a leave-one-chromosome-out (LOCO) scheme^17,19,21,31^ to predict phenotypes from genotype data. This step, which plays a key role in increasing association power^31^, is performed via Bayesian regression, using variance component estimates to set the prior. Similar to BOLT-LMM, which uses a mixture of Gaussian prior on variant effects^21,22^ to model nonpolygenic trait architectures, Quickdraws increases association power by adopting a spike-and-slab prior. Other scalable methods, such as REGENIE and FastGWA, rely on more computationally tractable Gaussian priors that assume fully polygenic traits.

Quickdraws incorporates several technical advancements to achieve scalability while maintaining high power. First, Quickdraws uses stochastic variational inference^32,33^, optimized with first-order optimizers^34,35^, to enable linear scaling with sample size, whereas BOLT-LMM requires ${\mathcal{O}}({N}^{1.5})$ computation for *N* individuals. Second, gradient-based stochastic variational inference allows Quickdraws to leverage modern computing architectures, such as GPUs. By offloading matrix multiplications and gradient evaluations to GPUs, Quickdraws achieves substantial speedups over central processing unit (CPU)-based computation. Finally, Quickdraws accelerates convergence by initializing LOCO runs with effect estimates from the whole-genome model, reducing computation without compromising accuracy^36^. Further details on Bayesian regression and its computational optimizations are provided in Methods and Supplementary Note.

During the testing step, Quickdraws uses these estimated genetic effects to compute a score-based test statistic for a linear or logistic mixed model, which is approximated up to a constant of proportionality^21,37,38^. This constant is later estimated by matching an estimate of the scaled effective sample size^39^ from linear or logistic regression on an unrelated and homogeneous subset of the data. We additionally correct for potential instability in the score-based test statistic that may be the result of case–control imbalance in binary traits^24^, using approximate Firth’s logistic regression^26^. We further optimize the calculation of test statistics using Numba^40^ and parallelize matrix operations across multiple cores. Additional details on the calculation of test statistics, their calibration and computational optimizations can be found in Methods and Supplementary Note.

### Performance in simulated data

We assessed the statistical power and robustness of Quickdraws by performing extensive simulations using 50,000 samples from the UK Biobank dataset^5^ genotyped at 512,828 variants, of which 54,568 were rare, having a minor allele frequency (MAF) between 10^−4^ and 10^−2^. We simulated 50 realistic heritable traits with a narrow-sense heritability ${h}_{g}^{2}=0.4$, polygenicity (defined as the proportion of considered variants with non-zero effects) between 0.25% and 10% and a MAF-dependent architecture with parameter *α* = −0.3, where *α* determines the relationship between MAF and effect sizes^41–43^. To obtain sample compositions that are representative of varying levels of population structure and relatedness that may be encountered in genomic datasets, we built three groups, each of 50,000 samples, using ancestry and relatedness information defined in ref. ^5^. These included a set of unrelated, self-reported white British individuals (referred to as GB-unrel), a set of white British individuals enriched for the presence of relatives (3.4× more first- to third-degree relatives compared with the full white British subset of UK Biobank^5^; referred to as GB-rel) and a set containing 50% British and 50% non-British European individuals (referred to as EUR), with a similar level of relatedness compared with the full white British subset. We used these groups to simulate the presence of a shared environmental component of the phenotype among close relatives as well as ancestry-based population stratification (Methods). We used sample coordinates on the top ten principal components (PCs) computed using genotype data as covariates for Quickdraws and all other models. Simulated causal variants were sampled only using odd chromosomes, which we used to assess statistical power, whereas noncausal variants on even chromosomes were utilized to evaluate statistical robustness to population relatedness and stratification.

#### Statistical power

**Fig. 1:**
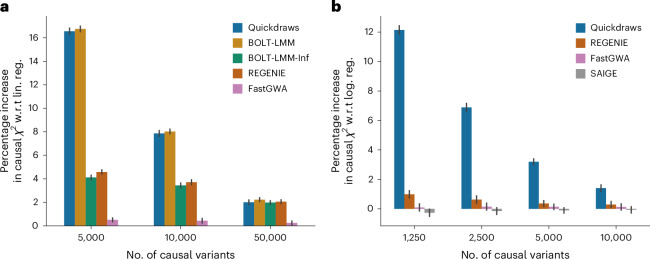
Statistical power in simulated quantitative and binary traits for unrelated British samples. **a**, Percentage increase in average *χ*^2^ test statistics at causal variants with respect to (w.r.t.) linear regression (lin. reg.) for quantitative traits, varying the number of simulated causal variants from 5,000 to 50,000 variants. **b**, Percentage increase in average *χ*^2^ test statistics at causal variants with respect to logistic regression (log. reg.) for binary traits, varying the number of simulated causal variants from 1,250 to 10,000 variants. Traits in **a** and **b** are simulated for 50,000 samples with *h*^2^ = 0.4; the prevalence is fixed to 30% for binary traits in **b**. Error bars are presented as mean value ± s.e. of the percentage improvement, measured using 50 independent traits. The causal *χ*^2^ is normalized by the mean *χ*^2^ at null variants for each trait.

We first tested the performance of Quickdraws in quantitative trait association and compared it with previous association testing methods including BOLT-LMM, REGENIE, FastGWA and an implementation of linear regression provided in PLINK^44,45^. For BOLT-LMM, we also considered a faster implementation that uses infinitesimal priors (BOLT-LMM-Inf), which is more scalable but does not lead to higher association power in less polygenic genetic architectures^21,22^. We measured statistical power by considering the average *χ*^2^ test statistics at simulated causal variants, focusing on the set of unrelated white British samples and varying the number of causal variants in the simulation from 5,000 to 50,000 (ref. ^42^). We normalized the average *χ*^2^ test statistics at causal variants by the average *χ*^2^ test statistics at null variants to remove any residual stratification. In these simulations, the modeling of trait polygenicity adopted by Quickdraws led to higher average *χ*^2^ statistics (Student’s paired *t*-test *P* < 10^−20^ for polygenicity up to 2%) compared with REGENIE, FastGWA, BOLT-LMM-Inf and linear regression (Fig. 1a and Supplementary Fig. 1). The difference in association power compared with other infinitesimal methods was larger for traits with lower polygenicity. For instance, when the number of causal variants was reduced to 5,000 (1% polygenicity), Quickdraws achieved ≥11.46% higher average *χ*^2^ compared with REGENIE and BOLT-LMM-Inf (Student’s paired *t*-test *P* < 10^−53^), resulting in a similarly large gain in effective sample size^39^.

Next, we applied Quickdraws to the analysis of binary traits, comparing its association power with previous binary trait association methods, including SAIGE, REGENIE and FastGWA-GLMM. We simulated phenotypes under a liability threshold model, where cases and controls are defined as individuals who are above or below a chosen liability threshold (Methods). We fixed the prevalence at 0.3 and varied the number of causal variants in the simulation from 1,250 to 10,000 (refs. ^42,46,47^). In these experiments, the use of a noninfinitesimal prior in Quickdraws led to higher statistical power compared with SAIGE, REGENIE and FastGWA-GLMM (Student’s paired *t*-test *P* < 10^−12^; Fig. 1b and Supplementary Fig. 2). The difference in association power compared with other methods was again larger for traits with lower polygenicity (Student’s paired *t*-test *P* < 10^−43^ for 1,250 and 2,500 causal variants). For instance, Quickdraws obtained 11.5% and 12.04% higher average *χ*^2^ compared with REGENIE and FastGWA-GLMM on the simulated causal variants for traits with a low polygenicity of 0.25%.

**Extended Data Fig. 1:**
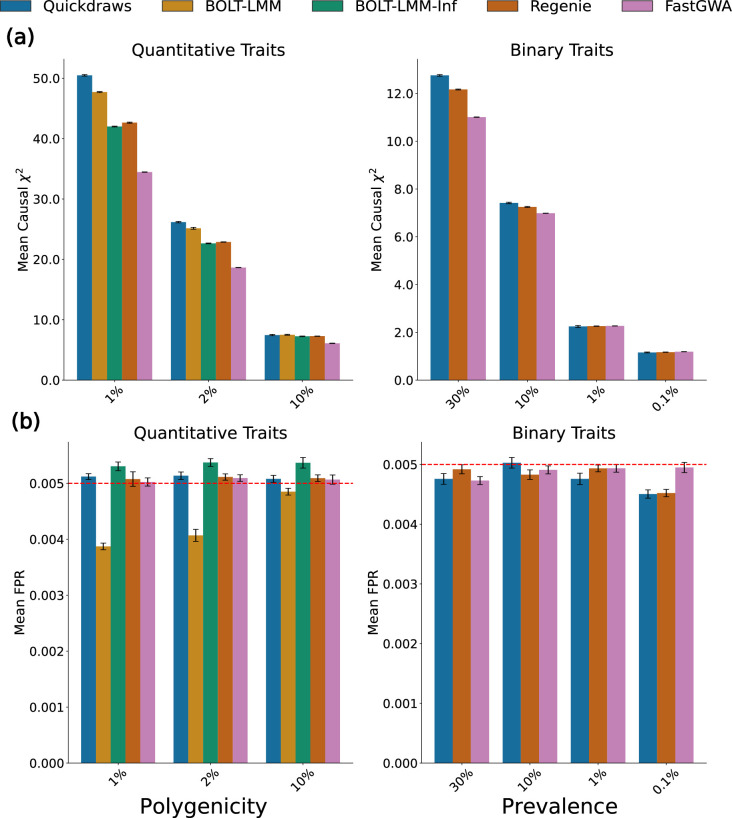
Power and FPR in N = 405k simulation. (**a**) The mean *χ*^2^ at causal variants across different methods and polygenicities for Quantitative traits, and prevalence for binary traits. The *χ*^2^ at causal variants is not normalized by the *χ*^2^ at null variants in this plot. (**b**) The mean false positive rates (FPR) at 0.005 calculated at null variants across different methods and polygenicities for Quantitative traits, and prevalence for binary traits. The simulation was performed using the N≈ 405,000 set of white British individuals (See Methods for more details on simulations). The error bars represent 95% confidence intervals. The red dashed line corresponds to false positive rate = 0.005.

**Extended Data Fig. 2:**
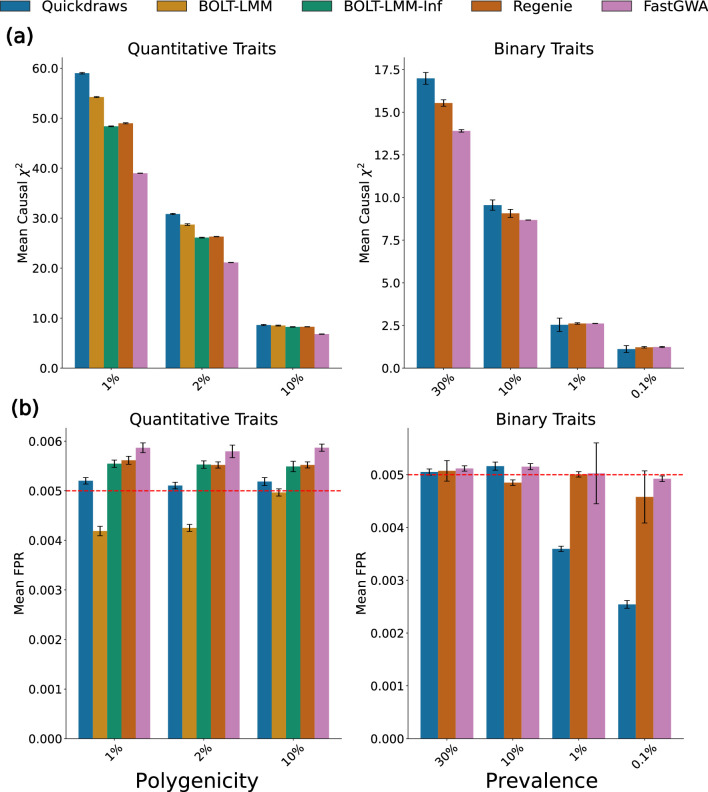
Power and FPR in N= 460k simulation. (**a**) The mean *χ*^2^ at causal variants across different methods and polygenicities for quantitative traits, and prevalence for binary traits. The *χ*^2^ at causal variants is not normalized by the *χ*^2^ at null variants in this plot. (**b**) The mean false positive rates (FPR) at 0.005 calculated at null variants across different methods and polygenicities for Quantitative traits, and prevalence for binary traits. The simulation was performed using the N≈ 460,000 set of UK Biobank self-identified Europeans [5]. The error bars represent 95% confidence intervals. The red dashed line corresponds to false positive rate = 0.005.

Finally, we tested Quickdraws in simulations where causal effects were not assumed to follow a spike-and-slab distribution, which models only a subset of variants as contributing to the phenotype. Instead, we sampled effects from a Gaussian, a mixture of Gaussians or a Laplace distribution. In the mixture of Gaussian setting, we again observed that Quickdraws and BOLT-LMM yielded higher power compared with other models. In simulations involving fully infinitesimal traits with Laplace and Gaussian effects, Quickdraws and BOLT-LMM produced results similar to other approaches that assume a fully infinitesimal trait architecture (Supplementary Fig. 3). We also evaluated power in simulations involving all white British individuals (*N* ≈ 405,000) and all self-identified European individuals from the UK Biobank (*N* ≈ 460,000). In these settings, we found that Quickdraws provided the highest association power, with higher power than BOLT-LMM when the polygenicity of the trait was low (Extended Data Figs. 1 and 2).

#### Calibration

We next verified the calibration of Quickdraws and the other methods by considering varying levels of polygenicity, relatedness, population structure, and the prevalence of binary traits. We measured false-positive rates (FPRs), calculated as the proportion of null variants (that is, variants on even chromosomes) below a chosen *P*-value threshold.

We first tested calibration in analyses involving quantitative traits, considering several simulation settings with population structure and relatedness and varying polygenicity of traits from 1% to 10%. In these experiments, linear regression was not robust to the presence of relatedness and population structure, as previously observed^21,23,31^, whereas all other methods were reasonably calibrated, with a few exceptions (Supplementary Fig. 4 and Supplementary Table 1). Similar to other mixed-model methods, Quickdraws remained calibrated, not showing significant inflation in any of the simulation conditions that we considered. REGENIE produced significantly inflated test statistics in datasets with high levels of relatedness (Student’s *t*-test *P* < 1.5 × 10^−4^; see GB-rel+ in Supplementary Fig. 4), which comprise 1,250 first-degree and 1,250 second-degree relative pairs (corresponding to ~7.3× and ~4.8× more relatives compared with the white British subset of UK Biobank samples). This may be because REGENIE does not explicitly account for relatedness. Finally, we observed higher variance for Quickdraws’ FPR estimates in simulations involving nonhomogeneous ancestry, due to higher noise in the estimated effective sample size, which relies on a smaller number of unrelated homogeneous individuals (Methods).

We then assessed statistical robustness in the analysis of binary traits, where we varied the prevalence of the trait from 0.3 to 0.001 under varying levels of population structure and relatedness (Methods, Supplementary Figs. 5 and 6 and Supplementary Table 1) while fixing the polygenicity of the traits to 2%. Binary trait association analysis often violates the normality assumptions made when defining the null distribution of association statistics in linear-mixed models, thereby resulting in a high FPR, particularly for lower frequency variants or traits with lower prevalence^24^. Consistent with this, BOLT-LMM and BOLT-LMM-Inf, which are not designed for the analysis of low-prevalence binary traits, were inflated across all simulated conditions (Student’s *t*-test *P* < 3 × 10^−5^) when the prevalence dropped to or <0.1. Quickdraws produced controlled FPRs for both common (MAF ≥ 1%) and rare (MAF < 1%) variants in all the simulation settings that we considered, which included population structure, relatedness and low-prevalence binary traits (Supplementary Figs. 5 and 6, respectively). REGENIE with Firth’s logistic regression fallback was inflated for common variants in traits with high prevalence (prevalence = 0.1 and 0.3) for several of the population structure and relatedness settings that we considered. When the prevalence of the trait was set to ≤0.01 (that is, 500 cases out of 50,000 samples), both Quickdraws and REGENIE yielded deflated test statistics (Student’s *t*-test *P* < 3 × 10^−5^); in scenarios with such a low prevalence, however, all methods that we considered lacked sufficient statistical power to detect association (*χ*^2^ at causal variants ≈ 1). SAIGE did not converge for up to 28% of the traits when the prevalence dropped to 0.001, leading to significant deflation (Student’s *t*-test *P* < 10^−15^).

Finally, we verified the calibration of FPRs in various other simulated conditions, including stronger population structure with up to 30% non-European ancestry (Methods and Supplementary Fig. 7), varying levels of relatedness (Supplementary Fig. 8) and genetic architectures involving diverse causal effect-size distributions (Supplementary Fig. 9). We also evaluated FPRs in simulations involving all white British individuals (*N* ≈ 405,000) and all self-identified European individuals from the UK Biobank (*N* ≈ 460,000), varying levels of polygenicity in quantitative traits (1–10%) and varying levels of prevalence (0.3–0.001) in binary traits. We found Quickdraws to yield controlled FPRs across varying significance thresholds, whereas FastGWA, REGENIE and BOLT-LMM-Inf led to significant inflation in some cases (Extended Data Figs. 1 and 2 and Supplementary Tables 2 and 3). This is probably the result of residual population stratification, which is not fully corrected using PCs in these simulated scenarios and, if present, may cause subtle inflation in real-data analyses^48–50^.

Overall, these simulations demonstrate that the use of a noninfinitesimal spike-and-slab prior results in higher statistical power to detect association in both quantitative and binary trait simulations. Quickdraws matched or outperformed the power of BOLT-LMM in the analysis of quantitative traits and obtained higher association power than existing GWAS algorithms in binary traits. Quickdraws also yielded controlled FPRs in all simulation settings that we considered, which included population structure, relatedness and low-prevalence binary traits.

### UK Biobank analysis

We applied Quickdraws to 79 quantitative traits (blood-related, anthropometric and other traits) and 50 self-reported diseases in ~405,000 white British individuals from the UK Biobank (Supplementary Tables 4 and 5), selected for their high phenotyping rate and heritability (Methods). We applied standard quality-control filters, retaining 458,420 markers for model fitting and testing ~13.3 million imputed variants (Methods). We compared the association statistics obtained from Quickdraws with those of SAIGE, FastGWA, REGENIE and BOLT-LMM.

**Fig. 2:**
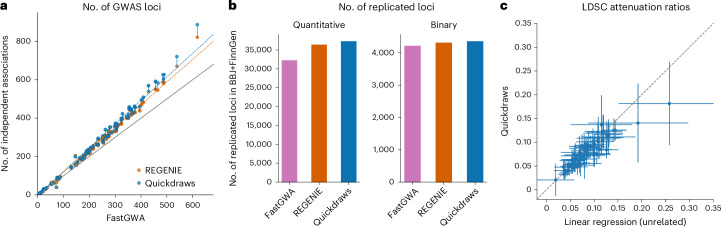
Power and calibration in real-data analysis. **a**, Comparison of the number of GWAS loci identified using Quickdraws, REGENIE and FastGWA. The vertical lines connect dots representing the same trait for Quickdraws and REGENIE (results including height are shown in Extended Data Fig. 3a). **b**, Total number of replicated loci in Biobank Japan (BBJ) using summary statistics from each method for quantitative and binary traits. **c**, Attenuation ratio of Quickdraws (*N* ≈ 405,000) versus linear regression in unrelated samples (*N* ≈ 337,000). The vertical and horizontal lines represent mean ± s.e. in the attenuation ratio estimate for each method.

We first assessed the number of approximately independent associations detected by each method using stringent clumping criteria (Methods). A summary of results for Quickdraws, REGENIE and FastGWA is shown in Fig. 2a; further data are reported in Supplementary Tables 6 and 7. The gains in detected associations were consistent with the power increases observed in simulations: Quickdraws found significantly more independent associations than REGENIE and FastGWA for quantitative and disease traits (binomial test *P* < 1.9 × 10^−3^) and performed similarly to BOLT-LMM for quantitative traits (Quickdraws = 26,236, BOLT-LMM = 26,368). For quantitative traits, Quickdraws detected 4.97% more independent associations than REGENIE and 22.71% more than FastGWA; for disease traits, Quickdraws found 3.25% more than REGENIE and 7.07% more than FastGWA-GLMM. The gains were larger in traits with high estimated heritability or low polygenicity^51^ (for example, 8.04% increase over REGENIE for mean platelet volume and 26.1% increase for standing height).

**Extended Data Fig. 3:**
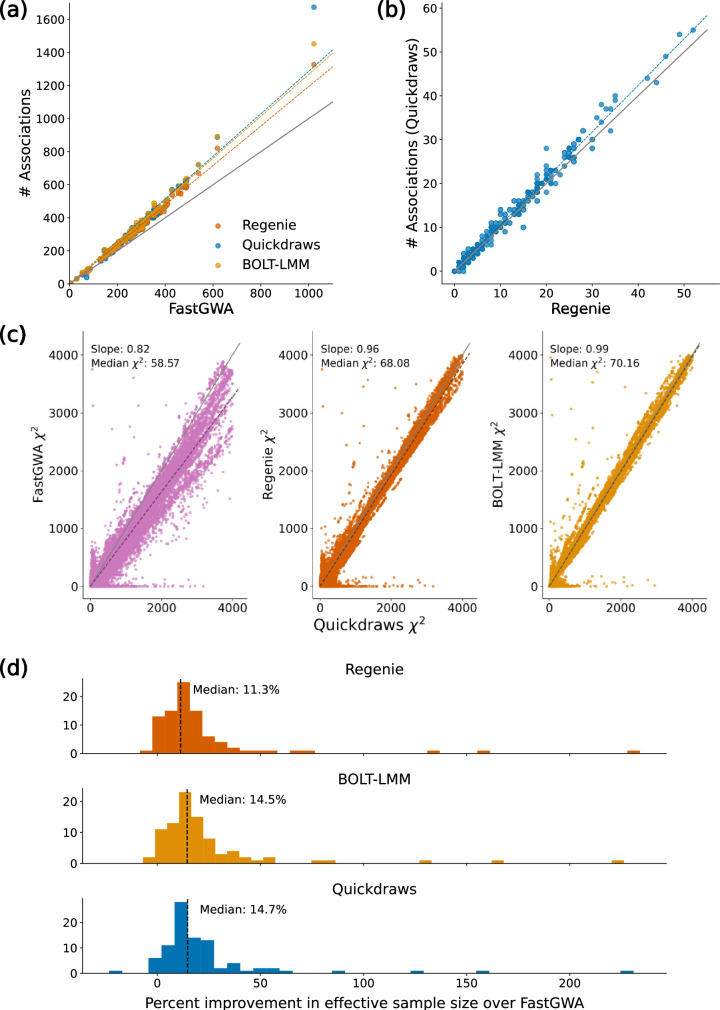
Approximately independent loci and effective sample size in UK Biobank analysis. (**a**) Number of approximately independent loci after plink clumping in FastGWA (x-axis) vs. Regenie, BOLT-LMM, and Quickdraws for 79 quantitative traits. (**b**) Number of approximately independent loci after plink clumping in Regenie (x-axis) vs. Quickdraws for 250 randomly sampled plasma protein traits. (**c**) The *χ*^2^ for 79 quantitative traits and N= 405k UK Biobank set conditioned on genome-wide significance (*p* = 5 × 10^−8^) in linear regression run on unrelated subset of the data (N= 337k). Median *χ*^2^: FastGWA = 58.57, Regenie = 68.08, BOLT-LMM = 70.16, Quickdraws = 70.64. (**d**) Histogram of the effective-sample size increase compared to FastGWA for 79 quantitative traits, measured as the mean *χ*^2^ minus 1 at genome-wide significant variants inferred though linear regression run on unrelated subset of the data (N= 337k). For (**a-c**), the gray line represents the y=x line and the dashed lines represent a linear regression fit for each method, for (**d**) the dashed line represents the median improvement in effective sample-size across traits. In (**c**), the slope refers to the linear regression slope of the *χ*^2^ values between each pair of methods.

We also analyzed 250 plasma protein traits (*N* ≈ 43,000; Methods). For these traits, which are less polygenic^52^, Quickdraws identified 5.54% more loci than REGENIE (*P* = 6.6 × 10^−3^; Extended Data Fig. 3b). Quickdraws’ effective sample size was 14.7% higher than FastGWA (*P* = 7.6 × 10^−4^), 3.4% higher than REGENIE (*P* = 0.197) and similar to BOLT-LMM (*P* = 0.46) (Extended Data Fig. 3c,d).

**Extended Data Fig. 4:**
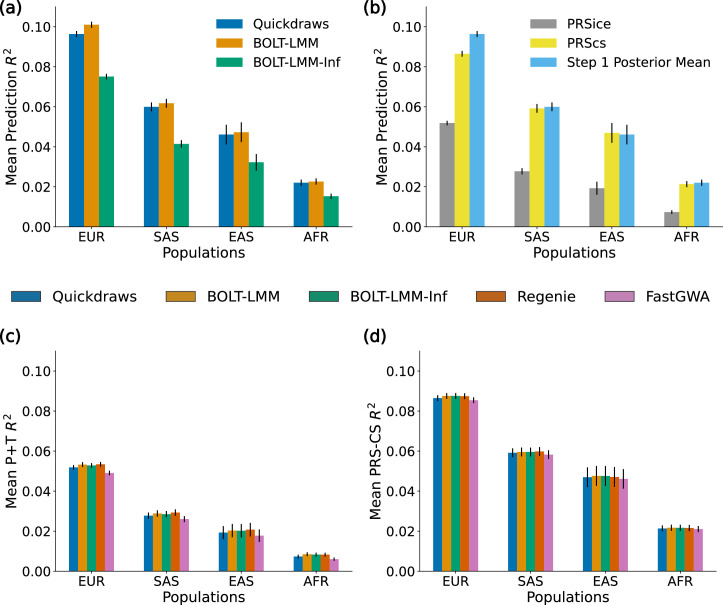
Phenotype prediction analyses in the UK Biobank. (**a**) Held-out mean phenotype prediction R^2^ comparing step 1 posterior estimates from Quickdraws, BOLT-LMM and BOLT-LMM-Inf. (**b**) Comparing Quickdraws’ step 1 posterior estimates with PGS calculated using Quickdraws’ association statistics and P+T (Pruning and thresholding as implemented in PRSice) or PRS-CS. (**c-d**) Comparing predictive power for PGS calculated using association statistics from different GWAS methods and different PGS methods, (**c**) P+T as implemented in PRSice and (**d**) PRS-CS. All analyses were performed on 27,683 held-out non-British Europeans, 9,044 self-identified south Asians, 1,457 self-identified east Asians and 7,204 self-identified African or African American samples. Results are aggregated across the 79 quantitative traits we analyzed, and the error bars represent 95% confidence interval of the mean prediction R^2^ for each method in each population subgroup.

To verify that these power gains result from modeling noninfinitesimal architectures, we evaluated the accuracy of polygenic predictions obtained during the model-fitting step from Quickdraws, BOLT-LMM and BOLT-LMM-Inf^21,22^. We used predictors from step 1, trained on ~405,000 white British individuals, to predict traits for the remaining samples. Across 79 quantitative traits, Quickdraws and BOLT-LMM obtained mean correlations of 0.307 (s.e. = 0.0061) and 0.313 (s.e. = 0.0061), whereas BOLT-LMM-Inf gave a lower correlation of 0.271 (s.e. = 0.0061) (Extended Data Fig. 4a). Similar improvements caused by the modeling of noninfinitesimal trait architectures have been observed in the context of polygenic prediction^21,22,53–55^.

We also compared predictions from step 1 of Quickdraws with polygenic scores (PGSs) built using recent methods^56,57^, based on summary statistics for 13.3 million variants. Quickdraws’ step 1 predictors were significantly more accurate (Student’s paired *t*-test *P* < 5.6 × 10^−6^) than PRS-CS and pruning and thresholding (P + T) in the European held-out set and had similar accuracy in other groups (Extended Data Fig. 4b). In addition, we compared the accuracy of PGS estimates built based on summary statistics obtained using different GWAS algorithms. Consistent with a recent analysis that did not find higher accuracy for PGSs built from summary statistics derived from noninfinitesimal modeling^58^, we did not observe significant differences across Quickdraws, BOLT-LMM or REGENIE. However, PGSs built using summary statistics from FastGWA were significantly less predictive (Student’s paired *t*-test *P* < 2.2 × 10^−4^ for both PRS-CS and P + T in European and south Asian held-out samples; Extended Data Fig. 4c,d). Finally, we tested the use of PGSs as covariates for association, a strategy recently shown to increase power^59–61^ (Methods and Supplementary Fig. 10). Consistent with recent results^59,61^, this approach increased FastGWA’s effective sample size. However, the effective sample size remained lower than that achieved by Quickdraws or BOLT-LMM and did not significantly change for REGENIE, which already accounts for polygenic effects by performing genome-wide regression.

**Extended Data Fig. 5:**
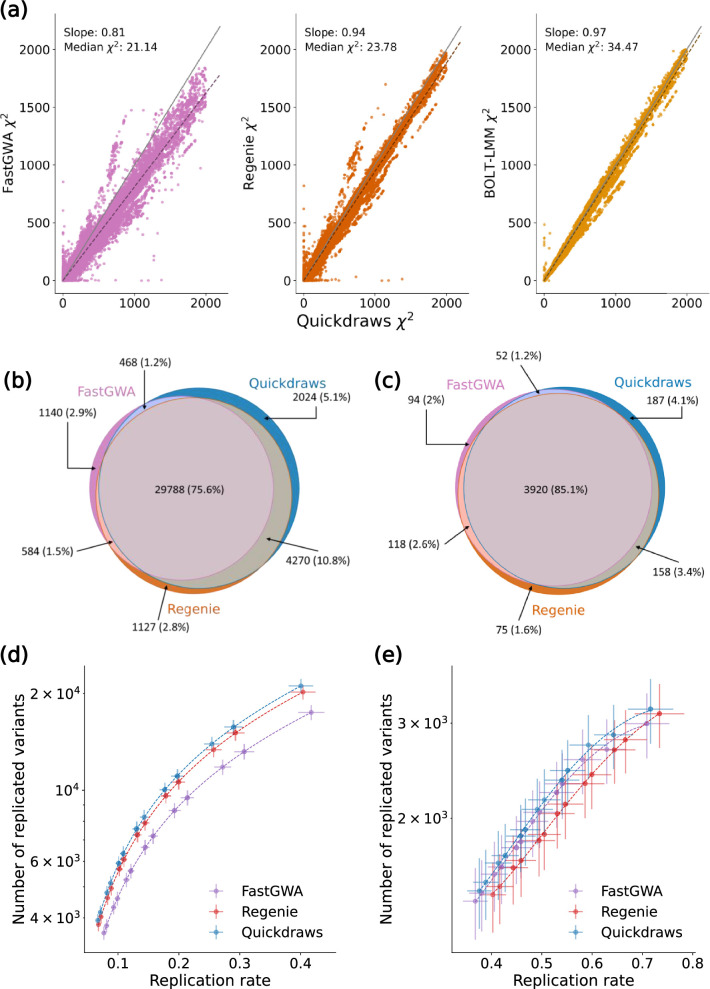
Replication analysis in Biobank Japan and Finngen. (**a**) The *χ*^2^ in UK Biobank conditioned on genome-wide significance (*p* = 5 × 10^−8^) in replicating biobank across GWAS methods. The variants are aggregated across traits, quantitative and binary traits for Regenie and FastGWA, and only quantitative traits for BOLT-LMM. The gray line represents the y = x line, and the dashed line the linear regression fit without intercept. Median *χ*^2^ for quantitative traits: FastGWA = 28.36, Regenie = 33.44, BOLT-LMM = 34.47 Quickdraws = 36.32; binary traits: FastGWA-GLMM = 14.08, Regenie = 14.34, Quickdraws = 14.51. (**b-c**) Replication Venn diagram for (**b**) 30 overlapping quantitative traits in Biobank Japan and (**c**) 23 overlapping binary traits in Biobank Japan and Finngen. (**d-e**) Number of replicated variants vs replication rate for (**d**) quantitative and (**e**) binary traits. The discovery threshold was fixed to 5 × 10^−9^ and the replication threshold was varied from 5 × 10^−2^ to 5 × 10^−8^. The error bars represent standard errors around the mean calculated using block jack-knife across chromosomes for 30 quantitative and 23 binary traits. The dashed line represents a cubic spline fit to the datapoints. In (**a**), the slope represents the linear regression slope of the *χ*^2^ values between methods, and in (**b-c**), the percentages indicate the proportion of associations found in the union of all methods.

We validated associations detected by Quickdraws through a replication analysis based on GWAS summary statistics from Biobank Japan^6^, FinnGen^10^ and other large-scale studies^62–66^ (Methods, Fig. 2b, Extended Data Fig. 5 and Supplementary Table 8). We estimated the number of UK Biobank variants also associated in the replication cohorts, using different significance thresholds. Across 53 traits in Biobank Japan and FinnGen (comprising 40 approximately independent traits), Quickdraws yielded a higher number of replicated loci than REGENIE and FastGWA (binomial tests *P* = 0.014 and *P* = 7 × 10^−4^). For 30 quantitative traits present in both datasets, Quickdraws yielded 2.5% and 15.72% more replicated loci than REGENIE and FastGWA, respectively, and a similar number to BOLT-LMM (Quickdraws = 37,210, BOLT-LMM = 37,072). For 23 overlapping disease traits, Quickdraws obtained 1.07% and 3.38% more replicated loci than REGENIE and FastGWA-GLMM, respectively. We observed similar gains in replication for five disease traits (Crohn’s disease^62^, type 2 diabetes^63^, celiac disease^64^, depression^65^ and ulcerative colitis^66^) where we had access to meta-analyzed summary statistics from large-scale studies (Supplementary Table 9). We verified that this increase in loci did not reduce replication rates across methods (Methods, Supplementary Tables 8 and 9 and Extended Data Fig. 5d,e). A Venn diagram depicting the relationships between sets of variants replicated using different methods is shown in Extended Data Fig. 5b,c; a summary of these results is shown in Fig. 2b. Note that these results may be affected by the choice of GWAS method used in the replication cohort.

Finally, we assessed calibration by comparing linkage disequilibrium (LD) score regression attenuation ratios^22^ for Quickdraws and linear regression applied to unrelated British samples^5^, across 79 quantitative traits. Quickdraws produced attenuation ratios close to those of linear regression (Quickdraws = 0.0832, s.e. = 0.008; linear regression = 0.0892, s.e. = 0.008; Fig. 2c). As linear regression is expected to be calibrated in this set, similar ratios for Quickdraws provide further evidence for its calibration^22,23^. Quickdraws also remained calibrated for low-prevalence binary traits, where it did not produce signatures of false-positive associations which are present in methods that do not perform test statistic adjustments such as saddle-point approximation^24^ or approximate Firth’s logistic regression^26^ (Supplementary Figs. 11–14). We further validated signals found using Quickdraws but not using REGENIE by evaluating the functional annotation of the regions where these variants were found. We found similar enrichments compared with variants detected using both REGENIE and Quickdraws (Supplementary Figs. 15 and 16), indicating no major differences in the functional profile of variants exclusively detected by Quickdraws.

### Computational costs

We compared the computational efficiency of Quickdraws with REGENIE, FastGWA, SAIGE and BOLT-LMM in large-scale UK Biobank analyses, testing the speed and cost of each method on the UK Biobank RAP. We analyzed ~13.3 million imputed genotypes for 50 quantitative and 50 binary traits, varying sample sizes between *N* = 50,000 and *N* = 405,088 (Methods). We used 458,464 markers for model fitting for Quickdraws, REGENIE and BOLT-LMM and 89,177 markers for SAIGE, and used a pre-computed sparse genomic relationship matrix (GRM) for FastGWA and SAIGE (Methods and Supplementary Note). Unlike current approaches, Quickdraws leverages GPU hardware to speed up Bayesian regression in the model-fitting step. To provide a detailed overview of costs and optimize hardware, we tested all methods on up to four RAP machines (Methods). Quickdraws also offers high-memory and low-memory modes for model fitting. The high-memory mode loads the genotype matrix in memory, whereas the low-memory mode streams data from disk, reducing memory usage but running 20% slower on the RAP instance that we tested. Tables 1 and 2 report the minimal cost for each method across RAP machines, along with running time and memory usage on a common machine, using the high-memory option for Quickdraws.

**Table 1:** Computational efficiency for binary trait association

Samples	Method	Step 1	Step 2	Total time	Total memory^a^	Cost on RAP^b^
		(h)	(h)	(h)	(GB)	(£)
50,000	Quickdraws	18.3	77.8	96.1	49 (16)	25.2
	SAIGE	2.1	270.9	273	<15	47.5
	REGENIE	13.1	168.9	182.0	<15	41.0
	FastGWA-GLMM	–	40.6	40.6	<15	9.2
405,088	Quickdraws	162.7	519.7	682.3	67 (16)	395.9
	SAIGE	9.0	12,015.5	12,024.4	<15	2,834.9
	REGENIE	56	813.9	869.9	<15	506.9
	FastGWA-GLMM	–	254.1	254.1	<15	98.1

We compared the computational requirements for recent GWAS algorithms with Quickdraws to generate summary statistics for 13.3 million variants and 50 binary traits with either *N* = 50,000 or *N* = 405,088. ^a^Total memory includes CPU RAM memory and GPU memory; only Quickdraws requires GPU memory which is reported separately in brackets. ^b^A more detailed cost analysis can be found in Supplementary Table 12. Running times are computed using the same hardware for all methods (mem2_ssd1_v2_x8, with 32 GB of RAM, 8-core processor for *N* = 50,000 and mem1_ssd1_v2_x36, 72 GB of RAM, 36-core processor for *N* = 405,088 datasets).

Table 2 summarizes results for 50 quantitative traits and *N* ≈ 405,000, where Quickdraws computed association statistics in 149.3 h at a cost of £93. We extrapolated that BOLT-LMM, with similar power, would need 56.2× more time and 80.6× more cost for the same traits. REGENIE required 50.0 h at £24.2, whereas FastGWA required 128.4 h at £37.3. For 50 binary traits (Table 1), Quickdraws yielded the highest power, computing association statistics for ~13.3 million variants in 682.3 h and for £395.9. REGENIE with Firth’s regression fallback needed 869.9 h and £506.8, whereas SAIGE with pre-computed sparse GRM took 12,024.4 h and £2,834.9. Although FastGWA-GLMM provided the lowest power, it was the least expensive, running in 254.1 h and £98.1. In high-memory mode, Quickdraws’ maximum random access memory (RAM) usage scales with *MN*/4, similar to BOLT-LMM. It also requires additional GPU memory, depending on the number of analyzed traits and the batch size. REGENIE and FastGWA required <15 GB of RAM for the analysis of ~405,000 samples, although REGENIE also needed ~355 GB of disk space for intermediate files.

**Table 2:** Computational efficiency for quantitative trait association

Samples	Method	Step 1	Step 2	Total time	Total memory^a^	Cost on RAP^b^
		(h)	(h)	(h)	(GB)	(£)
50,000	Quickdraws	9.6	9	18.6	48 (16)	7.9
	BOLT-LMM	127	671.5	798.5	<15	158.4
	REGENIE	1.1	19.4	20.5	<15	4.0
	FastGWA	–	20.2	20.2	<15	3.6
405,088	Quickdraws	97.7	51.5	149.3	63 (16)	93.0
	BOLT-LMM	1,150	7,250	8,400	46	7,500.0
	REGENIE	4.7	45.3	50.0	<15	24.2
	FastGWA	–	128.4	128.4	<15	37.3

We compared the computational requirements for recent GWAS algorithms with Quickdraws to generate summary statistics for 13.3 million variants and 50 quantitative traits with either *N* = 50,000 or *N* = 405,088. ^a^Total memory includes CPU RAM memory and GPU memory; only Quickdraws requires GPU memory which is reported separately in brackets. ^b^A more detailed cost analysis can be found in Supplementary Table 13. Running times are computed using the same hardware for all methods (mem2_ssd1_v2_x8, with 32 GB of RAM, 8-core processor for *N* = 50,000 and mem1_ssd1_v2_x36, 72 GB of RAM, 36-core processor for *N* = 405,088 datasets).

We finally compared the computational cost for sample sizes up to one million individuals, extrapolating costs for testing up to 600 million variants, and found similar costs for Quickdraws and REGENIE (Supplementary Table 10). We also compared the cost of single-trait analyses for *N* ≈ 405,000 samples (Supplementary Table 11). Although FastGWA does not support multi-trait analyses, it was substantially faster for single-trait analyses than Quickdraws and REGENIE, which are optimized for multi-trait processing.

## Discussion

To fully harness the growing volumes of genomic, phenotypic and environmental data in modern biobanks, GWAS algorithms need to optimize the trade-off across cost efficiency, statistical power and robustness. We developed Quickdraws, an algorithm that addresses these trade-offs by combining established GWAS paradigms, such as mixed-effect models^12–23^ and penalized regression^26,67^, with Bayesian machine learning techniques, such as stochastic variational inference^32,33^, first-order gradient optimizers^34,35^ and transfer learning^36^. Quickdraws also leverages GPUs, which are widely used in machine learning and increasingly available in modern computing platforms, to parallelize matrix operations^68^. This allows Quickdraws to achieve higher power for binary traits and match BOLT-LMM’s power for quantitative traits, the only other approach modeling noninfinitesimal architectures, while requiring fewer computational resources. In UK Biobank RAP benchmarks, Quickdraws required a fraction of the resources used by BOLT-LMM, with costs comparable to other scalable methods with lower average association power. The association statistics that we computed for 79 quantitative traits, 50 diseases and 2,923 plasma protein traits are publicly available (‘Data availability’).

The power gains from Quickdraws are equivalent to analyzing larger sample sizes^21,22,39^, with simulations showing >19.2% increases in effective sample size for traits linked to a few thousand causal variants. In real-trait analyses, Quickdraws identified more independent signals than other scalable methods for both highly polygenic traits (for example, 26.1% and 8.04% increases over REGENIE for height and mean platelet volume) and less polygenic molecular traits. As the range of phenotypic measurements in biobank datasets expands, we expect these gains to extend to a broader set of traits.

Our analyses show that, in addition to increasing association power through the computation of a residualized phenotype, posterior mean effect size estimates from the model-fitting step can be used to construct PGSs that perform well in held-out individuals, outperforming those based on summary statistics. Therefore, sharing these effect estimates, which we make available for the traits we analyzed (‘Data availability’), may facilitate downstream analyses. However, unlike summary association statistics, using posterior effect estimates for PGSs lacks established methods to combine them across cohorts. Developing strategies to meta-analyze these effects across cohorts could open new avenues for federated association and polygenic prediction strategies that preserve high statistical power without the need for sharing individual-level data.

We highlight several current limitations and areas of future work. First, Quickdraws relies on GPUs to speed up Bayesian regression, making it slower on CPU hardware (Supplementary Fig. 17). Although GPUs have higher costs (Tables 1 and 2), our analyses on the UK Biobank RAP show that the overall increase in running costs is small. Moreover, their widespread use in machine learning is likely to increase availability and reduce costs. Second, Quickdraws does not yet capture correlations across traits, which could improve performance^69,70^. Third, although adjusting for PGSs within the same sample did not lead to power gains comparable to those achieved by Quickdraws and BOLT-LMM, using scores from larger external cohorts could lead to further gains^60,61^. Fourth, association analyses have recently been shown to be affected by participation biases^71,72^, to which Quickdraws is also susceptible. We implemented an adjustment strategy from ref. ^73^ in step 2 (Methods), but further work is needed to integrate it into step 1. Despite these current limitations and avenues for future development, we believe that Quickdraws will provide a useful tool for large-scale GWASs, demonstrating the promise of leveraging modern machine learning methodology to improve statistical power and efficiency in these analyses.

## Methods

### Ethics

UK Biobank data were analyzed after approval of UK Biobank proposal no. 43206.

### Bayesian regression with spike-and-slab prior

Quickdraws uses a Bayesian regression to estimate the genetic effects. This Bayesian regression uses a spike-and-slab distribution on the variant effects, which results in increased association power compared with approaches that assume an infinitesimal model and rely on a Gaussian prior. We optimized the posterior using stochastic variational inference^33^, which substantially improved the scalability of this approach. A brief introduction to variational inference and additional details on the optimization techniques used by Quickdraws are provided in Supplementary Note.

#### Quantitative traits

Quickdraws adopts the following Bayesian linear regression model for a quantitative trait:

1 $$\begin{array}{lr}{\mathrm{Likelihood}}:&P({\mathbf{y}}| X,\mathbf{\upbeta}) \sim {\mathcal{N}}({X \mathbf{\upbeta}},{\sigma }_{e}^{2}),\\ {\mathrm{Prior}}:&P(\mathbf{\upbeta})=\mathop{\prod }\limits_{j}P({\upbeta }_{j}),P({\upbeta }_{j}) \sim (1-{p}_{0}){\mathcal{N}}(0,{\sigma }^{2})+{p}_{0}\delta (0),\\ {\mathrm{Approximate}}\; {\mathrm{posterior}}:&q(\mathbf{\upbeta})=\mathop{\prod }\limits_{j}q({\upbeta }_{j}),q({\upbeta }_{j}) \sim (1-{\psi }_{j}){\mathcal{N}}({\mu }_{j},{\sigma }_{j}^{2})+{\psi }_{j}\delta (0).\end{array}$$

In the above expression, given *N* individuals and *M* variants, *X* is an *N* × *M* covariate-adjusted genotype matrix, **y** an *N* × 1 covariate-adjusted phenotype vector and **β** an *M* × 1 vector of variant effects. *δ*(0) is the Dirac delta function at 0, representing the spike in the spike-and-slab prior used to model the sparsity in the genetic architecture. The spike-and-slab prior was recently shown to be conjugated to the normal likelihood^74^, making it a natural choice for variational inference. We also assumed a fully factorized spike-and-slab approximate posterior, which leads to efficient and low-variance sampling from the posterior. Next, we used stochastic variational inference to directly optimize the evidence lower bound (ELBO). The ELBO for Bayesian linear regression with spike-and-slab prior, normal likelihood and spike-and-slab approximate posterior (derived in Supplementary Note) can be approximated as follows:

2 $$\begin{array}{lll}{L}_{{\mathrm{VI}}}^{Q}(\mathbf{\uppsi} ,\mathbf{\upmu} ,\mathbf{\upsigma})&\approx &-\mathop{\sum }\limits_{b=1}^{B}\left(\mathop{\sum }\limits_{s=1}^{S}\frac{{({{\mathbf{y}}}_{b}- {X}_{b}\mathbf{\upbeta} (\mathbf{s}))}^{2}}{2{\sigma }_{e}^{2}}+\right.\\ &&+\frac{1}{B}\mathop{\sum }\limits_{j=1}^{M}\left(\frac{{\psi }_{j}}{2}\left(-1+\frac{{\mu }_{j}^{2}+{\sigma }_{j}^{2}}{{\sigma }^{2}}-\log \frac{{\sigma }_{j}^{2}}{{\sigma }^{2}}\right)\right.\\&&\left.\left.+(1-{\psi }_{j})\log \frac{1-{\psi }_{j}}{1-{p}_{0}}+{\psi }_{j}\log \frac{{\psi }_{j}}{{p}_{0}}\right)\right).\end{array}$$

In this expression, ${L}_{{\mathrm{VI}}}^{Q}(\mathbf{\uppsi} ,\mathbf{\upmu} ,\mathbf{\upsigma} )$ is the variational inference objective to be maximized, **ψ**, **μ**, **σ** are the variational parameters to be optimized, *X*_*b*_ and **y**_*b*_ are a genotype matrix and a phenotype vector containing the *b*th (out of *B*) batch of the data, batched along the number of samples, whereas **β**(**s**) are the effect estimates sampled from the approximate posterior *q*(**β**). Note that, contrary to other variational inference schemes, we aimed to optimize a stochastic objective, because **β**(**s**) is randomly sampled. To reduce the variance of this objective, which leads to faster convergence, we relied on a local reparameterization trick^75^ and the use of antithetic variates^76^. We provided additional details on algorithmic techniques that we employed for variance reduction in Supplementary Note. We also implemented several performance optimizations to improve the computational efficiency of this step, such as transferring the effect estimates trained on whole-genome data to a LOCO run (Supplementary Fig. 18) and employing efficient data storage with two-bit encoding per genotype. For further details on these optimizations, refer to Supplementary Note.

#### Binary traits

For binary traits, we adopted a Bayesian logistic regression model, so that the likelihood of equation (1) becomes:

3 $$P(\;\mathbf{y}| X,\mathbf{\upbeta})=\mathop{\prod }\limits_{n=1}^{N}{c}_{n}^{\;{y}_{n}}{\left\{1-{c}_{n}\right\}}^{1-{y}_{n}},{c}_{n}=\sigma \left({{\mathbf{X}}}_{n}{\mathbf{\upbeta}}\right),$$

although the prior and approximate posterior are unchanged. In the above, **X**_*n*_ and *y*_*n*_ represent the covariate-adjusted genotype vector and phenotype values for the *n*th individual, *σ* is the sigmoid function, which maps the output of the regression to a value between 0 and 1, and all other quantities are as previously defined. We also assumed that the approximate posterior has a spike-and-slab distribution; although it is not conjugated to the logistic distribution, we observed it to provide a good approximation to the true posterior. The ELBO, which we again optimized using stochastic variational inference, now takes the form:

4 $$\begin{array}{lll}{L}_{{\mathrm{VI}}}^{{\mathrm{Bi}}}(\mathbf{\uppsi} ,\mathbf{\upmu} ,\mathbf{\upsigma})&\approx &-\mathop{\sum }\limits_{b=1}^{B}\left(\mathop{\sum }\limits_{s=1}^{S}{\mathbf{y}}_{b}\log (\sigma ({{X}}_{b}\,\mathbf{\upbeta }(\mathbf{s})))+(1-{\mathbf{y}}_{b})\log (1-\sigma ({{X}}_{b}\,\mathbf{\upbeta }(\mathbf{s})))+\right.\\ &&+\frac{1}{B}\mathop{\sum }\limits_{j=1}^{M}\left(\frac{{\psi }_{j}}{2}\left(-1+\frac{{\mu }_{j}^{2}+{\sigma }_{j}^{2}}{{\sigma }^{2}}-\log \frac{{\sigma }_{j}^{2}}{{\sigma }^{2}}\right)+(1-{\psi }_{j})\log \frac{1-{\psi }_{j}}{1-{p}_{0}}\right.\\&&\left.\left.+{\psi }_{j}\log \frac{{\psi }_{j}}{{p}_{0}}\right)\right),\end{array}$$

where all terms are defined as in equation (2).

### Heritability estimation

To set the prior variance and likelihood contribution in equations (1) and (3), we computed an estimate of the narrow-sense heritability for the trait of interest. For quantitative traits, we estimated heritability using a parallelized implementation of the RHE algorithm^28,29^ (PyPI v.1.0) which allows the simultaneous estimation of variance components for multiple traits^30^. This approach requires a single pass through the data and allows estimating heritability in linear time in *N* and *M*. We set the number of random RHE-mc vectors to 50 while assigning markers to 8 components based on their LD scores and MAF. We also excluded the human leukocyte antigen (HLA) region, as recommended in ref. ^29^. For binary traits, we performed a grid search over a set of heritability values, *h*^2^ ∈ {0.01, 0.25, 0.5, 0.75}, running the Bayesian regression for each value and selecting the heritability corresponding to the highest likelihood.

### Test statistics calculation

After estimating the random genetic effects as described earlier in Methods, we computed association statistics for quantitative or binary traits as follows.

#### Quantitative traits

We modeled the phenotype of interest as a combination of fixed and random effects, which include genetic effects and environmental effects:

5 $${\mathbf{y}}= C\boldsymbol{\alpha} +{{\mathbf{x}}}_{{\mathrm{test}}}{\beta }_{{\mathrm{test}}}+g+\epsilon ,$$

where **y** is an *N* × 1 vector of phenotype values, **x**_test_ is an *N* × 1 vector of variant values, *C* is an *N* × *C* matrix of C covariates, and *g* and *ϵ* are random genetic and environmental effects. Note that using a covariate-adjusted genotype and phenotype by first regressing out any covariates is equivalent to including covariates as fixed effects in the above equation. Based on this model, we aimed to compute the following *χ*^2^ association statistic (a derivation is provided in Supplementary Note):

6 $$\frac{{\left({{\mathbf{x}}}_{{\mathrm{test}}}^{T}{\hat{V}}^{-1}{\mathbf{y}}\right)}^{2}}{{{\mathbf{x}}}_{{\mathrm{test}}}^{T}{\hat{V}}^{-1}{{\mathbf{x}}}_{{\mathrm{test}}}} \sim {\chi }_{1}^{2},$$

where $\hat{V}$ is the estimated variance matrix, defined as ${\hat{V}}=\frac{{{\hat{\sigma }_{g}}}^{2}}{M}{X}_{{\mathrm{GRM}}}{X}_{{\mathrm{GRM}}}^{T}+{{\hat{\sigma }_{e}}}^{2}{I}_{N}$, ${{\hat{\sigma }_{g}}}^{2}$ and ${{\hat{\sigma }_{e}}}^{2}$ are estimated genetic and environmental variance components and *X*_GRM_ is the genotype matrix used for model fitting. Obtaining the test statistic of equation (6) requires calculating the inverse of an *N* × *N* matrix, which creates a computational bottleneck for large sample sizes. This test statistic can be linked to the best linear unbiased predictor of a trait, by re-writing equation (6) as described in ref. ^21^:

7 $$\frac{{\left({{\mathbf{x}}}_{{\mathrm{test}}}^{T}\mathbf{\tilde{y}}/{\sigma }_{e}^{2}\right)}^{2}}{{{\mathbf{x}}}_{{\mathrm{test}}}^{T}{\hat{V}}^{-1}{{\mathbf{x}}}_{{\mathrm{test}}}} \sim {\chi }_{1}^{2}.$$

In the above, $\mathbf{\tilde{y}}$ is a residual phenotype estimated using Bayesian linear regression. We adopted a similar strategy and replaced the $\mathbf{\tilde{y}}$ with $\mathbf{{\tilde{y}}_{{\mathrm{LOCO}}}}$, the LOCO residual phenotype from Bayesian regression. In addition, the denominator ${{\mathbf{x}}}_{{\mathrm{test}}}^{T}{\hat{V}}^{-1}{{\mathbf{x}}}_{{\mathrm{test}}}$ of equation (7) can be shown to be well approximated using a constant multiple of ${{\mathbf{x}}}_{{\mathrm{test}}}^{T}{{\mathbf{x}}}_{{\mathrm{test}}}$ (ref. ^37^), further reducing computational costs. We can therefore write the test statistics for a quantitative trait up to a constant of proportionality as:

8 $${\chi }_{Q}^{2}\propto \frac{{\left({{\mathbf{x}}}_{{\mathrm{test}}}^{T}\mathbf{{\tilde{y}}_{{\mathrm{LOCO}}}}\right)}^{2}}{{{\mathbf{x}}}_{{\mathrm{test}}}^{T}{{\mathbf{x}}}_{{\mathrm{test}}}}.$$

To calibrate these statistics, we found the constant of proportionality by matching an estimate of the effective sample size, defined as mean *χ*^2^ test statistic, minus 1 (ref. ^39^). To this end, we estimated the increase in the effective sample size owing to use of a Bayesian linear regression approach, as well as the decrease in effective sample size resulting from the presence of relatedness among individuals. The improvement in sample size owing to the use of Bayesian linear regression is estimated in step 1 of the algorithm, by calculating the held-out residual phenotypic variance^21,22,77^. Relatedness is accounted for as done in ref. ^77^, by considering the effects of relationships of up to third degree. We use this information to compute a correction factor that aims to match the average *χ*^2^ test statistic of equation (8) for Quickdraws and that obtained by applying linear regression on homogeneous unrelated individuals. We provide additional algorithmic details on the calculation and calibration of statistics in Supplementary Note.

#### Binary traits

For binary traits, we modeled the association between genotype and phenotype using a logistic mixed model:

9 $${\mathrm{logit}}({p}_{i})= C\boldsymbol{\alpha}+{{\mathbf{x}}}_{{\mathrm{test}}}{\beta }_{{\mathrm{test}}}+g+\epsilon ,$$

where *p*_*i*_ = *P*(*y*_*i*_ = 1∣**x**_test_, *g*, *C*) is the probability of the *i*th individual being a case given **x**_test_, covariates *C* and random genetic effects *g*. A score test statistic for the null hypothesis *β*_test_ = 0 is then computed as $T={{\mathbf{x}}}_{{\mathrm{test}}}^{T}(\mathbf{y}-\mathbf{\hat{p}})$, where $\mathbf{\hat{p}}$ is the estimated mean under the null model. The normalized test statistic for logistic mixed model can then be written as:

10 $$T=\frac{{{\mathbf{x}}}_{{\mathrm{test}}}^{T}(\mathbf{y}-\mathbf{\hat{p}})}{\sqrt{{{\mathbf{x}}}_{{\mathrm{test}}}^{T}\hat{P}{{\mathbf{x}}}_{{\mathrm{test}}}}},$$

where $\hat{P}={\hat{V}}^{-1}-{\hat{V}}^{-1}C{({C}^{T}{\hat{V}}^{-1}C)}^{-1}{C}^{T}{\hat{V}}^{-1}$ is a dense *N* × *N* matrix with ${\hat{V}}=\frac{{{\hat{\sigma }_{g}}}^{2}}{M}{X}_{{\mathrm{GRM}}}{X}_{{\mathrm{GRM}}}^{T}+{\hat{W}}^{-1}$ and $\hat{W}={\mathrm{diag}}\{\hat{p}(1-\hat{p})\}$. Similar to quantitative traits, the test statistic in equation (9) can be written up to a constant of proportionality^24^ as:

11 $${T}_{B}\propto \frac{{{\mathbf{x}}}_{{\mathrm{test}}}^{T}(\mathbf{y}-\mathbf{\hat{p}})}{\sqrt{{{\mathbf{x}}}_{{\mathrm{test}}}^{T}\hat{W}{{\mathbf{x}}}_{{\mathrm{test}}}}},$$

where $\hat{W}$ is a diagonal matrix (with diagonal entries $\hat{{p}_{i}}(1-\hat{{p}_{i}})$) from the predictions of the null model in step 1. The test statistic from equation (11) is assumed to be normally distributed, which is often not the case with binary traits that have low prevalence or for rare variants. We therefore instead performed Firth’s logistic regression, which penalizes the likelihood by using a Jeffreys’ prior, removing most of the asymptotic bias in maximum-likelihood estimation. To save computation, we used the likelihood ratio test with an approximate version of Firth’s logistic regression introduced in ref. ^26^ on variants below a *P*-value threshold of 0.05. We further narrowed down the list of variants by only applying Firth’s logistic regression to rare variants (default MAF < 5%) or rare traits (default prevalence <5%) or both. Finally, similarly to quantitative traits, we calibrated the test statistics by matching the estimated scaled effective sample size with that of a logistic regression model run on an unrelated homogeneous subset of the data.

### UK Biobank analyses

We used the UK Biobank SNP array data for 405,088 white British samples^5^ (in PLINK bed format^44,45^) for model fitting and HRC + UK10,000-imputed data^5,78,79^ (in bgen v.1.2 format^80^) for association testing. We filtered the set of available autosomal variants to have MAF ≥ 1%, Hardy–Weinberg equilibrium *P* ≥1 × 10^−15^ and a genotyping rate >99%, obtaining a set of 458,620 markers. We used these markers as input for the model-fitting step of BOLT-LMM (v.2.4.1), REGENIE (v.3.1.1) and Quickdraws, whereas for SAIGE (v.1.1.6) we performed LD pruning using PLINK (v.1.9 (refs. ^44,45^) setting –window_size to 50 kb, –step_size to 5 and the *R*^2^ threshold to 0.05) as recommended, which resulted in 89,177 markers. We computed association statistics on a much larger set of ~13.3 million imputed variants with MAF ≥ 0.1% and INFO score ≥0.8.

For our main analyses, we considered 79 quantitative traits (comprising blood-related, anthropometric and other traits) and 50 binary self-reported disease traits. The traits we selected had a phenotyping rate >80% and, for quantitative traits, a statistically significant estimated narrow-sense heritability (*P* < 5 × 10^−4^, using LD-score regression estimates available at https://nealelab.github.io/UKBB_ldsc/h2_browser.html). Quantitative traits were also standardized, mean centered and quantile normalized to have an approximately Gaussian distribution. All methods that we considered were provided with the top 20 PCs^5^, age, sex, age^2^, age × sex, age^2^ × sex and smoking status as covariates during model fitting and testing.

We additionally considered 2,923 plasma protein traits from the UK Biobank, which we pre-processed as done in refs. ^52,81^. We downloaded normalized protein expression values from the UK Biobank RAP (field 30900), with measurements for up to 53,074 participants. For GWASs, we considered up to 49,441 European participants (based on self-reported field 21000). We ran Quickdraws with covariates including the top 20 PCs^5^, age, sex, age^2^, age × sex, age^2^ × sex, smoking status, collection site, batch and time difference between blood sampling and protein measurement. We also ran REGENIE on 250 randomly sampled plasma proteins, focusing on a subset of *N* = 43,293 individuals overlapping the British ancestry subgroup described in ref. ^5^. Association was performed in independent batches of 250 traits in parallel.

### Simulations

To assess the robustness and power of Quickdraws and other methods, we performed simulations with varying levels of relatedness and population stratification. We used SNP array data from the UK Biobank, using all autosomal variants with MAF ≥ 0.01%, Hardy–Weinberg equilibrium *P* ≥ 1 × 10^−15^ and a genotyping rate >99%, obtaining 512,828 variants. Of these, 54,568 had an MAF between 10^−4^ and 10^−2^. We either randomly sampled 50,000 individuals to assemble groups of samples matching specific relatedness and population structure criteria, or considered the entire subset of white British individuals (*N* ≈ 405,000). In each setting, we simulated 50 quantitative and 50 binary traits with narrow-sense heritability ${h}_{g}^{2}=0.4$, a realistic MAF dependence (*α* = −0.3)^42,43^ and varying polygenicity and prevalence levels.

We also simulated phenotypes generated using different distributions of genetic effects, including spike-and-slab, mixture of Gaussians, Laplace and Gaussian. To simulate effects following a spike-and-slab prior, given a polygenicity value *p* and *M* variants, we randomly selected *Mp* causal variants (out of *M* = 512,828 variants) and randomly sampled their effect sizes from a Gaussian distribution. To facilitate the calculation of FPRs and assess calibration, we chose only causal variants from the odd chromosomes, setting the effect sizes of variants on even chromosomes to zero. We simulated population stratification and shared environment between close relatives using:

12 $$\begin{array}{rcl}{y}_{i}&=&\mathop{\sum }\limits_{j=1}^{Mp}{G}_{i,\,j}{\beta }_{j}+A\gamma +\tau +\epsilon ,\\ {\beta }_{j}& \sim &{\mathcal{N}}\left(0,\frac{{h}_{g}^{2}{c}_{f}}{Mp}\right), \epsilon \sim {\mathcal{N}}\left(0,{h}_{e}^{2}\right),\end{array}$$

where $\mathop{\sum }\nolimits_{j=1}^{Mp}{G}_{i,\,j}{\beta }_{j}$ is a genetic effect explaining 40% of trait variance, *Aγ* a population stratification term determined using self-reported ancestry, *τ* the shared environmental effect, simulated by assigning a Gaussian draw to closely related individuals, and *ϵ* the residual environmental effect, with variance equal to ${h}_{e}^{2}$. To simulate from a mixture-of-Gaussians prior, we added an extra parameter *f* ∈ 0.05, 0.5, which controls the fraction of variance explained by the Gaussian with smaller variance. For Laplace and Gaussian priors, we assumed polygenicity *p* = 1, and sampled the effects *β*_*j*_ in equation (12) from either distribution.

In our simulations, we set the variance explained due to population stratification to be 5% of trait variance, whereas sample relatedness explained 10% of the trait variance in second-degree relatives and 20% in first-degree relatives^23^. To simulate a realistic MAF-dependent trait architecture, we set *c*_*f*_ ∝ (2*f*(1 − *f*))^*α*^, where *f* is the MAF and *α* = −0.3 (refs. ^42,43^). For binary traits, we adopted a liability threshold model by synthesizing a quantitative phenotype as in equation (12) and setting individuals above or below a given prevalence value as cases or controls. We varied the prevalence in binary traits from 0.3 to 0.001 and the amount of relatedness and population structure based on the following four simulation conditions, listed below.

#### Unrelated white British (GB-unrel)

We randomly sampled 50,000 individuals from the set of unrelated white British as defined in ref. ^5^. This scenario does not include population structure or relatedness.

#### Related white British (GB-rel)

We included 25,000 individuals from the set of individuals in the GB-rel but not in the GB-unrel subset, along with 25,000 unrelated white British individuals. This resulted in 552 first-degree, 906 second-degree and 1,396 third-degree relative pairs (3.4× more relative pairs than randomly sampling from the GB-rel subset). We also considered less and more extreme levels of relatedness, assembling sets of samples by randomly sampling from the GB-rel subset (referred to as GB-rel-ukb) and including high proportions of first- and second-degree relatives (referred as GB-rel+ having 7.3× and 4.8× more first- and second-degree pairs than randomly sampling from the GB-rel subset).

#### European structure (EUR)

To simulate higher levels of population structure in the dataset, we included 25,000 non-British European samples^5^ along with 25,000 unrelated British samples. This set also comprised 451 first-degree and 107 second-degree relative pairs, corresponding to 1.2× and 0.8× the amount of first- and second-degree pairs found in the GB-rel-ukb.

#### Pan-ancestry structure

To simulate population structure comprising multiple ancestry descriptors found in the UK Biobank, we included 7,500 self-reported African samples, 7,500 self-reported south Asian samples, along with 35,000 unrelated British samples, as defined in ref. ^5^. This set comprised 425 first-degree and 173 second-degree relative pairs, corresponding to 1.1× and 1.2× more first- and second-degree pairs than randomly sampling from the GB-rel-ukb subset. We simulated correlated variant effects across ancestry groups using a multivariate Gaussian, setting the covariance of effects between Africans and other subgroups to 0.4, and between the European and south Asian subgroups to 0.7 (ref. ^82^).

### Cost analysis on UK Biobank RAP

We performed an extensive cost analysis on the UK Biobank RAP cloud platform. We compared the computational requirements and cost of running Quickdraws with BOLT-LMM, REGENIE and FastGWA for quantitative traits, and SAIGE, REGENIE (with Firth regression fallback) and FastGWA-GLMM for binary traits. Note that, although we used FastGWA-GLMM^25^ in all analyses involving binary traits, we occasionally refer to it as FastGWA for brevity. As each GWAS method has different computational requirements and bottlenecks, we evaluated the running time and costs on multiple RAP hardware configurations, reporting the minimal cost for each method in Tables 1 and 2. For *N* = 50,000, we considered mem3_ssd1_v2_x4, mem2_ssd1_v2_x8, and mem1_ssd1_v2_x16; for *N* ≈ 405,000, we considered mem2_ssd1_v2_x8, mem2_ssd1_v2_x16, mem1_ssd1_v2_x36 and mem1_ssd1_v2_x72; and for *N* = 1,000,000, we considered only mem2_ssd1_v2_x32. We additionally used mem2_ssd2_gpu1_x8 to run step 1 of Quickdraws on an A10G GPU for all the experiments. Note that we did not exhaustively test all possible hardware configurations available on RAP. More details about these analyses can be found in Supplementary Note; numerical values and a comparison between methods are provided in Supplementary Tables 12 and 13 and Supplementary Fig. 19, respectively.

### Replication analyses using Biobank Japan and FinnGen association statistics

We assessed the number of variants detected using different GWAS methods that can be replicated in the Biobank Japan and FinnGen datasets, using downloaded summary association statistics (‘Data availability’). We considered 30 quantitative traits and 13 self-reported disease traits from Biobank Japan, as well as 21 self-reported disease traits from FinnGen for which equivalent definitions were available in both our analyses and the replication dataset and for which at least one significant association was detected in both studies. We considered variants with MAF ≥ 0.1%, INFO score ≥ 0.8 and appearing in both datasets, leading to ~5.89 million variants available for Biobank Japan replication and ~10.89 million variants available for FinnGen replication.

We separately considered the replication of associated variants and loci. For the replication of individual variants, we assessed the number (and proportion) of variants detected (*P*_dis_ ≤ 5 × 10^−9^) in the UK Biobank that are also significant in Biobank Japan, using multiple replication thresholds, *P*_rep_ ≤ 5 × 10^−2^, *P*_rep_ ≤ 5 × 10^−4^ and *P*_rep_ ≤ 5 × 10^−6^. For the replication of loci, we followed a procedure similar to that adopted in ref. ^83^, defining a credible set for a locus to contain a lead (or sentinel) associated variant together with additional proxy variants found within a 50-kb window from the lead variant and with association significance *P* ≤ 100 × *P*_sentinel_. We defined a locus as replicated if any variant in the credible set was also found to be associated (at *P* ≤ 5 × 10^−2^) with the same direction of effect in the replication cohort. To ensure consistent replication rates across different GWAS methods, we used a two-step procedure. First, we calculated the average replication rate for each trait using all GWAS methods (FastGWA, REGENIE and Quickdraws) with a discovery threshold of 5 × 10^−9^. Then, we adjusted the discovery threshold for each method, varying it from 2 × 10^−9^ to 8 × 10^−9^, to achieve the same replication rate for each method. We performed a similar replication analysis with publicly available summary statistics for five disease traits (Crohn’s disease^62^, type 2 diabetes^63^, celiac disease^64^, depression^65^ and ulcerative colitis^66^) that have higher number of cases than in the UK Biobank (Supplementary Table 9).

### Functional enrichment analysis

We used the baseline-LD model annotations^84,85^ to assess the distribution of functional annotations for sets of variants associated using different methods. We defined the functional enrichment for a set of variants as the proportion of variants being in a particular functional category divided by the genome-wide fraction of variants assigned to that category. We considered binary and quantitative traits separately and estimated the mean functional enrichment across phenotypes by computing the ratio between the number of variants from a given set that belong to a functional category (numerator) and the total number of variants in the set (denominator). We estimated this ratio by separately summing the numerator and denominator terms across all analyzed phenotypes. We estimated s.e. values around the enrichment by applying the jackknife method to 50 equally sized genome blocks. We looked at functional enrichment for various sets of variants, including: (1) variants found in Quickdraws but not in REGENIE and (2) variants found in both Quickdraws and REGENIE. For (2), we matched the *χ*^2^ distribution of the two considered sets, sampling variants detected using REGENIE so that they approximately match the empirical *χ*^2^ distribution of the variants detected using Quickdraws.

### PGS analyses

We constructed PGSs for all the 79 quantitative traits analyzed, using either P + T as implemented in PRSice^56^ or the PRS-CS method^57^. We used summary statistics generated with various methods on the set of ~13.3 million imputed variants described earlier to generate PGSs for held-out individuals from European, south Asian, east Asian and African subgroups of the UK Biobank. We compared the predictive performance of the PGS methods (P + T and PRS-CS) using summary statistics computed using FastGWA, REGENIE, BOLT-LMM, BOLT-LMM-Inf and Quickdraws. We reported the mean predictive *R*^2^ and the 95% confidence interval across all analyzed traits, obtained using a meta-analysis of Fisher-transformed estimated correlation coefficients. We also evaluated a PGS constructed by applying posterior mean effect estimates computed for 458,620 SNPs during step 1 of the Quickdraws algorithm. We compared these PGSs based on posterior mean effects with PGSs estimated using summary statistics generated for the same samples using the full Quickdraws algorithm.

We also performed experiments in which a within-cohort PGS is used as a covariate to adjust for background polygenic effects^59–61^, focusing on chromosomes 1–5, using up to 405,088 samples from the white British subgroup and analyzing all 79 quantitative traits. We first generated LOCO PGSs, as described in ref. ^59^, using PRS-CS and summary statistics computed with FastGWA or REGENIE. We then used FastGWA and REGENIE to test for association again, this time including the LOCO PGSs corresponding to the chromosome of the variant being tested as a covariate, during both the model-fitting and the association steps.

### Correction of participation bias

Quickdraws implements functionality to optionally correct for participation bias when computing association statistics in step 2, using an approach closely related to the one proposed in ref. ^73^. This approach relies on the use of sampling weights, which are computed by considering a range of covariates and used to up-weight or down-weight individuals estimated to be under-sampled or over-sampled in the study^73^. Quickdraws takes these weights as input and performs a weighted linear regression, using a Huber–White estimator to compute the variance of the fixed effects in the regression. The test statistics are recalibrated using a weighted linear regression, by matching the effective sample size estimated from a weighted linear regression on an unrelated and homogeneous sample, similar to the unweighted case. We tested this approach in 79 quantitative traits and 338,738 individuals from the white British subgroup for which all the covariates used in ref. ^73^ to estimate sampling weights were available (Supplementary Fig. 20). These covariates included sex, age, education age, alcohol consumption frequency, smoking status, income, household size, employment status, body mass index, overall health, height, urbanization, weight, assessment center, self-reported ethnicity and years of education. We used the LASSO regression model provided in ref. ^73^ to estimate these sampling weights and compared the number of approximately independent loci identified using Quickdraws versus LDAK (v.5.2).

### Reporting summary

Further information on research design is available in the Nature Portfolio Reporting Summary linked to this article.

## Online content

Any methods, additional references, Nature Portfolio reporting summaries, source data, extended data, supplementary information, acknowledgements, peer review information; details of author contributions and competing interests; and statements of data and code availability are available at 10.1038/s41588-024-02044-7.

## Supplementary information

Supplementary Information Supplementary Note, Tables 2–7 and 9–14 and Figs. 1–20.

Reporting Summary

Supplementary Tables Supplementary Tables 1 and 8.

## Data Availability

Summary statistics generated using Quickdraws for quantitative, binary and plasma protein traits are available at https://www.stats.ox.ac.uk/publication-data/sge/ppg/quickdraws and have been deposited in the GWAS Catalog (https://www.ebi.ac.uk/gwas/) under accession numbers GCST90468059–GCST90471107. The individual-level genotype and phenotype data are available to approved researchers through the UK Biobank (http://www.ukbiobank.ac.uk). Summary association statistics for Biobank Japan were downloaded from https://pheweb.jp. Summary association statistics for FinnGen were obtained, following approval, through https://www.finngen.fi/en/access_results. Baseline-LD annotations and HapMap3 filtered European LD scores for comparing attenuation ratios were downloaded from https://alkesgroup.broadinstitute.org/LDSCORE.

## Code availability

The Quickdraws software package (v.0.1.0) is freely available at https://palamaralab.github.io/software/quickdraws. The code used for the main analyses in the present study is available via Zenodo at 10.5281/zenodo.13936055 (ref. ^86^). External software used in the current study was obtained from the following URLs: REGENIE (v.3.1.1) https://rgcgithub.github.io/regenie; PLINK (v.1.9) https://www.cog-genomics.org/plink2; BOLT-LMM (v.2.4.1) https://alkesgroup.broadinstitute.org/BOLT-LMM; FastGWA (v.1.94.1) https://yanglab.westlake.edu.cn/software/gcta; FastGWA-GLMM (v.1.94.1) https://yanglab.westlake.edu.cn/software/gcta; SAIGE (v.1.1.6) https://saigegit.github.io/SAIGE-doc; RHE (PyPI v.1.0.0) https://pypi.org/project/rhe; LDSC software (v.1.0.0) https://github.com/bulik/ldsc; LDAK software (v.5.2) http://dougspeed.com/downloads2; Python package PySnpTools (v.0.5.10) https://github.com/fastlmm/PySnpTools; Python package PyTorch (v.2.4.0) https://pytorch.org; Python package Numba (v.0.55.1) https://numba.pydata.org; and Python package Numpy (v.2.1.2) https://numpy.org.

## References

[1] Abdellaoui, A., Yengo, L., Verweij, K. J. & Visscher, P. M. 15 years of GWAS discovery: realizing the promise. *Am. J. Human Genet.* **110**, 179–194 (2023). 10.1016/j.ajhg.2022.12.011 PMC9943775 36634672

[2] Khera, A. V. et al. Genome-wide polygenic scores for common diseases identify individuals with risk equivalent to monogenic mutations. *Nat. Genet.* **50**, 1219–1224 (2018). 30104762 10.1038/s41588-018-0183-z PMC6128408

[3] Craig, J. E. et al. Multitrait analysis of glaucoma identifies new risk loci and enables polygenic prediction of disease susceptibility and progression. *Nat. Genet.* **52**, 160–166 (2020). 31959993 10.1038/s41588-019-0556-y PMC8056672

[4] Klarin, D. & Natarajan, P. Clinical utility of polygenic risk scores for coronary artery disease. *Nat.Rev. Cardiol.* **19**, 291–301 (2022). 34811547 10.1038/s41569-021-00638-w PMC10150334

[5] Bycroft, C. et al. The uk biobank resource with deep phenotyping and genomic data. *Nature* **562**, 203–209 (2018). 30305743 10.1038/s41586-018-0579-z PMC6786975

[6] Nagai, A. et al. Overview of the biobank japan project: study design and profile. *J. Epidemiol.* **27**, S2–S8 (2017). 28189464 10.1016/j.je.2016.12.005 PMC5350590

[7] Ramirez, A. H. et al. The all of us research program: data quality, utility, and diversity. *Patterns* **3**, 100570 (2022). 10.1016/j.patter.2022.100570 PMC9403360 36033590

[8] Wojcik, G. L. et al. Genetic analyses of diverse populations improves discovery for complex traits. *Nature* **570**, 514–518 (2019). 31217584 10.1038/s41586-019-1310-4 PMC6785182

[9] Chen, Z. et al. China Kadoorie Biobank of 0.5 million people: survey methods, baseline characteristics and long-term follow-up. *Int. J. Epidemiol.* **40**, 1652–1666 (2011). 10.1093/ije/dyr120 PMC3235021 22158673

[10] Kurki, M. I. et al. Finngen provides genetic insights from a well-phenotyped isolated population. *Nature* **613**, 508–518 (2023). 36653562 10.1038/s41586-022-05473-8 PMC9849126

[11] Zhou, W. et al. Global biobank meta-analysis initiative: powering genetic discovery across human disease. *Cell Genom.* **2**, 100192 (2022). 36777996 10.1016/j.xgen.2022.100192 PMC9903716

[12] Yu, J. et al. A unified mixed-model method for association mapping that accounts for multiple levels of relatedness. *Nat. Genet.* **38**, 203–208 (2006). 16380716 10.1038/ng1702

[13] Kang, H. M. et al. Efficient control of population structure in model organism association mapping. *Genetics* **178**, 1709–1723 (2008). 18385116 10.1534/genetics.107.080101 PMC2278096

[14] Kang, H. M. et al. Variance component model to account for sample structure in genome-wide association studies. *Nat. Genet.* **42**, 348–354 (2010). 20208533 10.1038/ng.548 PMC3092069

[15] Zhang, Z. et al. Mixed linear model approach adapted for genome-wide association studies. *Nat. Genet.* **42**, 355–360 (2010). 20208535 10.1038/ng.546 PMC2931336

[16] Zhou, X. & Stephens, M. Genome-wide efficient mixed-model analysis for association studies. *Nat. Genet.* **44**, 821–824 (2012). 22706312 10.1038/ng.2310 PMC3386377

[17] Lippert, C. et al. Fast linear mixed models for genome-wide association studies. *Nat. Methods* **8**, 833–835 (2011). 21892150 10.1038/nmeth.1681

[18] Segura, V. et al. An efficient multi-locus mixed-model approach for genome-wide association studies in structured populations. *Nat. Genet.* **44**, 825–830 (2012). 22706313 10.1038/ng.2314 PMC3386481

[19] Listgarten, J. et al. Improved linear mixed models for genome-wide association studies. *Nat. Methods* **9**, 525–526 (2012). 22669648 10.1038/nmeth.2037 PMC3597090

[20] Listgarten, J., Lippert, C. & Heckerman, D. Fast-lmm-select for addressing confounding from spatial structure and rare variants. *Nat. Genet.* **45**, 470–471 (2013). 23619783 10.1038/ng.2620

[21] Loh, P.-R. et al. Efficient Bayesian mixed-model analysis increases association power in large cohorts. *Nat. Genet.* **47**, 284–290 (2015). 10.1038/ng.3190 PMC4342297 25642633

[22] Loh, P.-R., Kichaev, G., Gazal, S., Schoech, A. P. & Price, A. L. Mixed-model association for biobank-scale datasets. *Nat. Genet.* **50**, 906–908 (2018). 29892013 10.1038/s41588-018-0144-6 PMC6309610

[23] Jiang, L. et al. A resource-efficient tool for mixed model association analysis of large-scale data. *Nat. Genet.* **51**, 1749–1755 (2019). 31768069 10.1038/s41588-019-0530-8

[24] Zhou, W. et al. Efficiently controlling for case-control imbalance and sample relatedness in large-scale genetic association studies. *Nat. Genet.* **50**, 1335–1341 (2018). 30104761 10.1038/s41588-018-0184-y PMC6119127

[25] Jiang, L., Zheng, Z., Fang, H. & Yang, J. A generalized linear mixed model association tool for biobank-scale data. *Nat. Genet.* **53**, 1616–1621 (2021). 34737426 10.1038/s41588-021-00954-4

[26] Mbatchou, J. et al. Computationally efficient whole-genome regression for quantitative and binary traits. *Nat. Genet.* **53**, 1097–1103 (2021). 34017140 10.1038/s41588-021-00870-7

[27] Yengo, L. et al. A saturated map of common genetic variants associated with human height. *Nature* **610**, 704–712 (2022). 36224396 10.1038/s41586-022-05275-y PMC9605867

[28] Wu, Y. & Sankararaman, S. A scalable estimator of SNP heritability for biobank-scale data. *Bioinformatics* **34**, i187–i194 (2018). 10.1093/bioinformatics/bty253 PMC6022682 29950019

[29] Pazokitoroudi, A. et al. Efficient variance components analysis across millions of genomes. *Nat. Commun.* **11**, 4020 (2020). 10.1038/s41467-020-17576-9 PMC7419517 32782262

[30] Zhu, J. et al. Fast variance component analysis using large-scale ancestral recombination graphs. Preprint at *bioRxiv*10.1101/2024.08.31.610262 (2024).

[31] Yang, J., Zaitlen, N. A., Goddard, M. E., Visscher, P. M. & Price, A. L. Advantages and pitfalls in the application of mixed-model association methods. *Nat. Genet.* **46**, 100–106 (2014). 24473328 10.1038/ng.2876 PMC3989144

[32] Graves, A. Practical variational inference for neural networks. *Adv. Neural Inform. Process. Systems* **24**, 2348–2356 (2011).

[33] Hoffman, M. D., Blei, D. M., Wang, C. & Paisley, J. Stochastic variational inference. *J. Machine Learn. Res.* **14**, 1303–1347 (2013).

[34] Robbins, H. & Monro, S. A stochastic approximation method. *Ann. Math. Statistics* **22**, 400–407 (1951).

[35] Kingma, D. P. & Ba, J. Adam: A method for stochastic optimization. Preprint at https://arxiv.org/abs/1412.6980 (2014).

[36] Pan, S. J. & Yang, Q. A survey on transfer learning. *IEEE Trans. Knowl. Data Eng.* **22**, 1345–1359 (2009).

[37] Svishcheva, G. R., Axenovich, T. I., Belonogova, N. M., Van Duijn, C. M. & Aulchenko, Y. S. Rapid variance components–based method for whole-genome association analysis. *Nat. Genet.* **44**, 1166–1170 (2012). 22983301 10.1038/ng.2410

[38] Jakobsdottir, J. & McPeek, M. S. Mastor: mixed-model association mapping of quantitative traits in samples with related individuals. *Am. J. Human Genet.* **92**, 652–666 (2013). 23643379 10.1016/j.ajhg.2013.03.014 PMC3644644

[39] Yang, J. et al. Genomic inflation factors under polygenic inheritance. *Eur. J. Hum. Genet.* **19**, 807–812 (2011). 10.1038/ejhg.2011.39 PMC3137506 21407268

[40] Lam, S. K., Pitrou, A. & Seibert, S. Numba: A llvm-based python jit compiler. In *Proc. Second Workshop on the LLVM Compiler Infrastructure in HPC* (ed. Finkel, H.) 1–6 (ACM, 2015).

[41] Speed, D., Hemani, G., Johnson, M. R. & Balding, D. J. Improved heritability estimation from genome-wide SNPs. *Am. J. Hum. Genet.* **91**, 1011–1021 (2012). 10.1016/j.ajhg.2012.10.010 PMC3516604 23217325

[42] Zeng, J. et al. Signatures of negative selection in the genetic architecture of human complex traits. *Nat. Genet.* **50**, 746–753 (2018). 29662166 10.1038/s41588-018-0101-4

[43] Schoech, A. P. et al. Quantification of frequency-dependent genetic architectures in 25 UK Biobank traits reveals action of negative selection. *Nat. Commun.* **10**, 790 (2019). 30770844 10.1038/s41467-019-08424-6 PMC6377669

[44] Purcell, S. et al. Plink: a tool set for whole-genome association and population-based linkage analyses. *Am. J. Hum. Genet.* **81**, 559–575 (2007). 10.1086/519795 PMC1950838 17701901

[45] Chang, C. C. et al. Second-generation PLINK: rising to the challenge of larger and richer datasets. *Gigascience* **4**, s13742–015-0047-8 (2015). 10.1186/s13742-015-0047-8 PMC4342193 25722852

[46] Stahl, E. A. et al. Bayesian inference analyses of the polygenic architecture of rheumatoid arthritis. *Nat. Genet.* **44**, 483–489 (2012). 22446960 10.1038/ng.2232 PMC6560362

[47] O’Connor, L. J. et al. Extreme polygenicity of complex traits is explained by negative selection. *Am. J. Hum. Genet.* **105**, 456–476 (2019). 10.1016/j.ajhg.2019.07.003 PMC6732528 31402091

[48] Haworth, S. et al. Apparent latent structure within the UK Biobank sample has implications for epidemiological analysis. *Nat. Commun.* **10**, 333 (2019). 30659178 10.1038/s41467-018-08219-1 PMC6338768

[49] Sohail, M. et al. Polygenic adaptation on height is overestimated due to uncorrected stratification in genome-wide association studies. *eLife* **8**, e39702 (2019). 30895926 10.7554/eLife.39702 PMC6428571

[50] Berg, J. J. et al. Reduced signal for polygenic adaptation of height in UK Biobank. *eLife* **8**, e39725 (2019). 30895923 10.7554/eLife.39725 PMC6428572

[51] Zeng, J. et al. Widespread signatures of natural selection across human complex traits and functional genomic categories. *Nat. Commun.* **12**, 1164 (2021). 33608517 10.1038/s41467-021-21446-3 PMC7896067

[52] Sun, B. B. et al. Plasma proteomic associations with genetics and health in the UK Biobank. *Nature* **622**, 329–338 (2023). 37794186 10.1038/s41586-023-06592-6 PMC10567551

[53] Vilhjálmsson, B. J. et al. Modeling linkage disequilibrium increases accuracy of polygenic risk scores. *Am. J. Hum. Genet.* **97**, 576–592 (2015). 10.1016/j.ajhg.2015.09.001 PMC4596916 26430803

[54] Lloyd-Jones, L. R. et al. Improved polygenic prediction by Bayesian multiple regression on summary statistics. *Nat. Commun.* **10**, 5086 (2019). 10.1038/s41467-019-12653-0 PMC6841727 31704910

[55] Privé, F., Arbel, J. & Vilhjálmsson, B. J. Ldpred2: better, faster, stronger. *Bioinformatics* **36**, 5424–5431 (2020). 10.1093/bioinformatics/btaa1029 PMC8016455 33326037

[56] Euesden, J., Lewis, C. M. & O’reilly, P. F. Prsice: polygenic risk score software. *Bioinformatics* **31**, 1466–1468 (2015). 25550326 10.1093/bioinformatics/btu848 PMC4410663

[57] Ge, T., Chen, C.-Y., Ni, Y., Feng, Y.-C. A. & Smoller, J. W. Polygenic prediction via Bayesian regression and continuous shrinkage priors. *Nat. Commun.* **10**, 1776 (2019). 10.1038/s41467-019-09718-5 PMC6467998 30992449

[58] Weissbrod, O. et al. Leveraging fine-mapping and multipopulation training data to improve cross-population polygenic risk scores. *Nat. Genet.* **54**, 450–458 (2022). 35393596 10.1038/s41588-022-01036-9 PMC9009299

[59] Bennett, D., O’Shea, D., Ferguson, J., Morris, D. & Seoighe, C. Controlling for background genetic effects using polygenic scores improves the power of genome-wide association studies. *Sci. Rep.* **11**, 19571 (2021). 34599249 10.1038/s41598-021-99031-3 PMC8486788

[60] Campos, A. I. et al. Boosting the power of genome-wide association studies within and across ancestries by using polygenic scores. *Nat. Genet.* **55**, 1769–1776 (2023). 37723263 10.1038/s41588-023-01500-0

[61] Jurgens, S. J. et al. Adjusting for common variant polygenic scores improves yield in rare variant association analyses. *Nat. Genet.* **55**, 544–548 (2023). 36959364 10.1038/s41588-023-01342-w PMC11078202

[62] Jostins, L. et al. Host–microbe interactions have shaped the genetic architecture of inflammatory bowel disease. *Nature* **491**, 119–124 (2012). 23128233 10.1038/nature11582 PMC3491803

[63] Morris, A. P. et al. Large-scale association analysis provides insights into the genetic architecture and pathophysiology of type 2 diabetes. *Nat. Genet.* **44**, 981–990 (2012). 10.1038/ng.2383 PMC3442244 22885922

[64] Dubois, P. C. et al. Multiple common variants for celiac disease influencing immune gene expression. *Nat. Genet.* **42**, 295–302 (2010). 20190752 10.1038/ng.543 PMC2847618

[65] Nagel, M. et al. Meta-analysis of genome-wide association studies for neuroticism in 449,484 individuals identifies novel genetic loci and pathways. *Nat. Genet.* **50**, 920–927 (2018). 29942085 10.1038/s41588-018-0151-7

[66] De Lange, K. M. et al. Genome-wide association study implicates immune activation of multiple integrin genes in inflammatory bowel disease. *Nat. Genet.* **49**, 256–261 (2017). 28067908 10.1038/ng.3760 PMC5289481

[67] Firth, D. Bias reduction of maximum likelihood estimates. *Biometrika* **80**, 27–38 (1993).

[68] Ansel, J. et al. Pytorch 2: faster machine learning through dynamic python bytecode transformation and graph compilation. In *Proc. 29th ACM International Conference on Architectural Support for Programming Languages and Operating Systems* (eds Abu-Ghazaleh, N. et al.) 929–947 (ACM, 2024).

[69] Korte, A. et al. A mixed-model approach for genome-wide association studies of correlated traits in structured populations. *Nat. Genet.* **44**, 1066–1071 (2012). 22902788 10.1038/ng.2376 PMC3432668

[70] Zhou, X. & Stephens, M. Efficient multivariate linear mixed model algorithms for genome-wide association studies. *Nat. Methods* **11**, 407–409 (2014). 24531419 10.1038/nmeth.2848 PMC4211878

[71] Pirastu, N. et al. Genetic analyses identify widespread sex-differential participation bias. *Nat. Genet.* **53**, 663–671 (2021). 33888908 10.1038/s41588-021-00846-7 PMC7611642

[72] Benonisdottir, S. & Kong, A. Studying the genetics of participation using footprints left on the ascertained genotypes. *Nat. Genet.* **55**, 1413–1420 (2023). 37443256 10.1038/s41588-023-01439-2 PMC10412458

[73] Schoeler, T. et al. Participation bias in the UK Biobank distorts genetic associations and downstream analyses. *Nat. Hum. Behav.* **7**, 1216–1227 (2023). 10.1038/s41562-023-01579-9 PMC10365993 37106081

[74] Spence, J. Flexible mean field variational inference using mixtures of non-overlapping exponential families. *Adv. Neural Inf. Process. Syst.* **33**, 19642–19654 (2020).

[75] Kingma, D. P., Salimans, T. & Welling, M. Variational dropout and the local reparameterization trick. In *Proc. 28th International Conference**on Neural Information Processing Systems* (eds Cortes, C. et al.) 2575–2583 (Curran Associates Inc., 2015).

[76] Hammersley, J. & Morton, K. A new Monte Carlo technique: antithetic variates. In *Mathematical Proc. Cambridge Philosophical Society* 449–475 (Cambridge Univ. Press, 1956).

[77] Ziyatdinov, A. et al. Estimating the effective sample size in association studies of quantitative traits. *G3* **11**, jkab057 (2021). 33734375 10.1093/g3journal/jkab057 PMC8495748

[78] The Haplotype Reference Consortium A reference panel of 64,976 haplotypes for genotype imputation. *Nat. Genet.* **48**, 1279–1283 (2016). 10.1038/ng.3643 PMC5388176 27548312

[79] The UK10K Consortium The UK10K project identifies rare variants in health and disease. *Nature* **526**, 82–90 (2015). 10.1038/nature14962 PMC4773891 26367797

[80] Band, G. & Marchini, J. Bgen: a binary file format for imputed genotype and haplotype data. Preprint at *bioRxiv*10.1101/308296 (2018).

[81] Dhindsa, R. S. et al. Rare variant associations with plasma protein levels in the UK Biobank. *Nature* **622**, 339–347 (2023). 37794183 10.1038/s41586-023-06547-x PMC10567546

[82] Ruan, Y. et al. Improving polygenic prediction in ancestrally diverse populations. *Nat. Genet.* **54**, 573–580 (2022). 35513724 10.1038/s41588-022-01054-7 PMC9117455

[83] Huang, Q. Q. et al. Transferability of genetic loci and polygenic scores for cardiometabolic traits in British Pakistani and Bangladeshi individuals. *Nat. Commun.* **13**, 4664 (2022). 35945198 10.1038/s41467-022-32095-5 PMC9363492

[84] Finucane, H. K. et al. Partitioning heritability by functional annotation using genome-wide association summary statistics. *Nat. Genet.* **47**, 1228–1235 (2015). 26414678 10.1038/ng.3404 PMC4626285

[85] Gazal, S. et al. Linkage disequilibrium-dependent architecture of human complex traits shows action of negative selection. *Nat. Genet.* **49**, 1421–1427 (2017). 28892061 10.1038/ng.3954 PMC6133304

[86] Loya, H., Kalantzis, G., Cooper, F. & Palamara, P. F. Quickdraws. *Zenodo*10.5281/zenodo.13936055 (2024).

