# Supplementary material for: A scalable variational inference approach for increased mixed-model association power
Source: Nat Genet. 2025 Jan 9;57(2):461–8. doi: 10.1038/s41588-024-02044-7 (PMC11821521; doi:10.1038/s41588-024-02044-7)
Supplement: Supplementary file 1 — Supplementary Note, Tables 2–7 and 9–14 and Figs. 1–20. [file 41588_2024_2044_MOESM1_ESM.pdf]

nature genetics

Supplementary information

https://doi.org/10.1038/s41588-024-02044-7

A scalable variational inference approach for increased mixed-model association power

In the format provided by the authors and unedited

Contents

Supplementary Note **2**

1 Background **2**
1.1 Linear mixed models . . . 2
1.1.1 Leave-one-chromosome-out . . . 2
1.1.2 Calculation of the test statistic . . . 2
1.1.3 Infinitesimal model . . . 3
1.1.4 Non-infinitesimal model . . . 4
1.2 Bayesian inference . . . 4
1.3 Variational inference . . . 4
1.3.1 Evidence lower bound . . . 5
1.3.2 Mean-field variational inference . . . 5
1.3.3 Coordinate ascent variational inference . . . 6
1.3.4 Stochastic variational inference . . . 7

2 Quickdraws: Algorithm details **10**
2.1 Preprocessing . . . 10
2.2 Bayesian regression . . . 10
2.2.1 Optimizing the stochastic VI objective . . . 10
2.2.2 ELBO variance reduction . . . 10
2.2.3 Hyperparameter tuning . . . 12
2.3 Calculation of test statistics . . . 12

3 Performance optimization **15**
3.1 Memory optimization . . . 15
3.2 Speed optimization . . . 15

4 Summary of the Quickdraws algorithm **16**

5 Cost analysis on the UK Biobank RAP **17**

6 Data analyses and significance testing **20**

References **21**

Appendix **23**

Supplementary Tables **24**

Supplementary Figures **37**

Supplementary Note

1 Background

1.1 Linear mixed models

Linear mixed models (LMMs) are an extension of linear models that facilitate the analysis of non-independent data. They are commonly adopted in the analysis of genomic data involving population structure or cryptic relatedness. LMMs handle non-independence by modeling random effects that capture variability within different subpopulations or phenotypic correlation across close relatives. In an LMM for genome-wide association analysis, the phenotype of interest is often modeled as a combination of fixed effects, which include the effects of the test variant and covariates, and random effects, which include genetic effects and environmental effects:

$$y = x_{test}\beta_{test} + \alpha C + g + \epsilon. \tag{1}$$

In the above, y is an $N \times 1$ vector of mean-centered and standardized phenotype values, x_{test} is an $N \times 1$ vector of mean-centered and standardized biallelic variant values (i.e., the original $\tilde{x}_{test}$ takes values in $\{0, 1, 2\}^N$ for diploid samples), C is an $N \times C$ matrix of covariates of interest, such as principal components, age, and sex. We are mainly interested in inferring the fixed effect of a particular variant, β_{test}, given the random genetic effects, which help capture the effects of population structure and relatedness. We model the genetic and environmental effects using

$$\begin{aligned} g &= \beta_{GRM} X_{GRM} \\ \epsilon &\sim \mathcal{N}(0, \sigma_e^2), \end{aligned} \tag{2}$$

where X_{GRM} is an $N \times M_{GRM}$ standardized genotype matrix and σ_e^2 is the environmental variance for a given phenotype. For simplicity, the environmental random effect is assumed to be independent and identically distributed (i.i.d.) from a normal distribution, and the random genetic effect is modeled as a weighted sum of standardized genotypes. The random genetic effect g can also be thought of as a sample from $\mathcal{N}(0, \sigma_g^2 K)$, where σ_g^2 is the genetic variance for a given phenotype and $K = \frac{1}{M} X_{GRM} X_{GRM}^T$ is the genetic relatedness matrix.

1.1.1 Leave-one-chromosome-out

It has been observed that in order to avoid reducing the power of association, the matrix X_{GRM} should not contain the test variant x_{test}, as well as any other variants in linkage disequilibrium (LD) with x_{test}. This problem has been referred to as "proximal contamination" [1]. Fitting an LMM with genetic effects from variants not in LD with the testing variant, however, would require re-training the model to infer genetic effects for every tested variant, which would cause major computational overheads. [2] proposed a simple solution to this problem, referred to as the leave-one-chromosome-out (LOCO) scheme, where the X_{GRM} is constructed using all variants except those on the same chromosome as the test variant. For example, assuming 22 autosomal chromosomes and a test variant on chromosome 7, X_{GRM} is built to contain all variants from chromosome 1-6 and 6-22. This approach, which requires training a separate model for each chromosome, allows circumventing proximal contamination while increasing association power, and has been widely adopted by recent LMM association algorithms [3–5].

1.1.2 Calculation of the test statistic

To infer the fixed effect β_{test} in Equation 1, one can marginalize the random effects $\beta_{GRM} X_{GRM}$ and ϵ to get the marginalized log-likelihood:

$$\begin{aligned} y \mid x_{test}, \beta_{test}, \sigma_e^2, \sigma_g^2 &\sim \mathcal{N}(x_{test}\beta_{test}, \frac{\sigma_g^2}{M} X_{GRM} X_{GRM}^T + \sigma_e^2 I_N) \\ \log P(y \mid x_{test}, \beta_{test}, \sigma_e^2, \sigma_g^2) &\propto -\frac{1}{2}(y - x_{test}\beta_{test})^T V^{-1}(y - x_{test}\beta_{test}) - \frac{1}{2}|\log(V)|, \end{aligned} \tag{3}$$

where σ_g^2 and σ_e^2 are genetic and environmental variance components as described above, and V is a covariance matrix, defined as $V = \frac{\sigma_g^2}{M} X_{GRM} X_{GRM}^T + \sigma_e^2 I_N$. The maximum likelihood estimate (MLE) for β_{test} is obtained by setting $\frac{\partial \log P(y|x_{test}, \beta_{test})}{\partial \beta_{test}} = 0$; the variance of the estimate is obtained using the inverse Fisher information $-E\left[\frac{\partial^2 \log P(y|x_{test}, \beta_{test})}{\partial \beta_{test}^2} \mid \hat{\beta}_{test}\right]^{-1}$ and takes the form

$$\hat{\beta}_{test} = (x_{test}^T V^{-1} x_{test})^{-1}(x_{test}^T V^{-1} y)$$
$$var(\hat{\beta}_{test}) = (x_{test}^T V^{-1} x_{test})^{-1}. \quad (4)$$

The variance components, σ_g^2 and σ_e^2, are estimated using restricted likelihood maximization (REML) [3] or moment-based methods [6, 7] rather than standard maximum likelihood estimation. In this work, we estimate the variance components $\hat{\sigma}_g^2$ and $\hat{\sigma}_e^2$ using the RHE-MC algorithm [6, 7], a highly scalable moment-based approach that was recently extended to allow processing multiple traits in parallel [8]. We then estimate the covariance matrix using $\hat{V} = \frac{\hat{\sigma}_g^2}{M} X_{GRM} X_{GRM}^T + \hat{\sigma}_e^2 I_N$, and the effects using

$$\hat{\beta}_{test} = (x_{test}^T \hat{V}^{-1} x_{test})^{-1}(x_{test}^T \hat{V}^{-1} y),$$
$$var(\hat{\beta}_{test}) = (x_{test}^T \hat{V}^{-1} x_{test})^{-1}. \quad (5)$$

Using the asymptotic property of the MLE,

$$\frac{\hat{\beta}_{test} - \beta_{test}}{\sqrt{var(\hat{\beta}_{test})}} \sim \mathcal{N}(0, 1) \quad (6)$$

To test the null hypothesis $\beta_{test} = 0$, we therefore substitute the MLE estimate and the variance of the estimate from Equation 5, and square both sides to get:

$$\frac{(x_{test}^T \hat{V}^{-1} y)^2}{x_{test}^T \hat{V}^{-1} x_{test}} \sim \chi_1^2. \quad (7)$$

1.1.3 Infinitesimal model

The infinitesimal model places a normal prior on the effect estimates β_{GRM} from Equation 2, and assumes the total genetic effect explained by all the genetic markers to be σ_g^2:

$$\beta_{GRM} \sim \mathcal{N}(0, \sigma_g^2 I_M / M). \quad (8)$$

As the prior and likelihood are conjugate, we can infer the posterior distribution $P(\beta_{GRM} \mid X_{GRM}, y)$:

$$P(\beta_{GRM} \mid X_{GRM}, y) = \mathcal{N}\left(\left(\frac{I_M M}{\sigma_g^2} + \frac{X_{GRM}^T X_{GRM}}{\sigma_e^2}\right)^{-1} \frac{X_{GRM}^T y}{\sigma_e^2}, \left(\frac{I_M M}{\sigma_g^2} + \frac{X_{GRM}^T X_{GRM}}{\sigma_e^2}\right)^{-1}\right) \quad (9)$$

The maximum-a-posteriori (MAP) estimate of β_{GRM} in Equation 9, also referred to as the best linear unbiased predictor (BLUP), can be written as:

$$\hat{\beta}_{GRM} = \left(\frac{I_M M}{\sigma_g^2} + \frac{X_{GRM}^T X_{GRM}}{\sigma_e^2}\right)^{-1} \frac{X_{GRM}^T y}{\sigma_e^2} \quad (10)$$

The residual phenotype, formed by regressing out the effect of other variants on the phenotype, is given by $\tilde{y} = y - X_{GRM}\hat{\beta}_{GRM}$ and simplifies to

$$\tilde{y} = y - X_{GRM}\hat{\beta}_{GRM} = \sigma_e^2 V^{-1} y \quad (11)$$

This provides a link between the BLUP and test statistics for x_{test} in Equation 7, which is exploited to compute test statistics for non-infinitesimal models. Under the infinitesimal model, the test statistic can be rewritten as

$$\frac{(x_{test}^T \tilde{y} / \sigma_e^2)^2}{x_{test}^T \hat{V}^{-1} x_{test}} \sim \chi_1^2 \quad (12)$$

1.1.4 Non-infinitesimal model

It is often the case that a large fraction of variants are not linked to the phenotype, so the above model can be improved by adopting a more appropriate prior, such as a spike-and-slab prior, or a mixture-of-Gaussians prior. The spike-and-slab prior can be modelled as follows:

$$P(\beta_{GRM}) \sim p_0\mathcal{N}(0, \sigma_g^2/Mp_0) + (1-p_0)\delta(0), \tag{13}$$

where $\delta(0)$ is the Dirac-delta function at 0. A mixture-of-Gaussians prior replaces the Dirac-delta function using a low-variance normally distributed component, and can be written as

$$P(\beta_{GRM}) \sim p_0\mathcal{N}(0, \sigma_{g,1}^2/M) + (1-p_0)\mathcal{N}(0, \sigma_{g,2}^2/M). \tag{14}$$

Equation 12 can still be used to compute test statistics, by replacing $\tilde{y}$ with the phenotype residuals calculated using BLUP under the non-infinitesimal model. Note that it is often not possible to obtain a closed-form solution for the BLUP under an non-infinitesimal model. BOLT-LMM [3] therefore uses a variational inference approach to estimate the genetic effects.

1.2 Bayesian inference

Bayesian inference is a popular statistical inference approach that takes into account prior beliefs about the model along with evidence obtained from the data. We introduce some basic terminology related to Bayesian inference, which we utilize in the following sections to describe our inference approach. We refer the reader to [9, 10] for a more in-depth review on Bayesian inference and variational Bayes approaches. We use the following notation and terminology:

- $X = (x_1, x_2, ..., x_n)$ is a set of n observed data-points
- θ parameterizes the probability distribution of the data-points, $x \sim P(x \mid \theta)$.
- The **prior** distribution, $P(\theta)$, is the distribution of the parameters before any data is observed.
- The **data likelihood** or likelihood, $P(X \mid \theta)$, is the distribution of the data conditioned on model parameters.
- The **evidence** or marginal likelihood is the distribution of the observed data marginalized over parameters,

$$P(X) = \int P(X \mid \theta)P(\theta)d\theta.$$

- The **posterior**, $P(\theta \mid X)$, is the distribution of the parameters after taking into account the observed data,

$$P(\theta \mid X) = \frac{P(X \mid \theta)P(\theta)}{P(X)} = \frac{P(X \mid \theta)P(\theta)}{\int P(X \mid \theta)P(\theta)d\theta}.$$

In the context of Bayesian inference, we are most interested in deriving the posterior distribution given prior beliefs on the parameters. Unless the likelihood and prior are conjugate, however, we generally cannot derive a closed-form solution for the posterior. Thus, in more general and usually more complex scenarios, Bayesian Inference heavily relies on sampling approaches, such as MCMC, or approximate methods, such as variational Bayes, to infer the posterior.

1.3 Variational inference

Variational inference is a popular approximate inference technique often used to infer posterior estimates for models that do not allow for a closed-form solution for their posterior. In variational inference, posterior inference is formulated as an optimization problem. As the exact posterior probability is often intractable, we assume an approximate posterior family that is easy to evaluate. We then optimize the parameters of this posterior family so that the Kullback–Leibler (KL) divergence between the approximate and true posteriors is sufficiently small:

$$KL\left(q_\omega(\theta) \| P(\theta \mid X)\right) = \int q_\omega(\theta) \log\left(\frac{q_\omega(\theta)}{P(\theta \mid X)}\right) d\theta, \tag{15}$$

where $P(\theta \mid X)$ is the true posterior and $q_\omega(\theta)$ is the approximate posterior, parameterized by ω (also known as variational parameters). Variational inference aims to minimize the KL divergence in Equation 15, to obtain optimal variational parameters ω^*:

$$\omega^* = \underset{\omega}{\text{argmin}} KL(q_\omega(\theta)||P(\theta|\mathbf{X})). \tag{16}$$

1.3.1 Evidence lower bound

Note that Equation 16 poses an optimization problem that requires the calculation of KL divergence between approximate posterior and the true posterior. However, this objective cannot be computed because it requires information about the posterior, which is not available in closed form. We thus use the Bayes rule to simplify the above equation,

$$\begin{aligned} KL(q_\omega(\theta)||P(\theta|\mathbf{X})) &= \mathbb{E}[log(q_\omega(\theta))] - \mathbb{E}[log(P(\theta|\mathbf{X})] \\ &= \mathbb{E}[log(q_\omega(\theta))] - \mathbb{E}[log(P(\theta, \mathbf{X})] + log(P(\mathbf{X})) \\ &= KL(q_\omega(\theta)||P(\theta)) - \mathbb{E}[log(P(\mathbf{X}|\theta)] + log(P(\mathbf{X})) \geq 0 \end{aligned} \tag{17}$$

$$\implies log(P(\mathbf{X})) \geq \mathbb{E}[log(P(\mathbf{X}|\theta)] - KL(q_\omega(\theta)||P(\theta)). \tag{18}$$

In the above, the term on the left is the logarithm of the evidence, so the term on the right is called the evidence lower bound, or ELBO. Variational inference algorithms try to minimize the KL divergence between the true posterior and approximate posterior by maximizing the ELBO in Equation 18. The ELBO can also be seen as the difference between the expected log-likelihood of the data and the KL divergence between the approximate posterior and prior,

$$\mathcal{L}_{VI}(\omega) = \mathbb{E}[log(P(\mathbf{X}|\theta)] - KL(q_\omega(\theta)||P(\theta)). \tag{19}$$

In the context of Bayesian regression, our sampling distribution is $P(y \mid X)$ instead of $P(X)$, where y is the outcome variable and X is the input variable. In that case, the ELBO is written as

$$\mathcal{L}_{VI}(\omega) = \underbrace{\mathbb{E}[log(P(\mathbf{y}|\theta, \mathbf{X})]}_{\text{Expected LL}} - \underbrace{KL(q_\omega(\theta)||P(\theta))}_{\text{KL-divergence}}. \tag{20}$$

1.3.2 Mean-field variational inference

The fully-factorized or mean-field posterior is a commonly used approximate posterior in variational inference. In this approach, the posterior can be represented as a product of factors with only one parameter per factor. This enables the use of coordinate-ascent variational inference algorithms and makes operations such as sampling and computation of the ELBO, used in other variational inference strategies, more efficient.

Using this approach, the model we adopt when estimating genetic effects in quantitative traits, i.e., a Bayesian linear regression with spike-and-slab prior and a fully-factorized spike-and-slab approximate posterior, takes the form

$$\begin{aligned} &\textbf{Likelihood:} && P(y \mid X, \beta) \sim \mathcal{N}(\beta^T X, \sigma_e^2), \\ &\textbf{Prior:} && P(\beta) = \prod_j P(\beta_j),\ P(\beta_j) \sim (1-p_0)\mathcal{N}(0, \sigma^2) + p_0\delta(0), \\ &\textbf{Approximate Posterior:} && q(\beta) = \prod_j q(\beta_j),\ q(\beta_j) \sim (1-\psi_j)\mathcal{N}(\mu_j, \sigma_j^2) + \psi_j\delta(0). \end{aligned} \tag{21}$$

In the above, y and X are the output and input variables, respectively, β are the unknown effects of the linear regression, which have a spike-and-slab prior $P(\beta)$. The approximate posterior in this model assumes a fully-factorized spike-and-slab structure with variational parameters μ, ψ, and σ. As shown in [11], a spike-and-slab prior is conjugate to the normal likelihood, making a fully-factorized spike-and-slab posterior a good choice for the approximate posterior family.

1.3.3 Coordinate ascent variational inference

Coordinate ascent variational inference (CAVI), introduced in [10], is one of the most popular mean-field variational inference algorithms. CAVI iteratively optimizes each factor in the mean-field posterior while fixing the other factors, thereby improving the ELBO in each iteration and finding a local optimum. CAVI updates each factor in the approximate posterior as follows:

$$\log q({\beta^*}_j) = E_{\beta_{(-j)}}[\log(P(y,\beta))] + C \tag{22}$$

The optimal update for a variational parameter is proportional to the exponential of the expected logarithm of the complete conditional, where the expectation is taken with respect to all the variational parameters except the one which is being updated. It can be shown that the above updates increase the ELBO each iteration (see [9] for a concise proof). We next derive the CAVI update equations for the Bayesian linear regression model described in Equation 21.

Derivation of CAVI updates for spike-and-slab Bayesian linear regression:

$$\begin{aligned}\log q({\beta^*}_j) &= E_{\beta_{(-j)}}[\log(P(y,\beta))] + C\\ &= E_{\beta_{(-j)}} \log P(y \mid \beta) + E_{\beta_{(-j)}} \log P(\beta_j) + C\\ &= \sum_{n=1}^{N} \left(E_{\beta_{(-j)}} \log P(y_n \mid \beta)\right) + E_{\beta_{(-j)}} \log P(\beta_j) + C.\end{aligned} \tag{23}$$

To show this, we will ignore the terms in the complete conditional that do not depend on β_j and simplify the complete conditional for N data points. We will also remove terms which do not depend on β_j and absorb them in the constant C; the constant is thus not necessarily the same from one step to the other, but we simplify the notation by always expressing it as C.

We start by simplifying the equation by substituting the normal log-likelihood and spike-and-slab prior,

$$\log q({\beta^*}_j) = \sum_{n=1}^{N} E_{\beta_{(-j)}}\Big(-\frac{(y_n-\beta^T x_n)^2}{2\sigma_e^2} - log(\sigma_e)\Big) + E_{\beta_{(-j)}} \log P(\beta_j) + C. \tag{24}$$

Because the spike-and-slab distribution can be seen as mixture of non-overlapping Gaussians, [11] shows that it forms an exponential family (see theorems 1 and 2 in [11]). The natural parameters for the spike-and-slab distribution $(1-p_0)\mathcal{N}(\mu,\sigma^2) + p_0\delta(0)$ are $-\frac{1}{2\sigma^2}$, $\frac{\mu}{\sigma^2}$, and $\log p_0 - \log(1-p_0) + \frac{\mu^2}{2\sigma^2} + \frac{1}{2}\log\sigma^2$, with corresponding sufficient statistics $\mathbb{I}\{\beta_j \neq 0\}\beta_j^2$, $\mathbb{I}\{\beta_j \neq 0\}\beta_j$, and $\mathbb{I}\{\beta_j = 0\}$. We can substitute the natural parameters for the prior to simplify further:

$$\begin{aligned}&\log q({\beta^*}_j) =\\ &= \sum_{n=1}^{N} E_{\beta_{(-j)}}\left(-\frac{(y_n-\beta^T x_n)^2}{2\sigma_e^2}\right) + E_{\beta_{(-j)}}\left(-\mathbb{I}\{\beta_j \neq 0\}\frac{\beta_j^2}{2\sigma^2} - \mathbb{I}\{\beta_j = 0\}(\log p_0 - \log(1-p_0) + log(\sigma))\right) + C\\ &= -E_{\beta_{(-j)}}\left(\frac{\sum_{n=1}^{N}(y_n - \beta_j x_{n,j} - \sum_{i\neq j}\beta_i x_{n,i})^2}{2\sigma_e^2}\right) - \mathbb{I}\{\beta_j \neq 0\}\frac{\beta_j^2}{2\sigma^2} - \mathbb{I}\{\beta_j = 0\}\left(\log\frac{p_0\sigma}{1-p_0}\right) + C\\ &= -E_{\beta_{(-j)}}\left(\frac{\sum_{n=1}^{N}(\beta_j x_{n,j} - (y_n - \sum_{i\neq j}\beta_i x_{n,i}))^2}{2\sigma_e^2} + \mathbb{I}\{\beta_j \neq 0\}\frac{\beta_j^2}{2\sigma^2}\right) - \mathbb{I}\{\beta_j = 0\}\left(\log\frac{p_0\sigma}{1-p_0}\right) + C\\ &= -E_{\beta_{(-j)}}\left(\frac{\sum_{n=1}^{N}(\beta_j^2 x_{n,j}^2 - 2(y_n - \sum_{i\neq j}\beta_i x_{n,i})\beta_j x_{n,j})}{2\sigma_e^2} + \frac{\beta_j^2}{2\sigma^2}\right)\mathbb{I}\{\beta_j \neq 0\} - \left(\log\frac{p_0\sigma}{1-p_0}\right)\mathbb{I}\{\beta_j = 0\} + C\\ &= -\left(\frac{\sum_{n=1}^{N} x_{n,j}^2}{2\sigma_e^2} + \frac{1}{2\sigma^2}\right)\mathbb{I}\{\beta_j \neq 0\}\beta_j^2 + E_{\beta_{(-j)}}\left(\frac{\sum_{n=1}^{N}(y_n - \sum_{i\neq j}\beta_i x_{n,i})x_{n,j})}{\sigma_e^2}\right)\mathbb{I}\{\beta_j \neq 0\}\beta_j +\\ &\quad - \left(\log\frac{p_0\sigma}{1-p_0}\right)\mathbb{I}\{\beta_j = 0\} + C.\end{aligned} \tag{25}$$

Substituting the expected value of the approximate spike-and-slab posterior $(1-\psi_i)\mathcal{N}(\mu_i,\sigma_i)+\psi_i\delta(0)$ with $(1-\psi_i)\mu_i$,

$$\begin{aligned}\log q({\beta^*}_j) = &-\left(\frac{\sum_{n=1}^{N} x_{n,j}^2}{2\sigma_e^2}+\frac{1}{2\sigma^2}\right)\mathbb{I}\{\beta_j \neq 0\}\,\beta_j^2 + \\ &+\left(\frac{\sum_{n=1}^{N}(y_n-\sum_{i\neq j}\mu_i(1-\psi_i)x_{n,i})x_{n,j})}{\sigma_e^2}\right)\mathbb{I}\{\beta_j \neq 0\}\,\beta_j + \\ &-\left(\log\frac{p_0\sigma}{1-p_0}\right)\mathbb{I}\{\beta_j = 0\}+C. \end{aligned} \tag{26}$$

Now comparing the coefficients of the sufficient statistics $\mathbb{I}\{\beta_j\neq 0\}\,\beta_j^2$, $\mathbb{I}\{\beta_j\neq 0\}\,\beta_j$ and $\mathbb{I}\{\beta_j=0\}$ to the natural parameters of the spike-and-slab $-\frac{1}{2\sigma_j^2}$, $\frac{\mu_j}{\sigma_j^2}$ and $\log\psi_j-\log(1-\psi_j)+\frac{\mu_j^2}{2\sigma_j^2}+\frac{1}{2}\log\sigma_j^2$, respectively [11], we get the following update rules:

$$\begin{aligned}\sigma_j^2 &= \frac{1}{\frac{\sum_{n=1}^{N} x_{n,j}^2}{\sigma_e^2}+\frac{1}{\sigma^2}} \\ \mu_j &= \frac{\sum_{n=1}^{N}(y_n-\sum_{i\neq j}\mu_i(1-\psi_i)x_{n,i})x_{n,j})}{\sum_{n=1}^{N}x_{n,j}^2+\frac{\sigma_e^2}{\sigma^2}} \\ \psi_j &= 1-\frac{1}{1+\frac{p_0}{1-p_0}\sqrt{1+\sigma^2/\sigma_e^2\sum_{n=1}^{N}x_{n,j}^2}\,\exp\left\{-\frac{\left(\sum_{n=1}^{N}\left(y_n-\sum_{i\neq j}\mu_i(1-\psi_i)x_{n,i})x_{n,j}\right)\right)^2}{2\sigma_e^4/\sigma^2+2\sigma_e^2\sum_{n=1}^{N}x_{n,j}^2}\right\}}\end{aligned} \tag{27}$$

The updates for the variational parameter σ_j^2 (variance of the Gaussian in the spike-and-slab posterior) do not depend on y; we use these updates to initialize our variational parameters σ_j^2 and μ_j.

1.3.4 Stochastic variational inference

CAVI provides a principled way to perform variational inference but requires a full pass through the data for each iteration, which is computationally expensive for large datasets. BOLT-LMM [3] [4] observed that CAVI scales approximately as $\mathcal{O}(MN^{1.5})$, because the number of CAVI steps increase by $\sqrt{N}$, where M is the number of markers and N is the number of samples. Stochastic variational inference [12] uses ideas from stochastic optimization [13] to update the variational parameters through a noisy but unbiased estimate of ELBO's gradient. Like other modern machine learning optimizers, stochastic variational inference is also amenable to working with batches of data, rather than with a full pass through the data, making it more efficient when applied to large datasets. Overall, stochastic variational inference provides a more efficient and scalable approach for posterior inference and has been widely applied to many machine learning problems, ranging from topic modeling [12] to generative models such as variational autoencoders (VAE) [14].

We now derive the stochastic variational inference objective for the Bayesian linear regression model described in Equation 21 for quantitative traits and a similar Bayesian logistic regression model for binary traits. In Section 2.2.2, we highlight several practical considerations linked to implementing the algorithm.

Derivation of Stochastic VI objective for spike-and-slab Bayesian linear regression:

Stochastic VI tries to find the optimal variational parameters by performing gradient descent directly on the ELBO objective in Equation 20,

$$L_{VI}(\psi,\mu,\sigma) = E[log(P(y\mid\beta,X)]-KL(q_{\psi,\mu,\sigma}(\beta)\mid P(\beta)) \tag{28}$$

Assuming the same model as in Equation 21 with a fully factorized spike and slab prior, the ELBO can be simplified as follows:

$$
\begin{aligned}
L_{VI}^{Q}(\psi,\mu,\sigma) &= \sum_{n=1}^{N} E[log(p(y_n \mid \beta))] - KL(q_{\psi,\mu,\sigma}(\beta) \mid P(\beta)) \\
&= \sum_{n=1}^{N} E[log(p(y_n \mid \beta))] - \sum_{j=1}^{M} KL(q_{\psi_j,\mu_j,\sigma_j}(\beta_j) \mid P(\beta_j)) \\
&= -\sum_{n=1}^{N} \int \frac{(y_n - \beta^T x_n)^2}{2\sigma_e^2} q_{\psi,\mu,\sigma}(\beta) d\beta - \sum_{j=1}^{M} KL(q_{\psi_j,\mu_j,\sigma_j}(\beta_j) \mid P(\beta_j)),
\end{aligned} \tag{29}
$$

where N is the number of samples, M is the number of markers, and β are the markers' effect estimates. We derive the KL divergence between two spike-and-slab distributions as a special case of the KL divergence between non-overlapping mixture distributions (see Appendix for details). We do not have a closed form for the log-likelihood term. The usual approach in stochastic variational inference is to replace the integral with an approximate monte-carlo estimate. This results in a noisy yet unbiased estimate of the ELBO, which can be shown to converge to a local minimum under the Robbins-Munro condition [13] on step-size:

$$
\begin{aligned}
L_{VI}^{Q}(\psi,\mu,\sigma) \approx &-\sum_{n=1}^{N}\sum_{s=1}^{S} \frac{(y_n - \beta(s)x_n)^2}{2\sigma_e^2} + \\
&-\sum_{j=1}^{M} \left(\frac{\psi_j}{2} \left(-1 + \frac{\mu_j^2 + \sigma_j^2}{\sigma^2} - \log \frac{\sigma_j^2}{\sigma^2} \right) + (1-\psi_j) \log \frac{1-\psi_j}{1-p_0} + \psi_j \log \frac{\psi_j}{p_0} \right).
\end{aligned} \tag{30}
$$

We take S monte-carlo samples from the approximate posterior $\beta(s)$ to calculate the approximate log-likelihood. Similar to the use of stochastic optimization in machine learning, we can also have a batched version of the above ELBO, where we update the variational parameters after each mini-batch of data. We divide the input data into B equally sized batches and maximize the batched-version of ELBO given below:

$$
\begin{aligned}
L_{VI}^{Q}(\psi,\mu,\sigma) \approx &-\sum_{b=1}^{B} \left(\sum_{s=1}^{S} \frac{(y_b - \beta(s)X_b)^2}{2\sigma_e^2} + \right. \\
&\left. + \frac{1}{B} \sum_{j=1}^{M} \left(\frac{\psi_j}{2} \left(-1 + \frac{\mu_j^2 + \sigma_j^2}{\sigma^2} - \log \frac{\sigma_j^2}{\sigma^2} \right) + (1-\psi_j) \log \frac{1-\psi_j}{1-p_0} + \psi_j \log \frac{\psi_j}{p_0} \right) \right).
\end{aligned} \tag{31}
$$

In practice, we make use of a local reparameterization trick [15] and antithetic variates to sample the approximate predictions $\beta(s)X_b$ while reducing the variance in the ELBO gradient estimates. We provide details on sampling and other practical considerations in Section 2.2.2.

Derivation of Stochastic VI objective for spike-and-slab Bayesian logistic regression:

The Bayesian logistic regression model is similar to the linear regression case, only differing in the likelihood term. The model for Bayesian logistic regression with spike-and-slab prior can be written as

$$
\begin{aligned}
&\textbf{Likelihood:} && P(y \mid X,\beta) = \prod_{n=1}^{N} c_n^{y_n} \{1-c_n\}^{1-y_n},\ c_n = \sigma(\beta^T X_n), \\
&\textbf{Prior:} && P(\beta) = \prod_j P(\beta_j),\ P(\beta_j) \sim (1-p_0)\mathcal{N}(0,\sigma^2) + p_0\delta(0), \\
&\textbf{Approximate Posterior:} && q(\beta) = \prod_j q(\beta_j),\ q(\beta_j) \sim (1-\psi_j)\mathcal{N}(\mu_j,\sigma_j^2) + \psi_j\delta(0),
\end{aligned} \tag{32}
$$

where X_n and y_n represent the genotype vector and phenotype values for the n-th individual. We still assume a spike-and-slab approximate posterior, although it is not conjugate to the logistic regression model, because we

empirically find it to still perform well in this case. The ELBO for Bayesian logistic regression can be simplified as in the linear case:

$$\begin{aligned} L_{VI}^{Bi}(\psi,\mu,\sigma) &= \sum_{n=1}^{N} E[log(p(y_n \mid \beta))] - KL(q_{\psi,\mu,\sigma}(\beta) \mid P(\beta)) \\ &= -\sum_{n=1}^{N} \int \{y_n \log c_n + (1-y_n)\log(1-c_n)\} q_{\psi,\mu,\sigma}(\beta) d\beta - \sum_{j=1}^{M} KL(q_{\psi_j,\mu_j,\sigma_j}(\beta_j) \mid P(\beta_j)), \end{aligned} \tag{33}$$

where $c_n = \sigma(\beta^T X_n)$, i.e., the output after sigmoid activation. We estimate the expected log-likelihood with a Monte Carlo estimate, whereas we write a closed-form solution for the KL divergence term similar to Equation 31. The batched version of the ELBO for Bayesian logistic regression can be written as

$$\begin{aligned} L_{VI}^{Bi}(\psi,\mu,\sigma) \approx -\sum_{b=1}^{B} \Bigg(&\sum_{s=1}^{S} y_b \log(\sigma(\beta^T X_b)) + (1-y_b)\log(1-\sigma(\beta^T X_b)) + \\ &+ \frac{1}{B}\sum_{j=1}^{M} \left(\frac{\psi_j}{2}\left(-1 + \frac{\mu_j^2+\sigma_j^2}{\sigma^2} - \log\frac{\sigma_j^2}{\sigma^2}\right) + (1-\psi_j)\log\frac{1-\psi_j}{1-p_0} + \psi_j \log\frac{\psi_j}{p_0} \right) \Bigg), \end{aligned} \tag{34}$$

where we take S Monte Carlo samples to approximate the log-likelihood term. We also use the same approaches used in the Bayesian linear regression to reduce the variance in the ELBO gradient estimates.

2 Quickdraws: Algorithm details

2.1 Preprocessing

Our analyses make use of the following filtering criteria:

1. **Genotype QC:** for model fitting, we use markers with minor allele frequency $\geq 1\%$, Hardy-Weinberg equilibrium test $P > 1 \times 10^{-15}$, and genotyping rate $> 99\%$.
2. **Missing data:** we remove samples with phenotype missingness above 50% and mean-impute the remaining missing values. During the Bayesian regression step, we also median-impute the genotype matrix to enable 2-bit genotype encoding. We allow missing data during the computation of test statistics.
3. **Mean-centering and transforming:** all analyses are performed on mean-centered and standardized genotypes. Quantitative phenotypes are mean centered, and quantile normalized to a normal distribution.
4. **Covariates:** we regress covariates (top 20 PCs, age, sex, age^2, age$\times$sex, age$^2\times$sex, smoking status) from both genotype and phenotype on the fly, which is equivalent to including covariates as fixed effects in the model. We also remove individuals with any missing covariate ('complete case analysis').

2.2 Bayesian regression

The Bayesian regression step has a crucial role in increasing association power. We use stochastic variational inference with a spike-and-slab prior on the effect estimates to perform the regression, using leave-one-chromosome-out.

2.2.1 Optimizing the stochastic VI objective

We provide a derivation of the stochastic variational inference (VI) objective used to minimize the KL divergence between the approximate posterior and true posterior in Section 1.3.4. As previously described, we use stochastic optimization strategies [13] to update the evidence lower-bound (ELBO), using mini-batches of data. This approach provides a scalable and efficient alternative to coordinate-ascent variational inference. The VI objective to be maximized is given as L^{Q}_{VI} for quantitative traits and L^{Bi}_{VI} for binary traits:

$$\begin{aligned} L^{Q}_{VI}(\psi,\mu,\sigma) \approx -\sum_{b=1}^{B}\Bigg(&\sum_{s=1}^{S}\frac{(y_b-\beta(s)X_b)^2}{2\sigma_e^2}+ \\ &+\frac{1}{B}\sum_{j=1}^{M}\left(\frac{\psi_j}{2}\left(-1+\frac{\mu_j^2+\sigma_j^2}{\sigma^2}-\log\frac{\sigma_j^2}{\sigma^2}\right)+(1-\psi_j)\log\frac{1-\psi_j}{1-p_0}+\psi_j\log\frac{\psi_j}{p_0}\right)\Bigg) \end{aligned} \tag{35}$$

$$\begin{aligned} L^{Bi}_{VI}(\psi,\mu,\sigma) \approx -\sum_{b=1}^{B}\Bigg(&\sum_{s=1}^{S}y_b\log(\sigma(\beta^T X_b))+(1-y_b)\log(1-\sigma(\beta^T X_b))+ \\ &+\frac{1}{B}\sum_{j=1}^{M}\left(\frac{\psi_j}{2}\left(-1+\frac{\mu_j^2+\sigma_j^2}{\sigma^2}-\log\frac{\sigma_j^2}{\sigma^2}\right)+(1-\psi_j)\log\frac{1-\psi_j}{1-p_0}+\psi_j\log\frac{\psi_j}{p_0}\right)\Bigg) \end{aligned} \tag{36}$$

Because stochastic variational inference is amenable to batch-wise optimization, the objective function is written as a sum over batches. In particular, we use a 8-bit Adam optimizer [16] to optimize the variational parameters in the stochastic VI objective. The low-bit Adam quantizes the gradient information (which is stored for each parameter) to an 8-bit representation, instead of the default 32-bit, thereby saving GPU memory and allowing to work with multiple models in parallel. We did not observe this gradient quantization to cause significant differences in performance.

2.2.2 ELBO variance reduction

Stochastic VI methods provide a principled and efficient way to perform variational inference. This approach is simple but requires particular care to make sure that the variance of the ELBO estimate is not too large, as that may have a negative impact on convergence. We make use of multiple recently developed strategies to reduce variance

in the stochastic estimation of the ELBO, including continuous relaxation [17, 18], local reparameterization trick [15], and antithetic variates [19].

Reparameterization: When dealing with stochastic objectives, such as the ELBO, it is often difficult to differentiate the objective due to intrinsic randomness in the parameters. One solution to this problem is provided by the reparameterization trick [14, 20], which models the stochasticity in the parameters as an input to the model rather than an intrinsic property of the model. In more detail, the reparameterization trick assumes that distributions can be reparameterized in the form $g(\theta, \epsilon)$ where θ is the variational parameter, ϵ is the independently sampled random variable, and g is a differentiable function. As an example, consider the case of an isotropic normal distribution. To sample from a mean-field normal distribution, we may reparameterize the sample as follows:

$$\begin{aligned} X \sim \mathcal{N}(\mu, \sigma^2 I) &\implies X = \mu + \epsilon \cdot \sigma, \\ \epsilon \sim \mathcal{N}(0, I), & \end{aligned} \tag{37}$$

where ϵ is i.i.d. normal noise with no dependence on μ and σ. Relying on the reparameterization and treating ϵ as an independent input, we can differentiate with respect to μ and σ.

Local reparamaterization: The local reparamaterization trick [15] was introduced as a way to provide computationally fast and low-variance gradient estimators in parameter-heavy stochastic VI methods. It was originally applied to perform variational inference in Bayesian neural networks but is easily extended to Bayesian regression. Using the original reparamaterization trick [14, 20] leads to sampling i.i.d. noise at least once for each parameter, making this approach computationally intensive in high-dimensional settings, which may involve millions of parameters. The local reparamaterization trick translates the uncertainty on individual effect estimates, β, into local noise in the output, $\beta^T X$, thus reducing the computational overhead. In the simple mean-field Gaussian VI, the local reparameterization trick can be used to sample the outputs directly,

$$w_{i,j} \sim \mathcal{N}(\mu_{i,j}, \sigma_{i,j}^2), \quad b_{m,j} = \sum_i a_{m,i} w_{i,j}, \tag{38}$$

where $a_{m,i}$ is the m-th input data point and $b_{m,j}$ is the output corresponding to the m-th data point in the Bayesian linear regression model. $b_{m,j}$ is a weighted sum of normally distributed random variables, implying that $b_{m,j}$ is also normally distributed, with the following parameters:

$$\begin{aligned} w_{i,j} \sim \mathcal{N}(\mu_{i,j}, \sigma_{i,j}^2) &\implies b_{m,j} \sim \mathcal{N}(\gamma_{m,j}, \delta_{m,j}^2), \\ \gamma_{m,j} = \sum_i a_{m,i}\mu_{i,j}, &\quad \delta_{m,j}^2 = \sum_i a_{m,i}^2 \sigma_{i,j}^2. \end{aligned} \tag{39}$$

In order to sample this output directly, one can apply the original reparameterization trick for the normal distribution:

$$b_{m,j} = \gamma_{m,j} + \epsilon_{m,j}\delta_{m,j}, \quad \epsilon_{m,j} \sim \mathcal{N}(0, 1). \tag{40}$$

Note that in Bayesian linear/logistic regression the local reparameterization removes the need to sample all the weights in a layer and only requires sampling proportional to the number of output variables for each mini-batch of data, substantially improving the computational complexity of the algorithm.

The local reparamaterization trick was originally only derived for mean-field Gaussian variational inference, since the weighted sum of arbitrary distributions cannot always be analytically derived. In the case of mean-field spike-and-slab distributions, we use the central limit theorem to approximate the weighted sum of independent spike-and-slab distributions as approximately Gaussian. Note that the input dimension for Bayesian regression in our setup is quite high (usually $> 100,000$ markers), justifying this approximation. This idea was first explored in [21] to sample discrete weights for binary and ternary neural networks. We thus reparametrize the output of our model (as described in equations 21 and 32) as follows:

$$\begin{aligned} w_{i,j} \sim p_{i,j}\mathcal{N}(\mu_{i,j}, \sigma_{i,j}^2) + (1 - p_{i,j})\delta(0) &\implies b_{m,j} \sim \mathcal{N}(\gamma_{m,j}, \delta_{m,j}), \\ \gamma_{m,j} = \sum_i a_{m,i}\mu_{i,j}p_{i,j}, &\quad \delta_{m,j} = \sum_i a_{m,i}^2 (p_{i,j}\sigma_{i,j}^2 + p_{i,j}\mu_{i,j}^2 - p_{i,j}^2\mu_{i,j}^2). \end{aligned} \tag{41}$$

Antithetic variates for variance reduction: The use of antithetic variates is a popular approach to speed up Monte Carlo computations of the expectation of a random function [19]. This strategy relies on taking the antithetic path of the sampled path to reduce the variance in the overall estimator. For example, in order to sample from a normal random variable (with mean μ and variance σ^2), one might use the reparameterization trick to sample $\mu + \epsilon\sigma$. The antithetic path for a normally distributed random variable is $\mu - \epsilon\sigma$. In addition to providing a valid sample from the underlying distribution (in this case, normal with mean μ and variance σ^2), the antithetic path also reduces the variance of the Monte Carlo estimate, as the sampled path and antithetic path are often negatively correlated.

We use both antithetic variates and the local reparameterization trick to reduce the variance of the ELBO. As described above, the antithetic path for a normally distributed random variable is simply obtained by replacing ϵ with $-\epsilon$. For each forward pass, we thus obtain two Monte Carlo samples for the output of the model as follows:

$$\begin{aligned}
\beta(s_1) &= (\sum_i a_{m,i}\mu_{i,j} + \sqrt{\sum_i a_{m,i}^2(p_{i,j}\sigma_{i,j}^2 + p_{i,j}\mu_{i,j}^2 - p_{i,j}^2\mu_{i,j}^2}\epsilon), \\
\beta(s_2) &= (\sum_i a_{m,i}\mu_{i,j} - \sqrt{\sum_i a_{m,i}^2(p_{i,j}\sigma_{i,j}^2 + p_{i,j}\mu_{i,j}^2 - p_{i,j}^2\mu_{i,j}^2}\epsilon), \\
\beta(s) &= [\beta(s_1), \beta_2(s_2)].
\end{aligned} \tag{42}$$

2.2.3 Hyperparameter tuning

We perform cross-validation by randomly splitting the data into training (80%) and testing (20%) sets to estimate the optimal sparsity hyperparameter $1 - p_0$ (see Equation 21) using the values $\{0.5, 0.2, 0.1, 0.05, 0.02, 0.01\}$. For heritability in binary traits, we evaluate $\{0.01, 0.25, 0.5, 0.75\}$. Compared to coordinate-ascent variational inference, stochastic variational inference relies on additional hyperparameters that affect the final testing performance. These include the learning rate of the optimizer, the batch-size, and the number of training epochs. Setting a high learning rate results in unstable convergence of the optimizer, while a low learning rate leads to the need for more training epochs. Larger batch sizes lead to better performance but increased GPU memory usage. Considering these trade-offs, we set the batch-size to 128; the learning rate corresponding to each value of the sparsity hyperparameter was set to 4×10^{-4}, 2×10^{-4}, 2×10^{-4}, 1×10^{-4}, 2×10^{-5}, 5×10^{-6} for sparsity $(1-p_0) \in \{0.01, 0.02, 0.05, 0.1, 0.2, 0.5\}$. We also set the number of training epochs to 80 for the initial cross-validation step, and to 40 for the final LOCO Bayesian linear regression. We observed these hyperparameters to be robust across various sample sizes, providing a good balance between statistical power and computational costs.

2.3 Calculation of test statistics

Quantitative traits: After performing leave-one-chromosome-out Bayesian linear regression, we use the link between the BLUP or posterior mean effects and the test statistic described in Equation 12 to test for association in quantitative traits. The test statistic for a variant x_{test} is given by

$$\chi^2_{test} = \frac{(x_{test}^T\tilde{y}_{LOCO}/\sigma_e^2)^2}{x_{test}^T\hat{V}^{-1}x_{test}}, \tag{43}$$

where $\tilde{y}_{LOCO} = y - X_{GRM}\hat{\beta}_{LOCO}$ is the residual phenotype, estimated from the LOCO Bayesian linear regression, and σ_e^2 is the environmental variance, estimated using RHE-MC [7]. The denominator of 43 is further simplified by omitting $\hat{V}^{-1}$. [22] observed the ratio between $x_{test}^T\hat{V}^{-1}x_{test}$ and $x_{test}^T x_{test}$, referred to as GRAMMAR-Gamma ratio, to be nearly constant across variants. We can thus approximate the test statistic, up to a constant of proportionality, as

$$\chi^2_{test} \propto \frac{(x_{test}^T\tilde{y}_{LOCO})}{x_{test}^T x_{test}}. \tag{44}$$

We calculate the term on the right for all the tested variants and later calibrate the association statistics using the attenuation ratio estimated using LD-score regression [3, 23]. Note that we include covariates in the linear mixed model by regressing them out from both the genotype, x_{test}, and the residual phenotype, $\tilde{y}_{LOCO}$.

Binary traits: For binary traits, we model the association between genotype and phenotype through a logistic mixed model,

$$logit(p_i) = \alpha C + x_{test}\beta_{test} + g + \epsilon, \tag{45}$$

where $p_i = P(y_i = 1 \mid x_{test}, g, C)$ is the probability that the ith individual is a case given x_{test}, covariates C, and random genetic effects g. We test for association using a score-based test statistic. The score test is given by $T = x_{test}^T(y - \hat{p})$, where $\hat{p}$ is the estimated mean under the null model. The normalized test statistic is written as

$$T = \frac{x_{test}^T(y - \hat{p})}{\sqrt{x_{test}^T \hat{P} x_{test}}}, \tag{46}$$

where $P = \hat{V}^{-1} - \hat{V}^{-1}C(C^T\hat{V}^{-1}C)^{-1}C^T\hat{V}^{-1}$ is a dense $N \times N$ matrix, $\hat{V} = \frac{\hat{\sigma_g}^2}{M} X_{GRM}X_{GRM}^T + \hat{W}^{-1}$, and $\hat{W} = diag\{\hat{p}(1 - \hat{p})\}$. C and X_{GRM} represent the covariates and genotype matrix for model fitting. Similar to quantitative traits, the test statistics in Equation 46 can be simplified using a GRAMMAR-Gamma constant. We rewrite the test statistic in 46 up to a constant of proportionality as follows:

$$T_B \propto \frac{x_{test}^T(y - \hat{p})}{\sqrt{x_{test}^T \hat{W} x_{test}}}, \tag{47}$$

where $\hat{W}$ is a diagonal matrix with diagonal entries $\hat{p_i}(1 - \hat{p_i})$, obtained from the predictions of the null model in the Bayesian regression step.

Firth logistic regression: The test statistic from Equation 47 assumes that the null distribution of the test is normally distributed, which is often violated in traits with low prevalence or for rare variants. Firth logistic regression can be used to remove much of this bias, by adopting a Jeffrey's invariant prior.

The penalized likelihood for Firth logistic regression is given by

$$L(\alpha, \beta_{test}) = \sum_{i=1}^{N} y_i log p_i + (1 - y_i) log(1 - p_i) + \frac{\log |I(\alpha, \beta_{test})|}{2}, \tag{48}$$

where $I(\alpha, \beta_{test}) = U^T\hat{W}U$ with $U = (C; x_{test})$ is a Jeffrey's-prior penalty in the Firth logistic regression. In practice, performing Firth logistic regression requires computing matrix products and determinants of the form $U^T\hat{W}U$, which becomes computationally intensive as the number of covariates increases. The authors of Regenie [24] noticed that it is safe to assume that covariate effects are largely unchanged with or without the inclusion of a variant effect. To improve computational efficiency, they calculated the covariate effects under the null model once per chromosome and fixed it for Firth logistic regression to get variant effect estimates. The penalized likelihood under the approximate Firth logistic regression model is given by

$$L(\beta_{test}) = \sum_{i=1}^{N} y_i log p_i + (1 - y_i) log(1 - p_i) + \frac{\log |I^*(\beta_{test})|}{2}, \tag{49}$$

where $I^*(\beta_{test}) = x_{test}^T \hat{W} x_{test}$ uses the covariate-adjusted genotype vector. This reduces the computational overhead of Firth logistic regression and makes it independent on number of covariates (as the covariate effects are estimated once per chromosome, they require minimal time). We use the same strategy to perform Firth logistic regression, and then perform a likelihood ratio test to compute the test statistic and p-value,

$$T_{firth} = 2\{L(\beta_{test}) - L(0)\},\ T_{firth} \sim \chi_1^2. \tag{50}$$

Additionally, similarly to Regenie and FastGWA-GLMM, we perform approximate Firth logistic regression on the subset of variants for which the logistic regression p-value falls below a threshold, using a default cutoff of $p < 0.05$. We further narrow down the list of tested variants by only applying Firth logistic regression when the either the variant or the trait are rare (default: MAF $< 5\%$ or prevalence $< 5\%$).

Calibration of test statistics: We calibrate the summary statistics by estimating the effective sample size (ESS) increase compared to running linear regression on a homogeneous subset of unrelated individuals. To this end, we estimate the increase in ESS linked to the use of non-infinitesimal Bayesian linear regression (γ_{blr}), as well as the reduction in ESS due to the presence of close relatives (γ_{rel}). We then adjust the summary association statistics by matching the mean χ^2 statistic minus one [25] with that observed for linear or logistic regression run on

a homogeneous subset of unrelated individuals. In more detail, we multiply the uncalibrated Quickdraws summary statistics from equations 44 and 47 by a correction term c, computed as

$$c = \frac{\gamma_{rel}\gamma_{blr}\frac{N_{qd}}{N_{lr}}(< \chi^2_{lr} > -1) + 1}{< \chi^2_{qd} >}. \quad (51)$$

In the above, $< \chi^2_{qd} >$ and $< \chi^2_{lr} >$ are the mean χ^2 test statistics from Quickdraws and from linear/logistic regression run on a homogeneous unrelated subset of individuals, respectively. $\frac{N_{qd}}{N_{lr}}$ is the ratio of the total number of samples to the number of homogeneous unrelated samples used for linear/logistic regression. γ_{rel} and γ_{blr} are correction terms to account for relatedness and the use of Bayesian linear regression, which we describe in detail below. Note that the γ_{blr} term is estimated for each LOCO run, so the correction in equation 51 is performed separately for each chromosome. For binary traits, we use logistic regression and apply the correction separately for Firth-corrected and Firth-uncorrected variants.

Correcting the ESS for the presence of relatedness (γ_{rel} term). To estimate the effective number of samples in the presence of relatedness, we follow an approach similar to that of [26]. To this end, we first estimate genetic relatedness between samples using KING [27] as implemented in PLINK [28]. We only retain relationships up to the 3rd degree (KING score $> 2^{-\frac{9}{2}}$), as we found the ESS multiplier, described below, to not be significantly impacted by the inclusion of higher degree relatives. For each pair of relatives, we estimate the degree of the relationship using KING's output values as follows:

Range for KING ψ value	Relatedness
$\psi \geq 2^{-\frac{3}{2}}$	1 (Monozygotic Twins)
$2^{-\frac{3}{2}} > \psi \geq 2^{-\frac{5}{2}}$	0.5 (1st degree)
$2^{-\frac{5}{2}} > \psi \geq 2^{-\frac{7}{2}}$	0.25 (2nd degree)
$2^{-\frac{7}{2}} > \psi \geq 2^{-\frac{9}{2}}$	0.125 (3rd degree)

Genetic relationship estimates based on KING values.

Based on this, we compute the reduction factor for the effective sample size, γ_{rel}, using the following estimator, for which a derivation can be found in [26]:

$$\gamma_{rel} = \frac{tr((K\sigma_g^2 + I(1-\sigma_g^2))^{-1}K)}{N}. \quad (52)$$

In this expression, N is the total number of samples, K is the estimated kinship matrix, and σ_g^2 is the heritability of the trait. To efficiently compute γ_{rel}, we eigendecompose the matrix K, obtained using KING utilizing the block-like structure of the kinship matrix. In particular, the effective sample size multiplier in the presence of relatedness can be approximated as:

$$\gamma_{\text{rel}} \approx \frac{1}{N}\sum_{i=1}^{N}\frac{\lambda_i}{(\lambda_i - 1)\sigma_g^2 + 1}, \quad (53)$$

where λ_i is the ith eigenvalue of the kinship matrix K. And to facilitate this computation, we separately sum across each block B_i of related individuals:

$$\gamma_{\text{rel}} \approx \frac{1}{N}\sum_{i=1}^{B}\sum_{j=1}^{N_{B_i}}\frac{\lambda_{i,j}}{(\lambda_{i,j} - 1)\sigma_g^2 + 1}. \quad (54)$$

For binary traits, we convert the heritability from the liability scale to the observed scale.

Correcting the ESS for the use of Bayesian linear regression (γ_{blr} term): The effective sample size multiplier, γ_{blr}, has been shown to be proportional to the inverse of the residual trait variance in a linear regression model [3, 26]. To account for the increase in effective sample size in the model fitting step, we therefore calculate the inverse variance of the residual trait

$$\gamma_{blr} = \frac{Var(y)}{Var(y - \hat{y})} \quad (55)$$

where y and $\hat{y}$ are true and predicted trait values from the whole-genome regression regression on a held-out set. In more detail, we use the effect estimates from the best sparsity parameter to calculate the variance explained on held-out data. As the γ_{blr} term needs to be computed during the LOCO step, we compute these variance terms separately for each chromosome and sum across all chromosomes, excluding the one currently being tested.

3 Performance optimization

3.1 Memory optimization

1. **Raw genotypes:** for a diploid individual, the raw genotypes take values in $0, 1, 2$. We store the $N \times M$ genotype matrix in $NM/4$ bytes, i.e. using 2 bits per genotype entry. We do so by encoding 8 consecutive entries of the genotype matrix using two 8-bit integers using the built-in `numpy` features in Python, `np.packbits()` and `np.unpackbits()`. We also store the column-wise mean and variance of the genotype matrix, used to mean-center and standardize, on the fly.

2. **Genotype streaming during the calculation of test statistics:** the calculation of test statistics is usually performed on many more variants than those used for model fitting. We therefore optimize this step by streaming genotype blocks rather than loading all the variants into memory. The block size is determined based on the maximum memory allowed at runtime (default: 32GB) and the data IO is parallelized using multiple cores.

3.2 Speed optimization

1. **GPUs for fast Bayesian regression:** we use GPUs along with the `pytorch` machine learning package [29] to speed-up matrix multiplication and sampling operations during stochastic variational inference.

2. **Transfer learning:** rather than training the 22 LOCO models independently, we initialize each model training using the effect estimates previously computed for the whole-genome regression. This transfer learning approach requires fewer iterations to converge, making model fitting $2\times$ to $2.5\times$ faster, and leads to similar association power when compared to training each LOCO model separately. We compare the advantage of transfer learning over independent random initialization in simulations, by looking at the power improvement vs number of iterations in Supplementary Figure 18.

3. **Numba optimization for the calculation of test statistics:** we implement a fully-vectorized version of linear regression for multiple traits using the `parallel` and `njit` functionality from the Numba just-in-time (JIT) compiler [30].

4. **Calibrating test statistics using genotyped SNP data:** instead of running linear regression or logistic regression on all imputed variants, we calibrate our test statistics only using the genotype data provided in step 1 ($\sim$458k variants). We find that matching scaled effective sample size estimates using this subset of variants provides consistent calibration in the larger set of imputed variants, while greatly reducing running time, as we only need to evaluate the logistic or linear regression model on a subset of variants.

5. **HDF5 and PySnpTools for data loading:** we store the raw genotype matrix in a compressed HDF5 file [31], which provides fast sample-wise access (i.e., it allows obtaining all variants for an individual) for Bayesian regression. We instead use PySnpTools (see `https://github.com/fastlmm/PySnpTools`), which provides parallelizable variant-wise access, to access `bgen` or `bed` files for the calculation of test statistics.

6. **Approximate Firth logistic regression:** Approximate Firth regression is only applied to variants with logistic regression p-value below 0.05, as done in Regenie and FastGWA-GLMM. We further narrow down the list of variants by only applying Firth logistic regression to rare variants (MAF $< 5\%$) or rare traits (prevalence $< 5\%$).

7. **Multiple traits:** Most of our operations are vectorized using Numpy [32] and Numba [30], which leads to computational gains in the parallel analysis of multiple traits.

4 Summary of the Quickdraws algorithm

We provide a summary of the Quickdraws algorithm, which performs two main steps:

1. **Estimating genetic effects (model fitting):** This is further divided into two parts:
   (a) **Variance component estimation:** For quantitative traits, we use a multi-trait version of the RHE-mc algorithm [6–8] to obtain estimates of narrow-sense heritability. For binary traits, we directly perform the Bayesian regression while testing several heritability values in a grid search.
   (b) **Bayesian regression:** We perform Bayesian linear/logistic regression to estimate the genetic effects, using a spike-and-slab prior on the effect estimates:

$$P(\beta_j) \sim (1-p_0)\mathcal{N}(0,\sigma^2) + p_0\delta(0). \tag{56}$$

   There is one free hyperparameter in the prior (the sparsity term $1-p_0$) for quantitative traits and two free hyperparameters for binary traits (the sparsity term and h^2). We perform cross-validation to estimate these hyperparameters, splitting the input genotype/phenotype data into 90% for training and 10% for validation and parallelly running the regression using $1-p_0 \in \{0.01, 0.02, 0.05, 0.1, 0.2, 0.5\}$ and, for binary traits, $h^2 \in \{0.01, 0.25, 0.5, 0.75, 0.9\}$. We choose the sparsity (and h^2 for binary traits) parameter that maximizes (in the validation set) the test log-likelihood for the traits.

   After obtaining the optimal hyperparameters for each trait, we retrain the regression using a leave-one-chromosome-out approach. We obtain a $2.5-3\times$ speed-up for the training for this LOCO step by using transfer learning, initializing the effect estimates from the cross-validation run performed using all chromosomes.

2. **Calculation and calibration of association statistics (testing):** Given the estimated genetic effects, we calculate linear mixed-model or logistic mixed-model test statistics up to a constant of proportionality. We rely on optimizations such as just-in-time compilation and code vectorization in Numba. We estimate the constant of proportionality by matching the estimated scaled effective sample size of linear regression on a subset of unrelated homogeneous samples for quantitative traits, and logistic regression on a subset of unrelated homogeneous samples for binary traits.

5 Cost analysis on the UK Biobank RAP

We perform a cost analysis of Quickdraws and other GWAS algorithms using the Research Analysis Platform (RAP), a cloud service provided by the UK Biobank. We compare Quickdraws with BOLT-LMM, Regenie, and FastGWA for quantitative traits and SAIGE, Regenie (with Firth correction), and FastGWA-GLMM for binary traits. We report the total cost and running time for three different sample sizes, $N = 50k$, $N = 405k$, and $N = 1{,}000k$ for 50 quantitative or binary phenotypes. For all methods that require model fitting, we use a filtered set of common genetic markers from the UK Biobank SNP array data set, comprising $M = 458{,}464$ markers for BOLT-LMM, Quickdraws, and Regenie, and use an LD-pruned set of $M = 89{,}177$ markers for SAIGE. We compute test statistics for a filtered set of $\sim$13.3 million imputed genotypes.

To evaluate the cost and the total time to run a GWAS using each method, we consider up to four types of RAP instances, corresponding to different hardware configurations. For $N = 50k$, we consider `mem3_ssd1_v2_x4`, `mem2_ssd1_v2_x8`, and `mem1_ssd1_v2_x16`; for $N = 405k$, we consider `mem2_ssd1_v2_x8`, `mem2_ssd1_v2_x16`, `mem1_ssd1_v2_x36`, and `mem1_ssd1_v2_x72` ; for $N = 1{,}000k$, we only consider `mem1_ssd1_v2_x36`. Additionally, we use `mem2_ssd2_gpu1_x8` to run step 1 of Quickdraws on an instance providing a 24GB Nvidia A10G GPU. All the jobs on RAP were run using "on-demand" priority. Each method was run using configurations that would optimize performance where possible, e.g., by allowing multi-threading and supplying files using the most efficient file formats, and all methods were given the same file containing testing variants as input. While it might be possible to run FastGWA and FastGWA-GLMM in a two-step process using the `--model-only` and `--load-model` flags, we did not explore this option as the majority of the running time is spent on the test statistics calculation, and this approach is unlikely to significantly affect the overall running time. The scripts we used to evaluate the models can be found below.

BOLT-LMM, FastGWA and SAIGE do not leverage multi-trait parallelization, so we run them for 5 phenotypes and extrapolate the results for 50 phenotypes. We also only run BOLT-LMM for one of the three RAP instances, as the large difference in running costs would not to be significantly reduced using other types of hardware. For binary traits and $N = 1{,}000k$, which are more computationally intensive, we also run step 2 for Quickdraws, Regenie, and SAIGE using a subset of $\sim$13.3 million testing variants, and linearly extrapolate the results. The final results for quantitative and binary traits can be found in Supplementary Tables 13, 12, 11 and 10 .

1. FastGWA:

```
#!/bin/bash
./gcta --grm-sparse test_sp_grm \
    --pfile ukbxxxxx_c${chr}_b0_v3 \
    --pheno temp.pheno \
    --qcovar covariates.tab \
    --extract snp_list.txt  \
    --geno 1  --maf 0 --fastGWA-mlm \
    --thread-num $(nproc) \
    --out fastgwa_${chr}
```

2. Regenie:

```
#!/bin/bash
regenie --keep-l0 --step 1 \
    --bed genotype \
    --extract maf001.snps \
    --phenoFile temp.pheno \
    --covarFile covariates.tab \
    --phenoColList "list of phenotypes..." \
    --bsize 1000 --lowmem --lowmem-prefix tmp \
    --out regenie_400k
for chr in {1..22}
do
    regenie --step 2 \
        --pred regenie_400k_pred.list \
        --pgen ukbxxxxx_c${chr}_b0_v3 \
        --sample ${home_dir}ukb_xxxxx.sample \
        --phenoFile temp.pheno \
```

```
            --covarFile covariates.tab \
            --phenoColList "list of phenotypes..." \
            --extract ${home_dir}paper_speed/snp_list.txt \
            --bsize 1000 --lowmem --lowmem-prefix tmp_rg_all2 \
            --out regenie_400k_${chr}
    done
```

3. BOLT-LMM:

```
#!/bin/bash
bolt \
    --bfile="genotype" \
    --phenoFile=temp.pheno --phenoCol=phenotype name \
    --maxMissingPerSnp=0.5 --maxMissingPerIndiv=0.5 \
    --verboseStats --CVfoldsCompute=1 --lmm \
    --LDscoresFile="LDSCORE.1000G_EUR.tab.gz" \
    --covarFile="covariates.tab" --qCovarCol=".." --covarCol=".." \
    --bgenMinMAF=0.001 --bgenMinINFO=0.8 \
    --modelSnps="maf001.snps" \
    --bgenFile="ukbxxxxx_c{1:22}_b0_v3.bgen" \
    --sampleFile="ukb_xxxxx.sample" \
    --statsFile="bolt.sumstats" \
    --statsFileBgenSnps="bolt_imputed.sumstats"
```

4. SAIGE:

```
#!/bin/bash
docker run \
    -e HOME=${home_dir}  \
    -v ${home_dir}:$HOME/ \
    wzhou88/saige:1.1.6.3  step1_fitNULLGLMM.R    \
        --plinkFile=${HOME}/genotype_pruned   \
        --phenoFile=${HOME}/temp.pheno   \
        --phenoCol=phenotype name \
        --covarColList=".." --qCovarColList=".." \
        --sampleIDColinphenoFile=IID \
        --traitType=binary  --LOCO=TRUE \
        --IsOverwriteVarianceRatioFile=TRUE \
        --sparseGRMFile=${HOME}/saige_sp_grm.mtx \
        --sparseGRMSampleIDFile=${HOME}/saige_sp_grm.mtx.sampleIDs.txt \
        --useSparseGRMtoFitNULL=TRUE --isCateVarianceRatio=FALSE \
        --outputPrefix="${HOME}/saige"

for chr in {1..22}
do
    docker run \
        -e HOME=${home_dir}  \
        -v ${home_dir}:$HOME/ \
        wzhou88/saige:1.1.6.3 step2_SPAtests.R      \
            --bgenFile=${HOME}/ukbxxxxx_c10_b0_v3.bgen \
            --bgenFileIndex=${HOME}/ukbxxxxx_c10_b0_v3.bgen.bgi \
            --sampleFile=${HOME}/ukbxxxxx_c10_b0_v3.sample \
            --chrom="${chr}$" --AlleleOrder=ref-first \
            --minMAF=0 --minMAC=10 \
            --GMMATmodelFile="${HOME}/saige.rda" \
            --varianceRatioFile="${HOME}/saige.varianceRatio.txt"   \
            --is_Firth_beta=TRUE    --pCutoffforFirth=0.05 \
            --LOCO=FALSE --is_fastTest=TRUE \
```

```
                        --sparseGRMFile=${HOME}/saige_sp_grm.mtx \
                        --sparseGRMSampleIDFile=${HOME}/saige_sp_grm.mtx.sampleIDs.txt \
                        --idstoIncludeFile="${HOME}/snp_list.txt" \
                        --SAIGEOutputFile="${HOME}/saige.sumstats"
done
```

6 Data analyses and significance testing

In simulation experiments, we tested for differences in FPRs using two-sided t-tests, and for differences in association power using paired t-tests, accounting for the number of simulations and conditions we tested (490) by using an adjusted significance threshold of $p < 0.05/490 = 1.02 \times 10^{-4}$. For real-data analyses, we defined a maximal (approximately) independent set of phenotypes by retaining the maximal set of traits for which all pairwise squared Pearson correlations were less than 0.1 [33]. In case of multiple possible maximal independent sets, we chose the maximal independent set for which a greater number of associations (averaged across Quickdraws, Regenie, FastGWA, and BOLT-LMM) was found. Out of the 129 quantitative and binary phenotypes we analyzed, we found a maximal independent set of 76 phenotypes, which we used for significance testing in further analyses (see Supplementary Table 14). We used a binomial test to assess significance in the number of detected independent loci (Supplementary Tables 6, 7) and the number of replicated loci (Supplementary Tables 8). In particular, we counted the number of tested phenotypes where the number of loci detected using one method was more than those detected using a second method (in case of a tie, both counts were increased by 0.5). We then calculated a binomial p-value for a null model where both methods have an equal chance of detecting more loci.

References

[1] J. Listgarten, C. Lippert, C. M. Kadie, R. I. Davidson, E. Eskin, and D. Heckerman, "Improved linear mixed models for genome-wide association studies," *Nature methods*, vol. 9, no. 6, pp. 525–526, 2012.

[2] J. Yang, N. A. Zaitlen, M. E. Goddard, P. M. Visscher, and A. L. Price, "Advantages and pitfalls in the application of mixed-model association methods," *Nature genetics*, vol. 46, no. 2, pp. 100–106, 2014.

[3] P.-R. Loh *et al.*, "Efficient bayesian mixed-model analysis increases association power in large cohorts," *Nature genetics*, vol. 47, no. 3, pp. 284–290, 2015.

[4] P.-R. Loh, G. Kichaev, S. Gazal, A. P. Schoech, and A. L. Price, "Mixed-model association for biobank-scale datasets," *Nature genetics*, vol. 50, no. 7, pp. 906–908, 2018.

[5] L. Jiang *et al.*, "A resource-efficient tool for mixed model association analysis of large-scale data," *Nature genetics*, vol. 51, no. 12, pp. 1749–1755, 2019.

[6] Y. Wu and S. Sankararaman, "A scalable estimator of snp heritability for biobank-scale data," *Bioinformatics*, vol. 34, no. 13, pp. i187–i194, 2018.

[7] A. Pazokitoroudi *et al.*, "Efficient variance components analysis across millions of genomes," *Nature communications*, vol. 11, no. 1, pp. 1–10, 2020.

[8] J. Zhu *et al.*, "Fast variance component analysis using large-scale ancestral recombination graphs," *bioRxiv*, 2024. DOI: 10.1101/2024.08.31.610262.

[9] D. M. Blei, A. Kucukelbir, and J. D. McAuliffe, "Variational inference: A review for statisticians," *Journal of the American statistical Association*, vol. 112, no. 518, pp. 859–877, 2017.

[10] C. M. Bishop and N. M. Nasrabadi, *Pattern recognition and machine learning*. Springer, 2006, vol. 4.

[11] J. Spence, "Flexible mean field variational inference using mixtures of non-overlapping exponential families," *Advances in Neural Information Processing Systems*, vol. 33, pp. 19 642–19 654, 2020.

[12] M. D. Hoffman, D. M. Blei, C. Wang, and J. Paisley, "Stochastic variational inference," *Journal of Machine Learning Research*, 2013.

[13] H. Robbins and S. Monro, "A stochastic approximation method," *The annals of mathematical statistics*, pp. 400–407, 1951.

[14] D. P. Kingma and M. Welling, "Auto-encoding variational bayes," *arXiv preprint arXiv:1312.6114*, 2013.

[15] D. P. Kingma, T. Salimans, and M. Welling, "Variational dropout and the local reparameterization trick," *Advances in neural information processing systems*, vol. 28, 2015.

[16] T. Dettmers, M. Lewis, S. Shleifer, and L. Zettlemoyer, "8-bit optimizers via block-wise quantization," *arXiv preprint arXiv:2110.02861*, 2021.

[17] C. J. Maddison, A. Mnih, and Y. W. Teh, "The concrete distribution: A continuous relaxation of discrete random variables," *arXiv preprint arXiv:1611.00712*, 2016.

[18] E. Jang, S. Gu, and B. Poole, "Categorical reparameterization with gumbel-softmax," *arXiv preprint arXiv:1611.01144*, 2016.

[19] J. Hammersley and K. Morton, "A new monte carlo technique: Antithetic variates," in *Mathematical proceedings of the Cambridge philosophical society*, Cambridge University Press, vol. 52, 1956, pp. 449–475.

[20] C. Blundell, J. Cornebise, K. Kavukcuoglu, and D. Wierstra, "Weight uncertainty in neural networks," *arXiv preprint arXiv:1505.05424*, 2015.

[21] O. Shayer, D. Levi, and E. Fetaya, "Learning discrete weights using the local reparameterization trick," *arXiv preprint arXiv:1710.07739*, 2017.

[22] G. R. Svishcheva, T. I. Axenovich, N. M. Belonogova, C. M. Van Duijn, and Y. S. Aulchenko, "Rapid variance components–based method for whole-genome association analysis," *Nature genetics*, vol. 44, no. 10, pp. 1166–1170, 2012.

[23] B. K. Bulik-Sullivan *et al.*, "Ld score regression distinguishes confounding from polygenicity in genome-wide association studies," *Nature genetics*, vol. 47, no. 3, pp. 291–295, 2015.

[24] J. Mbatchou *et al.*, "Computationally efficient whole-genome regression for quantitative and binary traits," *Nature genetics*, vol. 53, no. 7, pp. 1097–1103, 2021.

[25] J. Yang *et al.*, “Genomic inflation factors under polygenic inheritance,” *European journal of human genetics*, vol. 19, no. 7, pp. 807–812, 2011.

[26] A. Ziyatdinov *et al.*, “Estimating the effective sample size in association studies of quantitative traits,” *G3*, vol. 11, no. 6, jkab057, 2021.

[27] A. Manichaikul, J. C. Mychaleckyj, S. S. Rich, K. Daly, M. Sale, and W.-M. Chen, “Robust relationship inference in genome-wide association studies,” *Bioinformatics*, vol. 26, no. 22, pp. 2867–2873, 2010.

[28] C. C. Chang, C. C. Chow, L. C. Tellier, S. Vattikuti, S. M. Purcell, and J. J. Lee, “Second-generation plink: Rising to the challenge of larger and richer datasets,” *Gigascience*, vol. 4, no. 1, s13742–015, 2015.

[29] J. Ansel *et al.*, “Pytorch 2: Faster machine learning through dynamic python bytecode transformation and graph compilation,” in *Proceedings of the 29th ACM International Conference on Architectural Support for Programming Languages and Operating Systems, Volume 2*, 2024, pp. 929–947.

[30] S. K. Lam, A. Pitrou, and S. Seibert, “Numba: A llvm-based python jit compiler,” in *Proceedings of the Second Workshop on the LLVM Compiler Infrastructure in HPC*, 2015, pp. 1–6.

[31] S. Koranne and S. Koranne, “Hierarchical data format 5: Hdf5,” *Handbook of open source tools*, pp. 191–200, 2011.

[32] C. R. Harris *et al.*, “Array programming with NumPy,” *Nature*, vol. 585, no. 7825, pp. 357–362, Sep. 2020. DOI: `10.1038/s41586-020-2649-2`. [Online]. Available: `https://doi.org/10.1038/s41586-020-2649-2`.

[33] S. Gazal *et al.*, “Functional architecture of low-frequency variants highlights strength of negative selection across coding and non-coding annotations,” *Nature genetics*, vol. 50, no. 11, pp. 1600–1607, 2018.

[34] C. Bycroft *et al.*, “The uk biobank resource with deep phenotyping and genomic data,” *Nature*, vol. 562, no. 7726, pp. 203–209, 2018.

[35] L. Jostins *et al.*, “Host–microbe interactions have shaped the genetic architecture of inflammatory bowel disease,” *Nature*, vol. 491, no. 7422, pp. 119–124, 2012.

[36] M.-A. of Glucose *et al.*, “Large-scale association analysis provides insights into the genetic architecture and pathophysiology of type 2 diabetes,” *Nature genetics*, vol. 44, no. 9, pp. 981–990, 2012.

[37] P. C. Dubois *et al.*, “Multiple common variants for celiac disease influencing immune gene expression,” *Nature genetics*, vol. 42, no. 4, pp. 295–302, 2010.

[38] M. Nagel *et al.*, “Meta-analysis of genome-wide association studies for neuroticism in 449,484 individuals identifies novel genetic loci and pathways,” *Nature genetics*, vol. 50, no. 7, pp. 920–927, 2018.

[39] K. M. De Lange *et al.*, “Genome-wide association study implicates immune activation of multiple integrin genes in inflammatory bowel disease,” *Nature genetics*, vol. 49, no. 2, pp. 256–261, 2017.

Appendix

KL-divergence for non-overlapping mixture distributions

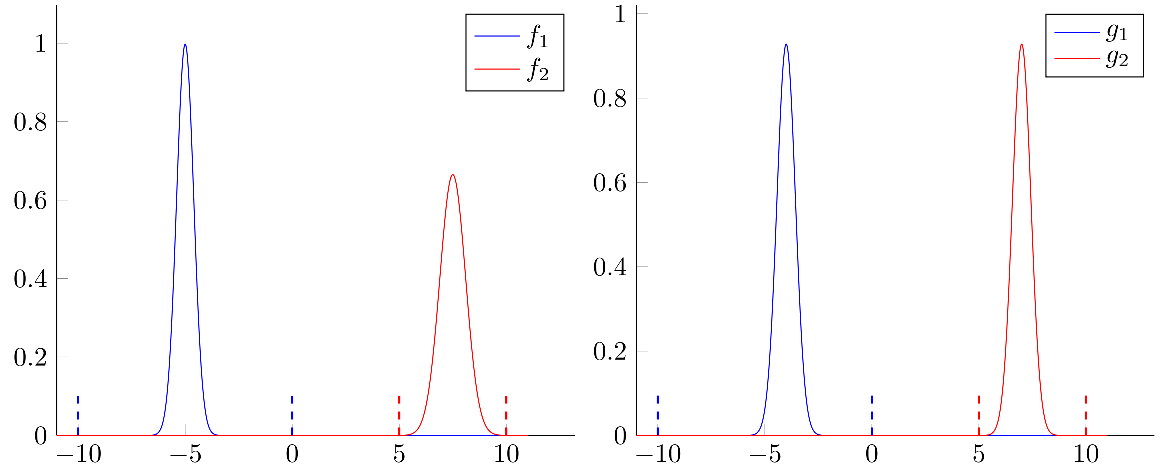

Posterior distribution   Prior distribution

Theorem 1: Let $q = \sum_i^N \pi_i f_i$ be a mixture of non-overlapping distributions, i.e., $S_i \cap S_j = \emptyset, \forall i \neq j$, where S_i is the support of distribution f_i. If p is a probability distribution and can be represented as a mixture of non-overlapping distributions with the same support as f_i, i.e., $p = \sum_i^N \pi'_i g_i$ and support of g_i = support of f_i, the KL divergence between q and p can be simplified as follows:

$$KL(q||p) = \sum_i^N \left(\pi_i KL(f_i||g_i) + \pi_i \log(\pi_i) - \pi_i \log(\pi'_i)\right). \tag{57}$$

Proof: Assuming q is 0 outside $\cup_i^N S_i$, we can write the integral as

$$\begin{aligned} KL(q||p) &= \int q \log(q) - q \log(p) = \sum_i^N \int_{S_i} q \log(q) - q \log(p) \\ &= \sum_i^N \int_{S_i} \left(\sum_i^N \pi_i f_i\right) \log\left(\sum_i^N \pi_i f_i\right) - \left(\sum_i^N \pi_i f_i\right) \log\left(\sum_i^N \pi'_i g_i\right). \end{aligned} \tag{58}$$

Because $S_i \cap S_j = \emptyset, \forall i \neq j$, we can simplify as

$$\begin{aligned} KL(q||p) &= \sum_i^N \int_{S_i} \pi_i f_i \log(\pi_i f_i) - \pi_i f_i \log(\pi'_i g_i) \\ &= \sum_i^N \pi_i \left(\int_{S_i} f_i \log(f_i) - f_i \log(g_i)\right) + \sum_i^N \left(\pi_i log(\pi_i) \int_{S_i} f_i - \pi_i log(\pi'_i) \int_{S_i} f_i\right) \\ &= \sum_i^N \pi_i \left(\int_{S_i} f_i \log(f_i) - f_i \log(g_i)\right) + \sum_i^N \left(\pi_i log(\pi_i) - -\pi_i log(\pi'_i)\right) \\ &= \sum_i^N \left(\pi_i KL(f_i||g_i) + \pi_i \log(\pi_i) - \pi_i \log(\pi'_i)\right). \end{aligned} \tag{59}$$

Supplementary Tables

Table 1: (See Excel sheet.) **Complete list of False Positive Rates (FPRs) in simulations.**

Table 2: **Summary of calibration in simulations using FastGWA with up to 460k self-identified Europeans.** The table presents the false positive rate (FPR) for FastGWA under various sample sizes, controlling for residual population stratification by adjusting the number of included principal components (PCs) or by omitting them entirely. We report the FPR at a significance threshold of $\alpha = 0.005$, calculated as the fraction of variants on even chromosomes with p-value lower than α. The polygenicity of the simulated trait is fixed at 1%. S.E. refers to the standard error, and * indicates a Bonferroni-significant two-sided t-test p-value. Mean FPR refers to the mean across 50 independent simulated traits, SE refers to standard errors, and two-sided t-test p-values are computed for the significance of a deviation from the FPR threshold. Each simulation condition varied one of the following baseline parameters: sample size (N = 460k), ancestry differences explaining 5% of phenotypic variance, and the use of 10 PCs to correct for stratification.

**Condition**	**Mean FPR**	**S.E. FPR**	**t-test p-value**
**Varying sample sizes**			
N = 50k	0.00503	2.99×10^{-5}	0.23
N = 100k	0.00514	4.35×10^{-5}	0.0009
N = 460k	0.00586	4.11×10^{-5}	8×10^{-38} *
**Varying pop. stratification**			
5%	0.00586	4.11×10^{-5}	8×10^{-38} *
0%	0.00509	2.94×10^{-5}	0.0019
**Varying #PCs**			
3 PCs	0.01424	3.23×10^{-4}	2×10^{-48} *
10 PCs	0.00586	4.11×10^{-5}	8×10^{-38} *
20 PCs	0.00531	3.61×10^{-5}	1.8×10^{-13} *

Table 3: **Summary of calibration in simulations using Quickdraws for (a) $N = 405$k white British individuals and (b) $N = 460$k European individuals [34].** We report the false positive rate (FPR) at a significance threshold of $\alpha \in \{0.05, 0.0005\}$, calculated as the fraction of variants on even chromosomes with p-value lower than α. We varied the polygenicity from 1% to 10% in quantitative traits. For binary traits, we changed the prevalence from 0.3 to 0.001, fixing the polygenicity to 2%. Mean FPR refers to the mean across 50 independent simulated traits, SE refers to standard errors, and two-sided t-test p-values are computed for the significance of a deviation from the FPR threshold.

Simulation Type	FPR Threshold	Polygenicity (QT) / Prevalence (BT)	Mean FPR	SE FPR	T-test P
Quantitative Traits	0.05	1%	0.05034	0.00022	0.13382
		2%	0.05040	0.00029	0.17865
		10%	0.05036	0.00017	0.03658
	0.0005	1%	0.00052	0.00001	0.06363
		2%	0.00053	0.00002	0.04845
		10%	0.00052	0.00001	0.04787
Binary Traits	0.05	0.3	0.04923	0.00022	0.00103
		0.1	0.04986	0.00019	0.46758
		0.01	0.04940	0.00016	0.00044
		0.001	0.04648	0.00016	7.39×10^{-39}
	0.0005	0.3	0.00048	0.00001	0.05193
		0.1	0.00051	0.00001	0.57445
		0.01	0.00046	0.00001	0.00018
		0.001	0.00042	0.00001	5.28×10^{-13}

(a) $N = 405$k white British individuals.

Simulation Type	FPR Threshold	Polygenicity (QT) / Prevalence (BT)	Mean FPR	SE FPR	T-test P
Quantitative Traits	0.05	1%	0.05061	0.000231	0.0098
		2%	0.05019	0.00025	0.4444
		10%	0.05037	0.00026	0.1709
	0.0005	1%	0.00053	0.00001	0.0075
		2%	0.00053	0.00001	0.01728
		10%	0.00053	0.00001	0.0429
Binary Traits	0.05	0.3	0.04983	0.00015	0.3108
		0.1	0.04997	0.00017	0.9015
		0.01	0.0437	0.00058	5.19×10^{-18}
		0.001	0.03121	0.0022	3.72×10^{-13}
	0.0005	0.3	0.00051	0.00001	0.1391
		0.1	0.00053	0.00002	0.0436
		0.01	0.00033	0.00003	4.93×10^{-7}
		0.001	0.00022	0.00003	2.47×10^{-15}

(b) $N = 460$k European individuals.

Table 4: **Quantitative traits analyzed.** Quantitative traits analyzed, including RHE-MC h^2 estimates and sample sizes (N).

**UKBB ID**	**Phenotype**	h^2	**N**
30850	Testosterone	0.03	355373
1438	Bread intake	0.053	396396
874	Duration of walks	0.054	346657
30740	Glucose	0.088	359138
2217	Age started wearing glasses	0.092	349337
30060	Mean corp. haemoglobin conc.	0.101	398336
30810	Phosphate	0.149	358861
30780	LDL direct	0.152	390888
3064	Peak expiratory flow (PEF)	0.152	370024
30690	Cholesterol	0.157	391567
30020	Haemoglobin conc	0.158	398342
30030	Haematocrit %	0.161	398343
46	Hand grip strength (left)	0.162	403800
47	Hand grip strength (right)	0.164	403850
30600	Albumin	0.168	359528
30670	Urea	0.172	391307
30680	Calcium	0.177	359391
30650	Asp-aminotransferase	0.178	390183
30860	Total protein	0.188	359122
4079	Diastolic blood pressure	0.188	379181
30640	Apolipoprotein B	0.189	389718
30200	Neutrophill %	0.19	397665
4080	Systolic blood pressure	0.192	379174
102	Pulse rate	0.194	379181
30180	Lymphocyte %	0.196	397665
30700	Creatinine	0.198	391377
30830	SHBG	0.2	356165
30880	Urate	0.206	391102
30630	Apolipoprotein A	0.213	357411
30150	Eosinophill count	0.213	397661
30790	Lipoprotein A	0.22	312270
30140	Neutrophill count	0.22	397661
30130	Monocyte %	0.221	397665
30870	Triglycerides	0.234	391260
30730	Gamma glutamyltransferase	0.24	391372
30710	C-reactive protein	0.241	390752
20257	Forced vital capacity (FVC) Z-score	0.242	328403
30750	Glycated haemoglobin	0.245	391597
30760	HDL cholesterol	0.248	359370
30210	Eosinophill %	0.251	397665
30130	Monocyte count	0.253	397661
30280	Immature reticulocyte fraction	0.26	391990
30250	Reticulocyte count	0.26	392210
20256	Forced expiratory volume (FEV1) Z-score	0.263	328403
30070	Red blood cell distribution width	0.264	398342
30260	Mean reticulocyte vol	0.266	392211
30240	Reticulocyte %	0.267	392210
20258	FEV1/FVC ratio Z-score	0.269	328403
30120	Lymphocyte count	0.269	397661
48	Waist circumference	0.277	404969
30010	Red blood cell count	0.279	398343

Continued on next page

Table 4: *(Continued)*

**UKBB ID**	**Phenotype**	h^2	**N**
30610	Alkaline phosphatase	0.279	391586
30300	High light scatter reticulocyte count	0.289	391989
30770	IGF-1	0.29	389525
30270	Mean sphered cell vol	0.293	391990
23115	Leg fat % (left)	0.294	399130
23111	Leg fat % (right)	0.294	399151
30110	Platelet distribution width	0.296	398153
23127	Trunk fat %	0.302	398940
23119	Arm fat % (right)	0.302	399098
23123	Arm fat % (left)	0.305	399037
30290	High light scatter reticulocyte %	0.306	391990
23099	Body fat %	0.310	398955
49	Hip circumference	0.318	404972
30090	Platelet crit	0.326	398154
30840	Total bilirubin	0.327	389995
23110	Impedance of arm (left)	0.331	399149
23109	Impedance of arm (right)	0.331	399133
23128	Trunk fat mass	0.331	398918
21001	Body mass index (BMI)	0.348	405010
23106	Impedance of whole body	0.367	399144
30080	Platelet count	0.373	398339
21002	Weight	0.381	405018
30040	Mean corp. vol	0.414	398342
23105	Basal metabolic rate	0.419	399169
23102	Whole body water mass	0.428	399179
30050	Mean corp. haemoglobin	0.432	398339
30100	Mean platelet vol	0.448	398334
50	Standing height	0.745	405035

Table 5: **Self-reported disease traits analyzed.** Self-reported disease traits analyzed, including their disease code and prevalence.

UKBB ID	Disease ID	Phenotype	Prevalence
20002	1065	Hypertension	0.2740
20002	1473	High cholestrol	0.1400
20002	1111	Asthma	0.1190
20002	1465	Osteoarthritis	0.0946
20002	1387	Hayfever	0.0667
20002	1286	Depression	0.0649
20002	1138	Gastric reflux	0.0529
20002	1226	Hypothyroidism	0.0518
20002	1220	Diabetes	0.0421
20002	1265	Migraine	0.0343
20002	1074	Angina	0.0335
20002	1452	Eczema	0.0320
20002	1474	Hiatus hernia	0.0266
20002	1075	Heart attack	0.0247
20001	1002	Breast cancer	0.0241
20002	1094	Deep venous thrombosis	0.0210
20002	1162	Cholelithiasis	0.0188
20002	1294	Back problem	0.0183
20002	1396	Enlarged prostate	0.0183
20002	1309	Osteoporosis	0.0182
20002	1287	Anxiety	0.0182
20002	1351	Uterine fibroids	0.0170
20002	1538	Arthritis nos	0.0148
20002	1113	Emphysema	0.0142
20002	1458	Diverticulitis	0.0137
20002	1453	Psoriasis	0.0131
20002	1277	Glaucoma	0.0125
20001	1061	Basal cell carcinoma	0.0120
20001	1044	Prostate cancer	0.0097
20002	1223	Type 2 diabetes	0.0093
20002	1197	Bladder stone	0.0092
20002	1093	Pulmonary embolism	0.0088
20001	1059	Malignant melanoma	0.0087
20002	1225	Hyperthyroidism	0.0084
20002	1202	Urinary frequency	0.0069
20002	1353	Vaginal prolapse	0.0069
20002	1330	Iron deficiency anaemia	0.0065
20002	1463	Ulcerative colitis	0.0056
20002	1417	Nasal polyps	0.0050
20002	1295	Joint disorder	0.0049
20002	1456	Coeliac disease	0.0047
20002	1112	Chronic obstructive airways disease	0.0045
20002	1281	Retinal detachment	0.0041
20002	1123	Sleep apnoea	0.0038
20002	1331	Pernicious anaemia	0.0033
20002	1462	Crohns disease	0.0032
20002	1291	Bipolar disorder	0.0028
20002	1446	Gout	0.0026
20002	1661	Vitiligo	0.0005
20002	1430	Hypopituitarism	0.0004

Table 6: **Number of independent associated loci for quantitative traits.** Number of independent associated loci for quantitative traits after Plink clumping using summary statistics from FastGWA, Regenie, BOLT-LMM, and Quickdraws

Phenotype	FastGWA	Regenie	BOLT-MoG	Quickdraws
Testosterone	72	65	83	40
Bread intake	5	5	6	5
Duration of walks	0	0	1	0
Glucose	87	92	94	93
Age started wearing glasses	30	30	29	32
Mean corp. haemoglobin conc.	82	85	86	86
Phosphate	131	140	149	140
LDL direct	151	165	170	164
Peak expiratory flow (PEF)	82	85	83	85
Cholesterol	181	192	197	191
Haemoglobin conc	306	341	363	356
Haematocrit %	276	305	317	316
Hand grip strength (left)	75	78	79	84
Hand grip strength (right)	76	89	91	91
Albumin	198	216	222	220
Urea	145	158	161	156
Calcium	178	196	205	198
Asp-aminotransferase	254	271	282	276
Total protein	250	277	296	290
Diastolic blood pressure	129	143	151	146
Apolipoprotein B	178	205	214	206
Neutrophill %	266	290	304	306
Systolic blood pressure	150	157	162	154
Pulse rate	171	182	186	187
Lymphocyte %	305	332	344	345
Creatinine	351	400	418	403
SHBG	275	334	348	328
Urate	228	262	272	265
Apolipoprotein A	252	306	319	294
Eosinophill count	299	334	345	356
Lipoprotein A	54	65	70	56
Neutrophill count	299	330	341	356
Monocyte %	362	416	437	449
Triglycerides	234	287	288	294
Gamma glutamyltransferase	287	323	330	327
C-reactive protein	196	227	233	227
Forced vital capacity (FVC) Z-score	163	185	203	193
Glycated haemoglobin	345	400	418	408
HDL cholesterol	280	353	371	352
Eosinophill %	375	430	463	461
Monocyte count	402	470	490	494
Immature reticulocyte fraction	234	258	262	264
Reticulocyte count	311	361	372	367
Forced expiratory volume (FEV1) Z-score	189	218	228	228
Red blood cell distribution width	368	423	443	425
Mean reticulocyte vol	395	439	463	460
Reticulocyte %	307	353	363	361
FEV1/FVC ratio Z-score	272	301	323	307
Lymphocyte count	353	406	422	420
Waist circumference	186	206	214	212

Continued on next page

Table 6: *(Continued)*

**Phenotype**	**FastGWA**	**Regenie**	**BOLT-MoG**	**Quickdraws**
Alkaline phosphatase	355	446	473	457
Red blood cell count	409	482	507	508
High light scatter reticulocyte count	325	373	393	390
IGF-1	364	435	459	445
Mean sphered cell vol	378	430	456	456
Leg fat % (left)	197	222	233	233
Leg fat % (right)	204	220	229	235
Platelet distribution width	456	549	576	591
Trunk fat %	197	224	238	237
Arm fat % (right)	215	240	258	251
Arm fat % (left)	206	242	256	246
High light scatter reticulocyte %	324	369	398	399
Body fat %	215	242	259	255
Hip circumference	255	292	308	306
Platelet crit	485	587	623	626
Total bilirubin	146	203	206	199
Impedance of arm (left)	257	292	309	307
Impedance of arm (right)	301	368	387	366
Trunk fat mass	302	367	384	373
Body mass index (BMI)	262	309	334	340
Impedance of whole body	354	451	488	452
Platelet count	539	670	720	721
Weight	329	398	428	423
Mean corp. vol	487	580	599	605
Basal metabolic rate	465	545	592	584
Whole body water mass	489	587	638	627
Mean corp. haemoglobin	429	538	562	569
Mean platelet vol	618	821	892	887
Standing height	1022	1327	1452	1674
Total	21,380	24,995	26,368	26,236

Table 7: **Number of independent associated loci for binary traits.** Number of independent associated loci for binary traits after Plink clumping using summary statistics from FastGWA-GLMM, SAIGE, Regenie, and Quickdraws. We excluded traits (enlarged prostate, prostate cancer, vaginal prolapse, and uterine fibroids) which led to matrix inversion errors or non-convergence of Firth logistic regression, or for which no associations were found using any method.

**Phenotype**	**FastGWA-GLMM**	**SAIGE**	**Regenie**	**Quickdraws**
Hypertension	155	156	170	172
High cholesterol	52	52	56	57
Asthma	65	65	63	63
Osteoarthritis	2	2	2	2
Hayfever	20	21	20	20
Depression	1	1	1	1
Gastric reflux	1	1	1	1
Hypothyroidism	80	81	81	84
Diabetes	36	36	36	38
Migraine	9	9	8	9
Angina	8	9	9	8
Eczema	13	13	13	13
Hiatus hernia	0	0	0	0
Heart attack	9	8	9	9
Breast cancer	11	11	11	11
Deep venous thrombosis	9	9	9	9
Cholelithiasis	11	11	12	11
Osteoporosis	8	8	9	8
Diverticulitis	1	1	1	1
Psoriasis	21	21	18	25
Glaucoma	6	6	6	6
Basal cell carcinoma	12	12	12	12
Type 2 diabetes	2	2	2	2
Bladder stone	1	1	1	1
Pulmonary embolism	5	5	5	6
Malignant melanoma	3	3	2	3
Hyperthyroidism	12	12	11	12
Ulcerative colitis	8	8	8	8
Nasal polyps	5	5	6	6
Celiac disease	24	26	30	32
Chronic obstructive airways disease	1	1	0	1
Pernicious anaemia	1	1	2	2
Crohns disease	2	2	2	3
Total	594	599	616	636

Table 8: (See Excel sheet.) **Complete list of replication rates and number of replicated variants or loci across traits.**

Table 9: **Number of individual variants replicated using trait-specific summary statistics.** Number of individual variants replicated using summary statistics for Crohn's disease [35], Type 2 Diabetes [36], Celiac disease [37], Depression [38], and Ulcerative Colitis [39]. We use a discovery threshold of 5×10^{-8} and vary the replication threshold from 5×10^{-2} to 5×10^{-6}, and compare summary statistics from FastGWA-GLMM, Regenie, and Quickdraws.

Phenotype	Method	Repl. thresh. $= 5 \times 10^{-2}$		Repl. thresh. $= 5 \times 10^{-4}$		Repl. thresh. $= 5 \times 10^{-6}$	
		# repl.	repl. ratio.	# repl.	repl. ratio.	# repl.	repl. ratio.
Crohns disease	fastGWA	29	1	29	1	29	1
Crohns disease	Regenie	29	1	29	1	29	1
Crohns disease	Quickdraws	29	1	29	1	29	1
Type 2 diabetes	fastGWA	2	1	2	1	2	1
Type 2 diabetes	Regenie	2	1	2	1	2	1
Type 2 diabetes	Quickdraws	2	1	2	1	2	1
Coeliac disease	fastGWA	18	1	18	1	17	0.94
Coeliac disease	Regenie	20	1	20	1	19	0.95
Coeliac disease	Quickdraws	23	1	23	1	22	0.96
Depression	fastGWA	1	1	1	1	1	1
Depression	Regenie	0	0	0	0	0	0
Depression	Quickdraws	2	1	2	1	2	1
Ulcerative colitis	fastGWA	24	1	24	1	24	1
Ulcerative colitis	Regenie	25	1	25	1	25	1
Ulcerative colitis	Quickdraws	23	1	23	1	23	1
Total	fastGWA	74	1	74	1	73	0.99
Total	Regenie	76	1	76	1	75	0.99
Total	Quickdraws	79	1	79	1	78	0.99

Table 10: **Computational efficiency of Quickdraws for N = 1,000,000 samples.** We compare the computational requirements of Quickdraws and Regenie when computing summary statistics for 458k, 13.3 million and 600 million variants, 50 quantitative, and 50 binary traits, using N = 1,000,000 and 458,464 genotyped markers for model fitting. The data set was generated by duplicating samples from the $N = 405k$ subset of white British individuals. * indicates that the cost of imputed variant association testing is extrapolated from the testing time for $458,464$ genotyped markers. Quickdraws was run using the low-memory option. Running times and costs were computed using the same hardware for both methods (mem1_ssd1_v2_x36, 72 GB of RAM, 36-core processor).

**Method**	**Quantitative Traits**		**Binary Traits**	
	**Time (hrs)**	**Cost (£)**	**Time (hrs)**	**Cost (£)**
Regenie (Step 1)	13.38	12.11	178.41	159.29
Regenie (Step 2, M = 458k)	2.13	1.90	32.96	29.43
Regenie* (Step 2, M = 13.3 mil.)	62.04	55.39	957.33	854.71
Regenie* (Step 2, M = 600 mil.)	2798.8	2498.8	43187.82	38558.34
Quickdraws (Step 1)	243.23	161.50	424.56	281.91
Quickdraws (Step 2, M = 458k)	1.51	1.36	23.4	21.19
Quickdraws* (Step 2, M = 13.3 mil.)	44.04	39.77	679.51	615.43
Quickdraws* (Step 2, M = 600 mil.)	1986.76	1794.13	30654.58	27763.75

Table 11: **Computational efficiency of Quickdraws for a single quantitative or binary trait.** We compare the computational requirements of Regenie and Quickdraws, which support the parallal analysis of multiple traits, with those of FastGWA, BOLT-LMM, and SAIGE. All methods are used to compute summary statistics for ∼13.3 million variants and one phenotype with N = 405,088 and 458,464 genotyped markers for model fitting ($89,177$ genotyped markers for SAIGE, see Methods). Running times and costs are computed using the same hardware for all methods (mem1_ssd1_v2_x36, 72 GB of RAM, 36-core processor).

**Method**	**Quantitative Traits**		**Binary Traits**	
	**Time (hrs)**	**Cost (£)**	**Time (hrs)**	**Cost (£)**
Quickdraws	71.92	57.86	141.11	109.5
Regenie	26.71	22.38	24.73	22.40
BOLT-LMM	168.0	150.0	-	-
FastGWA	2.6	2.29	5.08	4.536
SAIGE	-	-	240.48	214.72

Table 12: **Computational efficiency of Quickdraws for binary trait association.** We compare the computational requirements of several GWAS algorithms and Quickdraws across 3 to 4 RAP cloud instances for ∼13.3 million tested variants and 50 phenotypes with either $N = 50{,}000$ or $N = 405{,}088$ samples and 458,464 genotyped markers for model fitting. * The step 1 for Quickdraws is run using `mem3_ssd1_gpu_x8`. All methods except FastGWA-GLMM were run on 1 million test variants and extrapolated. Additional details may be found in Section 5.

Sample	Instance	Quickdraws* (£)	Regenie (£)	FastGWA-GLMM (£)	SAIGE (£)
50k	mem3_ssd1_v2_x4	30.5	44.20	9.28	**47.54**
	mem2_ssd1_v2_x8	29.79	41.62	**9.20**	54.48
	mem1_ssd1_v2_x16	**25.221**	**40.99**	11.64	101.94
405k	mem2_ssd1_v2_x8	Disk space exceeded	Disk space exceeded	**98.1**	**2834.91**
	mem2_ssd1_v2_x16	**395.937**	**506.85**	126.04	5703.49
	mem1_ssd1_v2_x36	571.964	769.17	226.82	10736.36
	mem1_ssd1_v2_x72	628.003	1192.22	319.66	21887.59

(a) Total cost (GBP) on UK Biobank RAP.

Sample	Instance	Quickdraws* (h)	Regenie (h)	FastGWA-GLMM (h)	SAIGE (h)
50k	mem3_ssd1_v2_x4	143.87	300.78	63.58	322.24
	mem2_ssd1_v2_x8	96.14	182.03	40.63	272.97
	mem1_ssd1_v2_x16	51.21	100.49	29.34	253.28
405k	mem2_ssd1_v2_x8	Disk space exceeded	Disk space exceeded	433.29	12524.16
	mem2_ssd1_v2_x16	798.55	930.32	278.35	12584.25
	mem1_ssd1_v2_x36	682.33	869.96	254.06	12024.41
	mem1_ssd1_v2_x72	453.89	663.53	179.02	12264.12

(b) Total running time (hours).

Table 13: **Computational efficiency of Quickdraws for quantitative trait association.** We compare the computational requirements of several GWAS algorithms and Quickdraws across 3 to 4 RAP cloud instances for ∼13.3 million tested variants and 50 phenotypes with either N = 50,000 or N = 405,088 samples and 458,464 genotyped markers for model fitting. * The step 1 for Quickdraws is run using `mem3_ssd1_gpu_x8`; ** extrapolated value; "-" indicates that BOLT-LMM was not run in this setting. Additional details may be found in Section 5.

Sample	Instance	Quickdraws* (£)	Regenie (£)	FastGWA (£)	BOLT-LMM (£)
50k	mem3_ssd1_v2_x4	**7.9**	**3.95**	**3.63**	-
	mem2_ssd1_v2_x8	8.397	4.65	4.58	**158.38**
	mem1_ssd1_v2_x16	9.018	6.18	7.01	-
405k	mem2_ssd1_v2_x8	Disk space exceeded	Disk space exceeded	**37.31**	Out of Memory
	mem2_ssd1_v2_x16	**92.993**	**24.18**	59.28	-
	mem1_ssd1_v2_x36	110.91	44.66	114.61	**7500.0**
	mem1_ssd1_v2_x72	156.908	68.72	161.88	-

(a) Total cost (GBP) on UK Biobank RAP.

Sample	Instance	Quickdraws* (h)	Regenie (h)	FastGWA (h)	BOLT-MoG (h)
50k	mem3_ssd1_v2_x4	20.18	27.07	24.84	-
	mem2_ssd1_v2_x8	18.57	20.55	20.24	798.5**
	mem1_ssd1_v2_x16	16.27	15.61	17.66	-
405k	mem2_ssd1_v2_x8	Disk space exceeded	Disk space exceeded	164.77	Out of Memory
	mem2_ssd1_v2_x16	159.78	53.4	130.91	-
	mem1_ssd1_v2_x36	149.29	50.02	128.37	8400**
	mem1_ssd1_v2_x72	149.29	38.5	90.66	-

(b) Total running time (hours).

Table 14: **List of 76 approximately independent traits.** List of 71 approximately independent traits, for which the pairwise squared phenotypic correlation between traits is ≤ 0.1.

**Binary Traits**	**Quantitative Traits**
Hypertension	Eosinophill %
Asthma	Lymphocyte count
Diabetes	Mean platelet thrombocyte volume
High cholestrol	Mean reticulocyte volume
Hypothyroidism	Monocyte count
Osteoarthritis	Neutrophill count
Depression	Platelet crit
Hayfever	Red blood cell erythrocyte count
Migraine	Red blood cell erythrocyte distribution width
Hiatus hernia	Reticulocyte %
Breast cancer	Alkaline phosphatase
Diverticulitis	Apolipoprotein A
Gastric reflux	Aspartate aminotransferase
Osteoporosis	C-reactive protein
Basal cell carcinoma	Glucose
Celiac disease	IGF-1
Bladder stone	LDL direct
Ulcerative colitis	Lipoprotein A
Deep venous thrombosis	Phosphate
Glaucoma	Total bilirubin
Vitiligo	Total protein
Cholelithiasis	Triglycerides
Gout	Urea
Crohns disease	Pulse rate
Back problem	Bread intake
Emphysema	Forced vital capacity (FVC) Z-score
Psoriasis	FEV1/ FVC ratio Z-score
Eczema	Leg fat % (left)
Iron deficiency anaemia	Systolic blood pressure
Chronic obstructive airways disease	Duration of walks
Hyperthyroidism	Age started wearing glasses
Type 2 diabetes	
Sleep apnoea	
Malignant melanoma	
Bipolar disorder	
Pernicious anaemia	
Nasal polyps	
Anxiety	
Arthritis nos	
Retinal detachment	
Pulmonary embolism	
Joint disorder	
Hypopituitarism	
Urinary frequency	
Heart attack	

Supplementary Figures

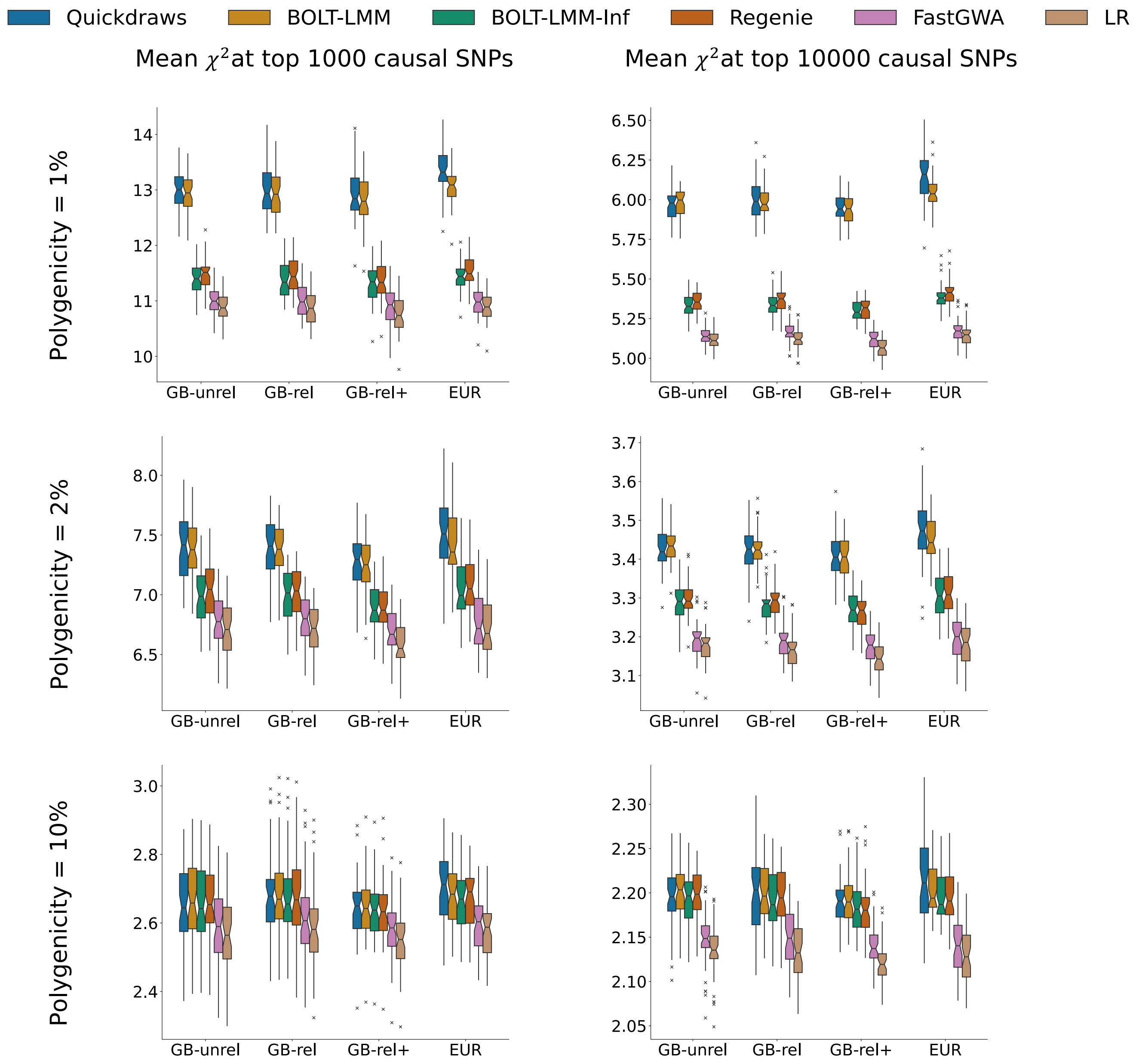

Figure 1: **Summary of statistical power in simulations for quantitative traits.** We measure the normalized causal χ^2 at top 1,000 and top 10,000 causal variants. To correct for confounding, the causal χ^2 is normalized by the average χ^2 at null variants on even chromosomes. The line inside each box indicates the median value, the central box indicates the interquartile range, whiskers indicate data up to 1.5 times the IQR, and outliers are shown as separate points. These results are based on simulations of 50 independent traits. A description of the group labels (GB-unrel, GB-rel, GB-rel+, EUR) is provided in the Methods section.

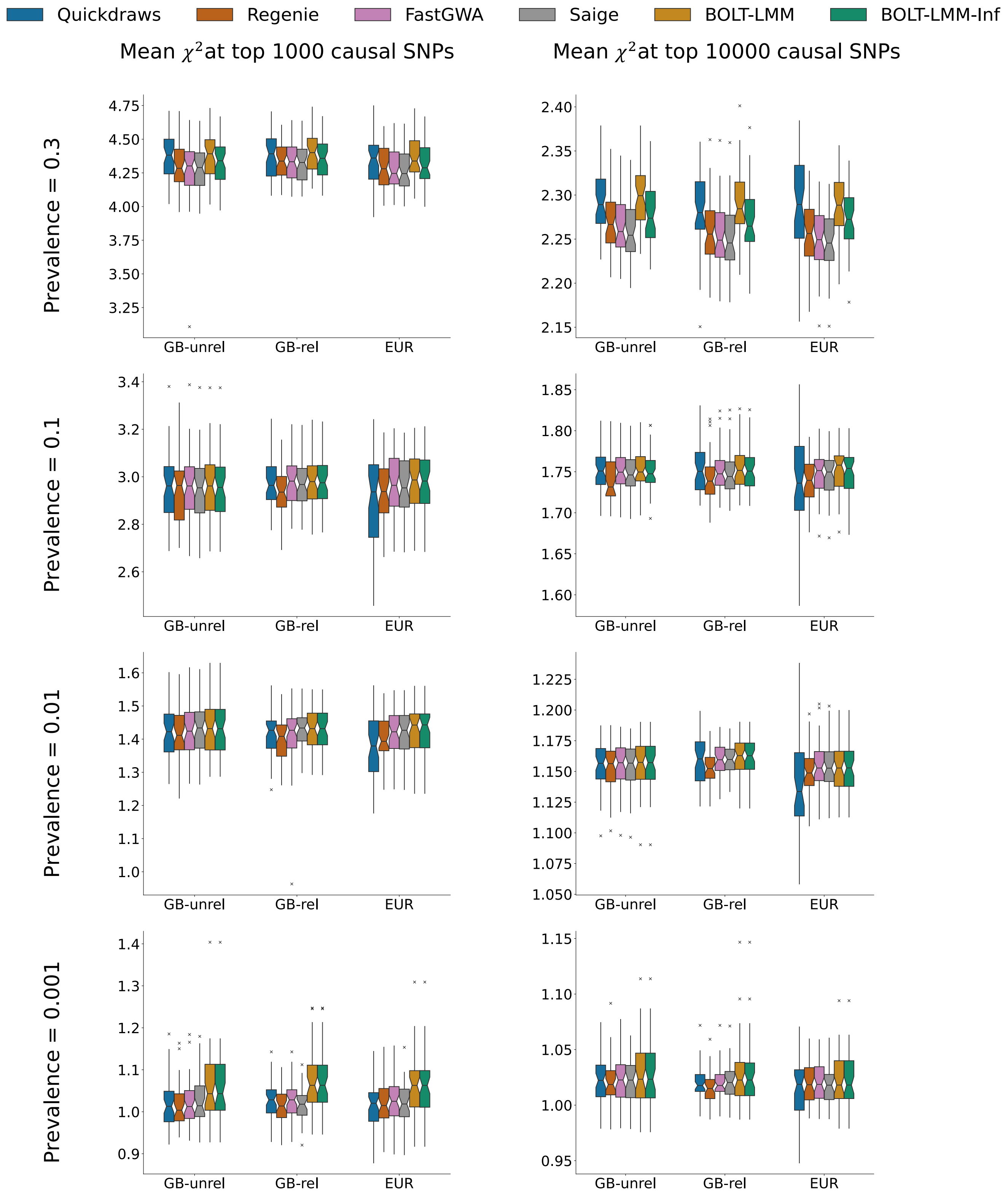

Figure 2: **Summary of statistical power in simulations for binary traits.** We measure the normalized causal χ^2 at top 1,000 and top 10,000 causal variants. To correct for confounding, the causal χ^2 is normalized by the average χ^2 at null variants on even chromosomes. The line inside each box indicates the median value, the central box indicates the interquartile range, whiskers indicate data up to 1.5 times the IQR, and outliers are shown as separate points. These results are based on simulations of 50 independent traits. A description of the group labels (GB-unrel, GB-rel, EUR) is provided in the Methods section.

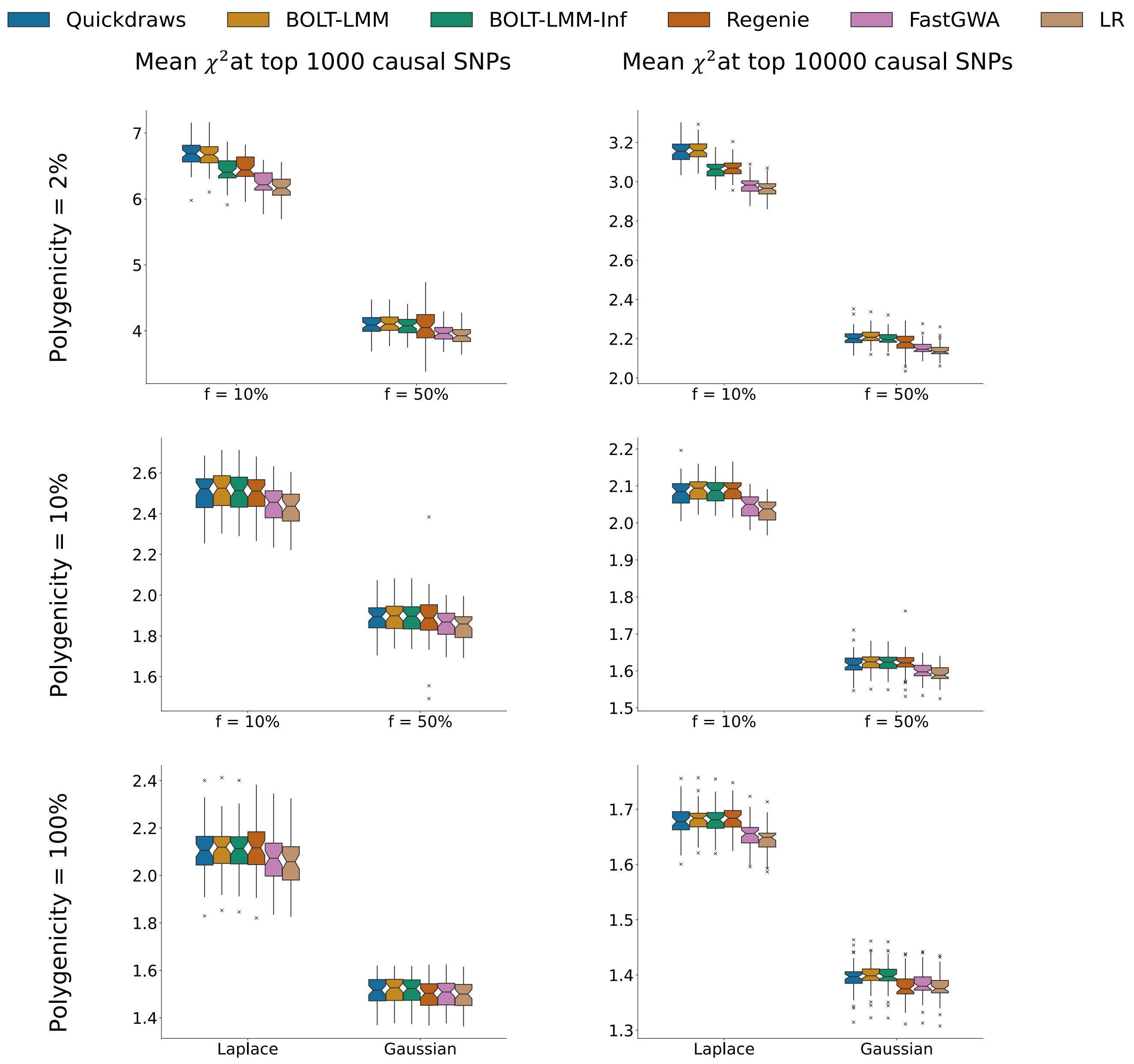

Figure 3: **Summary of statistical power in simulations with varying causal effect distributions.** The first two rows correspond to causal effects simulated from a mixture of two Gaussian. Polygenicity refers the proportion corresponding to the Gaussian with higher variance, and f refers to the fraction of total variance explained by the Gaussian with lower variance. We measure the normalized causal χ^2 at top 1,000 and top 10,000 causal variants. To correct for confounding, the causal χ^2 is normalized by the average χ^2 at null variants on even chromosomes. The line inside each box indicates the median value, the central box indicates the interquartile range (IQR), whiskers indicate data up to 1.5 times the IQR, and outliers are shown as separate points. These results are based on simulations of 50 independent traits.

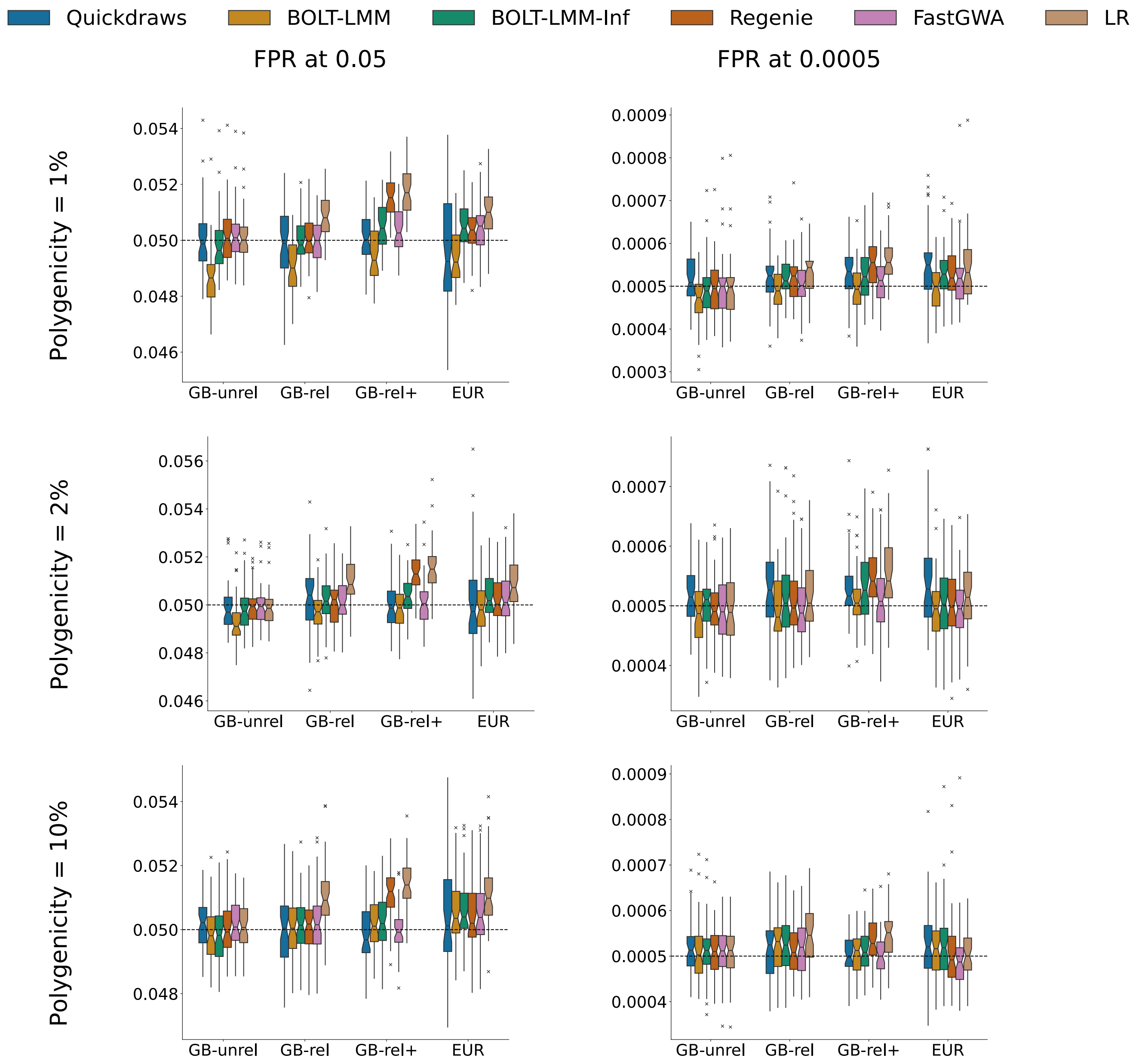

Figure 4: **Summary of calibration in simulations for quantitative traits.** False positive rate (FPR) at a significance threshold of $\alpha \in \{0.05, 0.0005\}$, calculated as the fraction of variants on even chromosomes with p-value lower than α. The line inside each box indicates the median value, the central box indicates the interquartile range, whiskers indicate data up to 1.5 times the IQR, and outliers are shown as separate points. These results are based on simulations of 50 independent traits. A description of the group labels (GB-unrel, GB-rel, GB-rel+, EUR) is provided in the Methods section. Inflation was assessed using Bonferroni-corrected two-sided t-tests, with numerical values available in Supplementary Table 1.

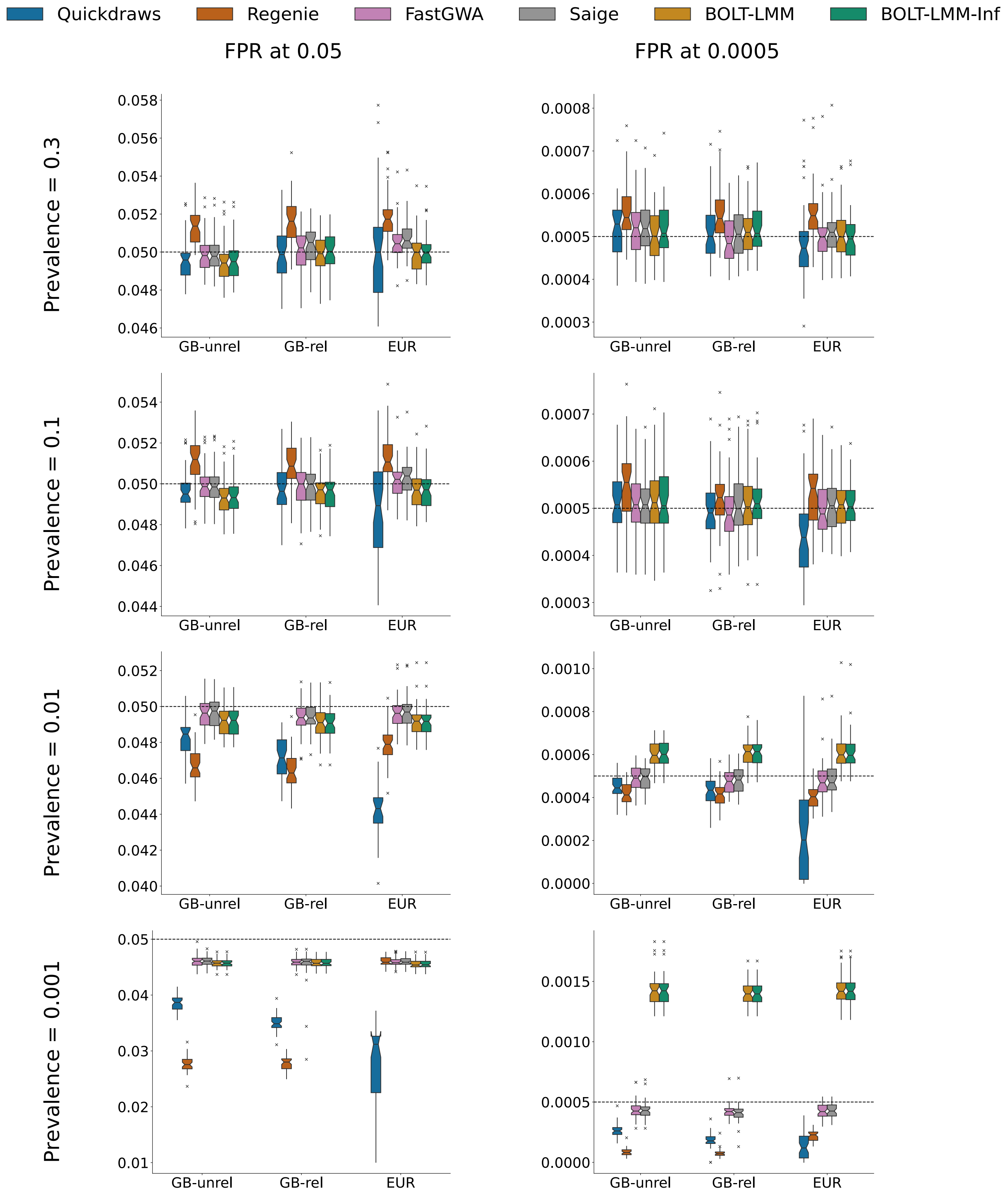

Figure 5: **Summary of calibration in simulations for binary traits and common variants (MAF $\geq$ 1%):** False positive rate (FPR) at a significance threshold of $\alpha \in \{0.05, 0.0005\}$, calculated as the fraction of variants on even chromosomes with p-value lower than α. The line inside each box indicates the median value, the central box indicates the interquartile range, whiskers indicate data up to 1.5 times the IQR, and outliers are shown as separate points. These results are based on simulations of 50 independent traits. A description of the group labels (GB-unrel, GB-rel, EUR) is provided in the Methods section. Inflation was assessed using Bonferroni-corrected two-sided t-tests, with numerical values available in Supplementary Table 1.

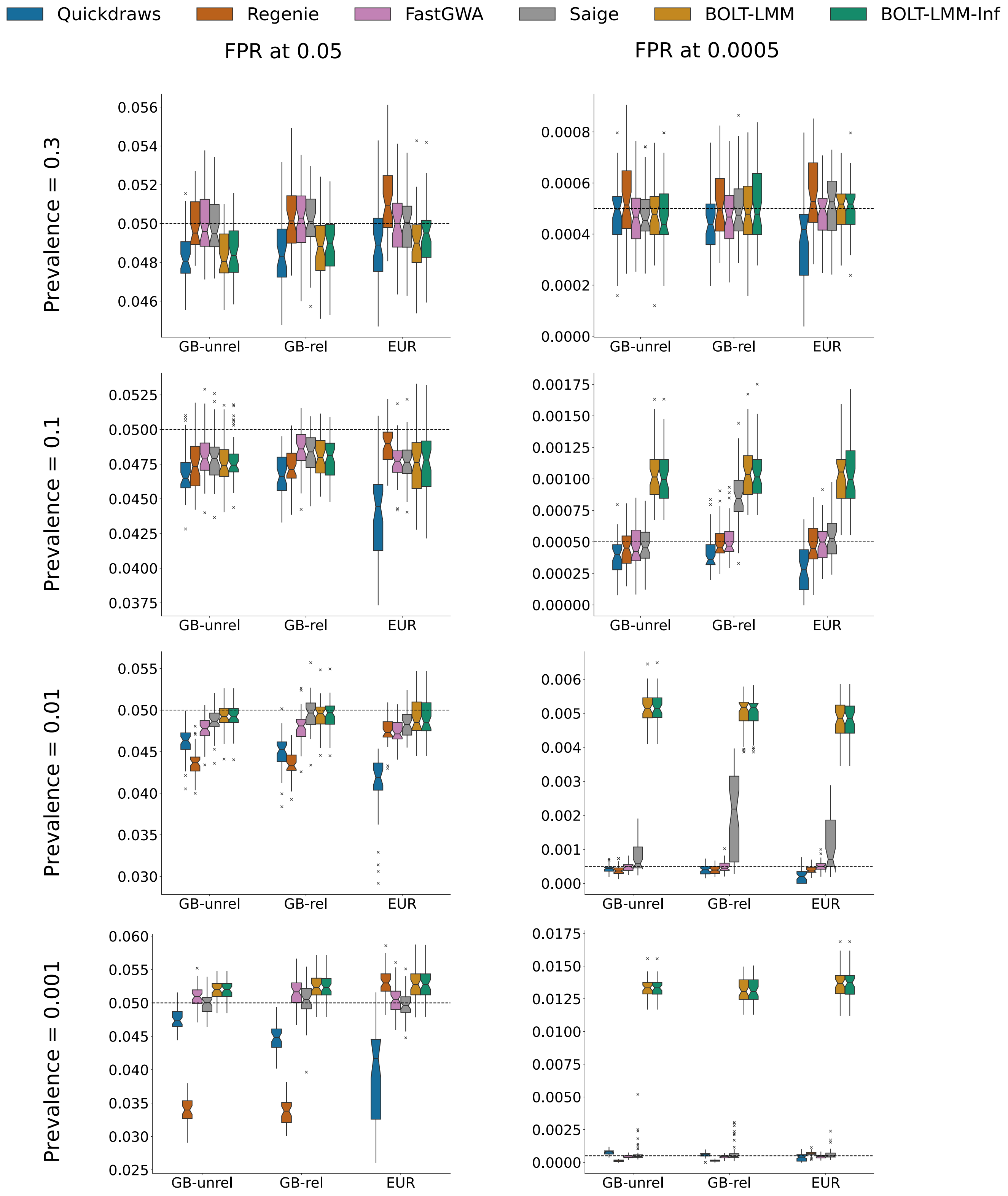

Figure 6: **Summary of calibration in simulations for binary traits and rare variants (MAF $< 1\%$):** False positive rate (FPR) at a significance threshold of $\alpha \in \{0.05, 0.0005\}$, calculated as the fraction of variants on even chromosomes with p-value lower than α. The line inside each box indicates the median value, the central box indicates the interquartile range, whiskers indicate data up to 1.5 times the IQR, and outliers are shown as separate points. These results are based on simulations of 50 independent traits. A description of the group labels (GB-unrel, GB-rel, EUR) is provided in the Methods section. Inflation was assessed using Bonferroni-corrected two-sided t-tests, with numerical values available in Supplementary Table 1.

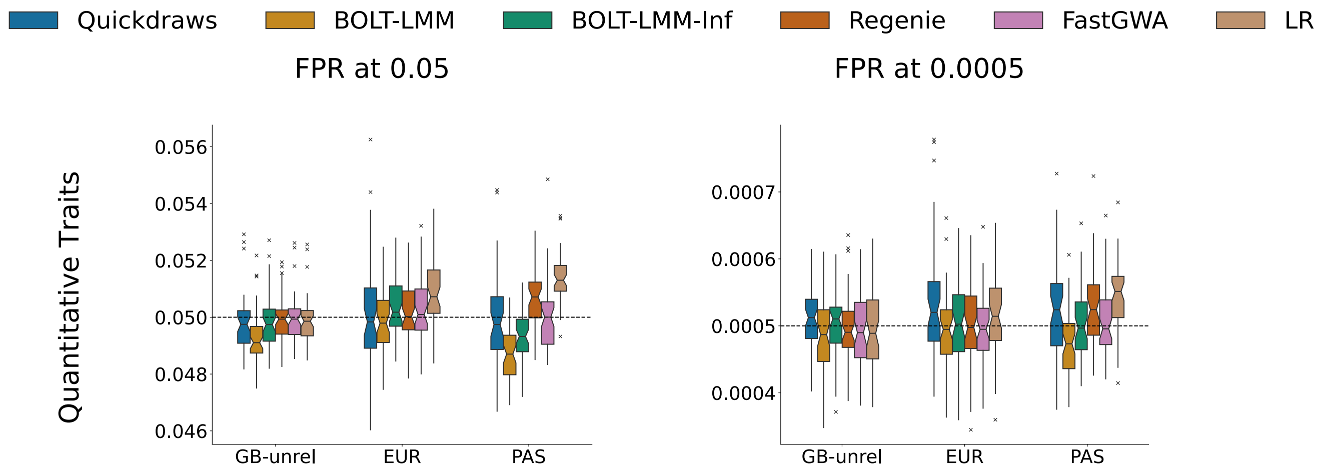

Figure 7: **Summary of calibration in simulations with varying levels of population structure.** False positive rate (FPR) at a significance threshold of $\alpha \in \{0.05, 0.0005\}$, calculated as the fraction of variants on even chromosomes with p-value lower than α. The line inside each box indicates the median value, the central box indicates the interquartile range, whiskers indicate data up to 1.5 times the IQR, and outliers are shown as separate points. These results are based on simulations of 50 independent traits. A description of the group labels (GB-unrel, EUR, PAS) is provided in the Methods section. Inflation was assessed using Bonferroni-corrected two-sided t-tests, with numerical values available in Supplementary Table 1.

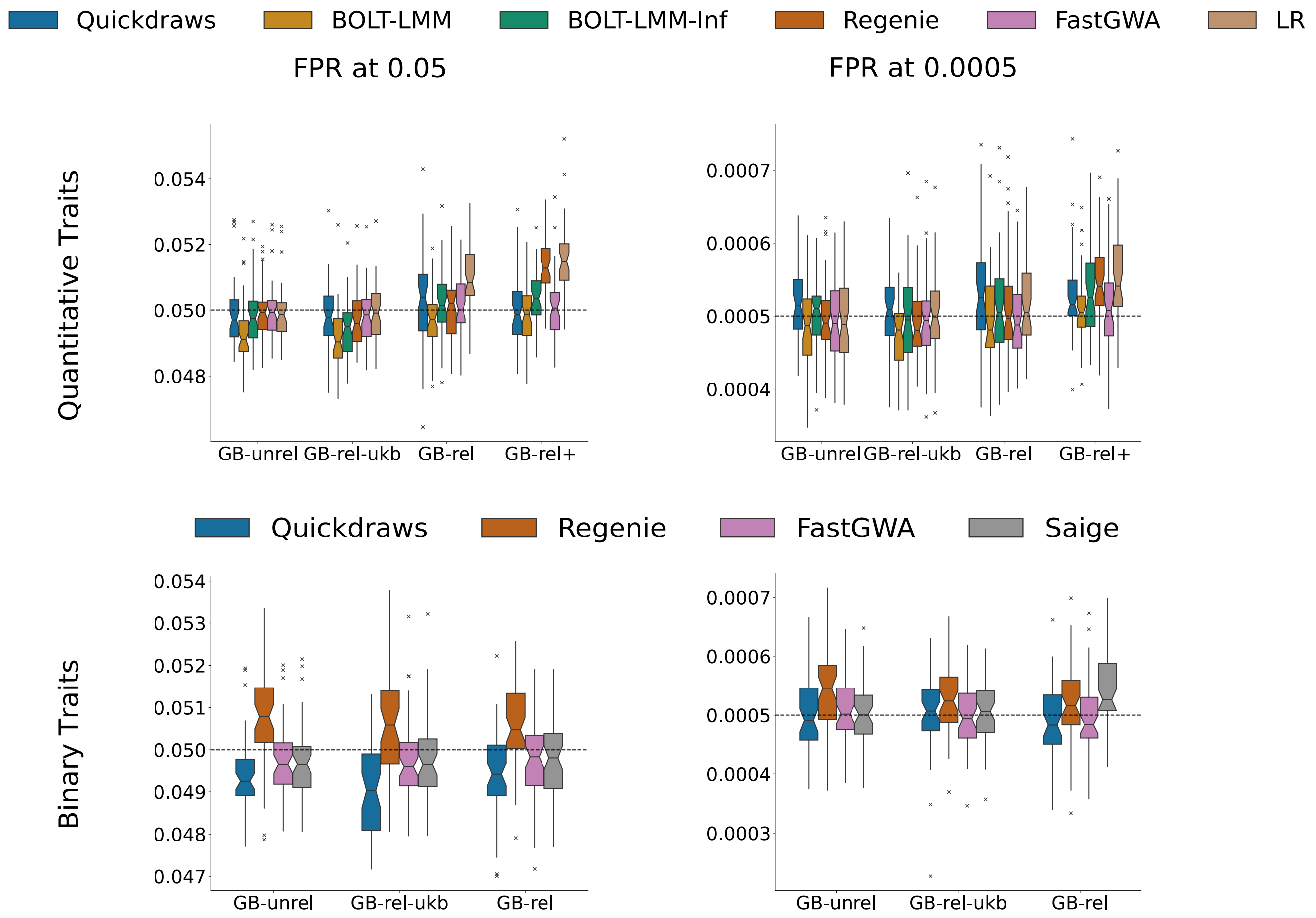

Figure 8: **Summary of calibration in simulations with varying levels of relatedness.** False positive rate (FPR) at a significance threshold of $\alpha \in \{0.05, 0.0005\}$, calculated as the fraction of variants on even chromosomes with p-value lower than α. The line inside each box indicates the median value, the central box indicates the interquartile range, whiskers indicate data up to 1.5 times the IQR, and outliers are shown as separate points. GB-unrel refers to simulations including only unrelated British individuals, GB-rel-ukb refers to randomly sampling from the related white British subset, GB-rel refers to the default relatedness setting with 3.4× more relative pairs compared to the UK Biobank, and GB-rel+ refers to the extreme relatedness case of 7.3× and 4.8× more first and second degree relative pairs compared to the UK Biobank. The prevalence of binary traits was fixed at 10%. These results are based on simulations of 50 independent traits. Inflation was assessed using Bonferroni-corrected two-sided t-tests, with numerical values available in Supplementary Table 1.

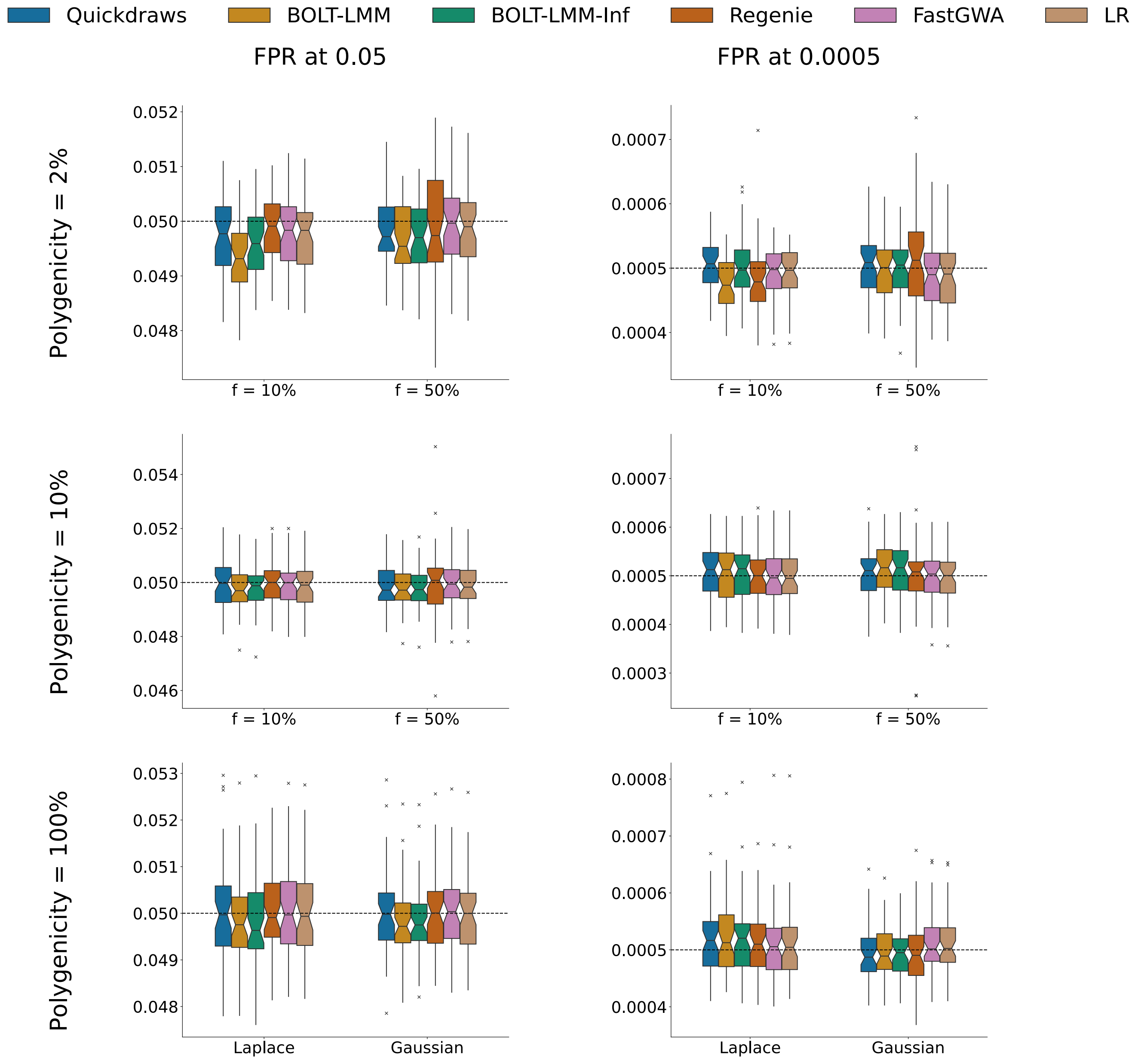

Figure 9: **Summary of calibration in simulations with varying causal effect distributions:** The first two rows correspond to causal effects simulated from a mixture of two Gaussian. Polygenicity refers to the proportion corresponding to the Gaussian with higher variance, and f refers to the fraction of total variance explained by the Gaussian with lower variance. False positive rate (FPR) at a significance threshold of $\alpha \in \{0.05, 0.0005\}$, calculated as the fraction of variants on even chromosomes with p-value lower than α. The line inside each box indicates the median value, the central box indicates the interquartile (IQR) range, whiskers indicate data up to 1.5 times the IQR, and outliers are shown as separate points. These results are based on simulations of 50 independent traits. Inflation was assessed using Bonferroni-corrected two-sided t-tests, with numerical values available in Supplementary Table 1.

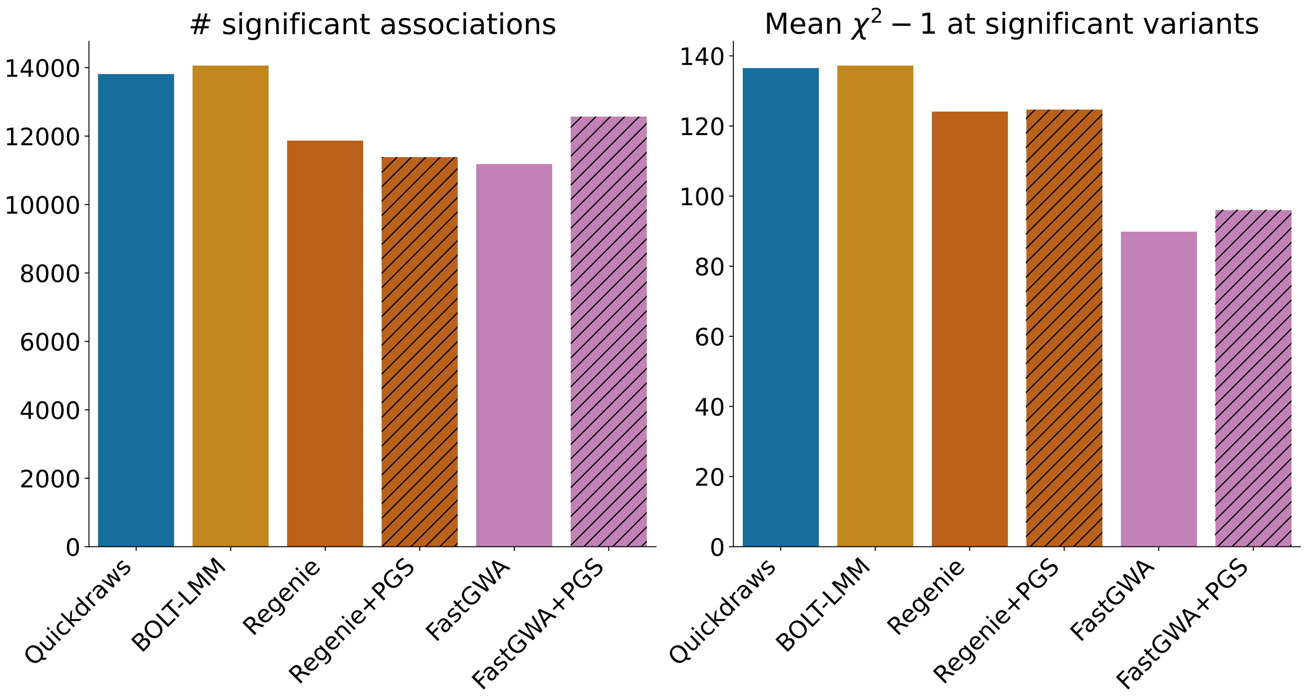

Figure 10: **Effects of PGS-adjustment on association power.** (a) Total number of indepedent loci found on chromosomes 1-5, using different GWAS methods with and without PGS adjustment in 79 UK Biobank quantitative traits. (b) Mean χ^2 - 1 at significant variants with and without PGS adjustment for the same traits and regions. Significant variants were defined as those with genome-wide significant ($P = 5 \times 10^{-8}$) LR-unrel GWAS p-value. FastGWA with PGS adjustment yielded an increase in the number of independent loci (with PGS adjustment, 159.1; without, 141.6; paired two-sided t-test $p = 3.8 \times 10^{-9}$) and higher mean χ^2-1 at significant variants (with PGS adjustment, 96.0; without, 89.8; paired two-sided t-test $p = 6.6 \times 10^{-11}$). Regenie with PGS adjustment yielded fewer independent loci (with PGS adjustment, 144.1; without, 150.1; paired two-sided t-test $p = 1.5 \times 10^{-5}$), and similar mean χ^2 - 1 values (with PGS adjustment, 124.6; without, 124.1; paired two-sided t-test $p > 0.05$).

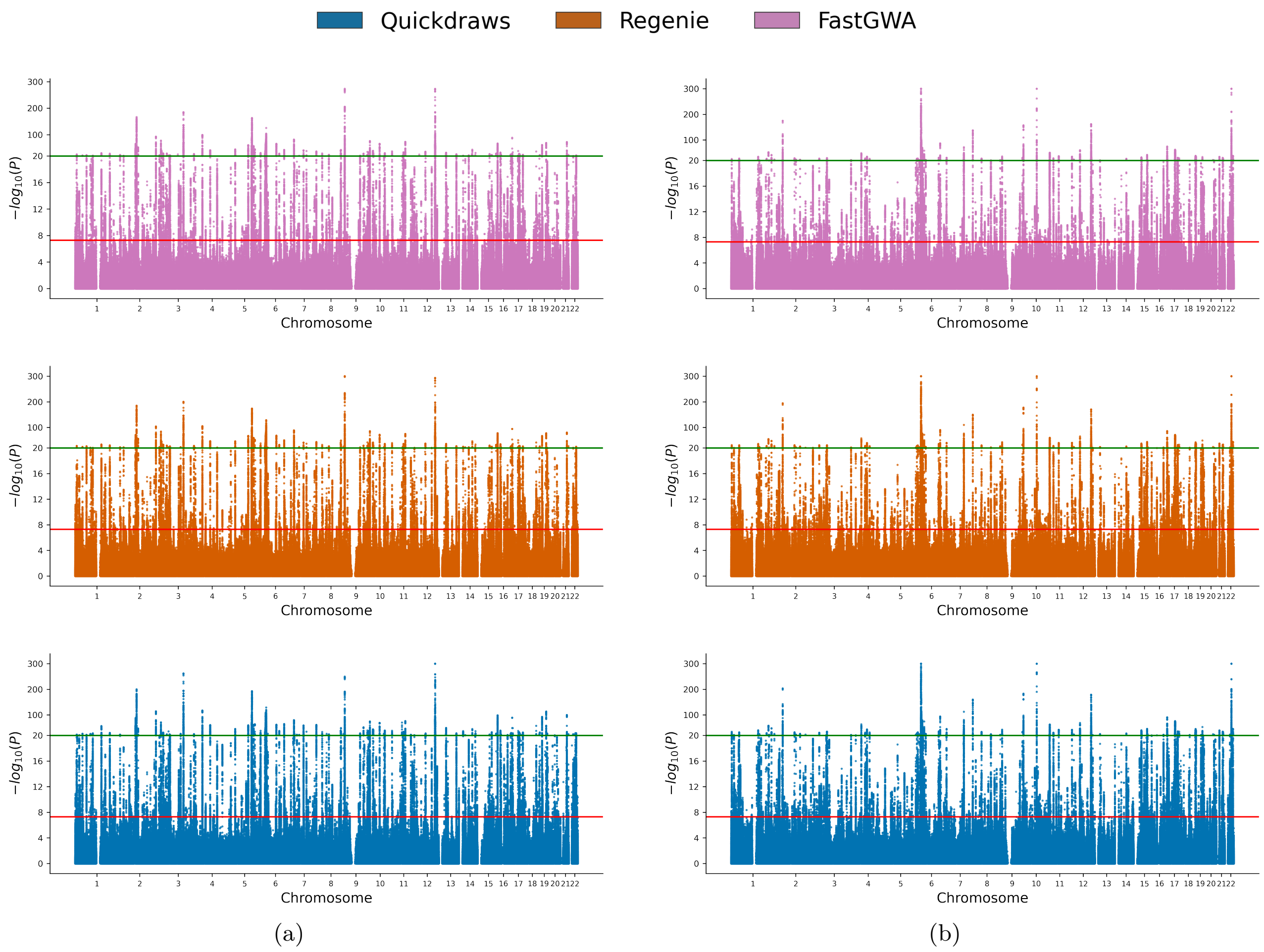

Figure 11: **Manhattan plots for two quantitative traits.** (a) Eosinophil count (RHE-me $h^2 = 0.213$) and (b) Haemoglobin concentration (RHE-mc $h^2 = 0.158$). ∼13.3 million variants were tested. Red lines indicate a genome-wide significance threshold of $P = 5 \times 10^{-8}$ and the green line indicates $P = 1 \times 10^{-20}$. P-values from different GWAS methods are log-transformed, with $P = 1 \times 10^{-20}$ marking the change in y-axis scaling.

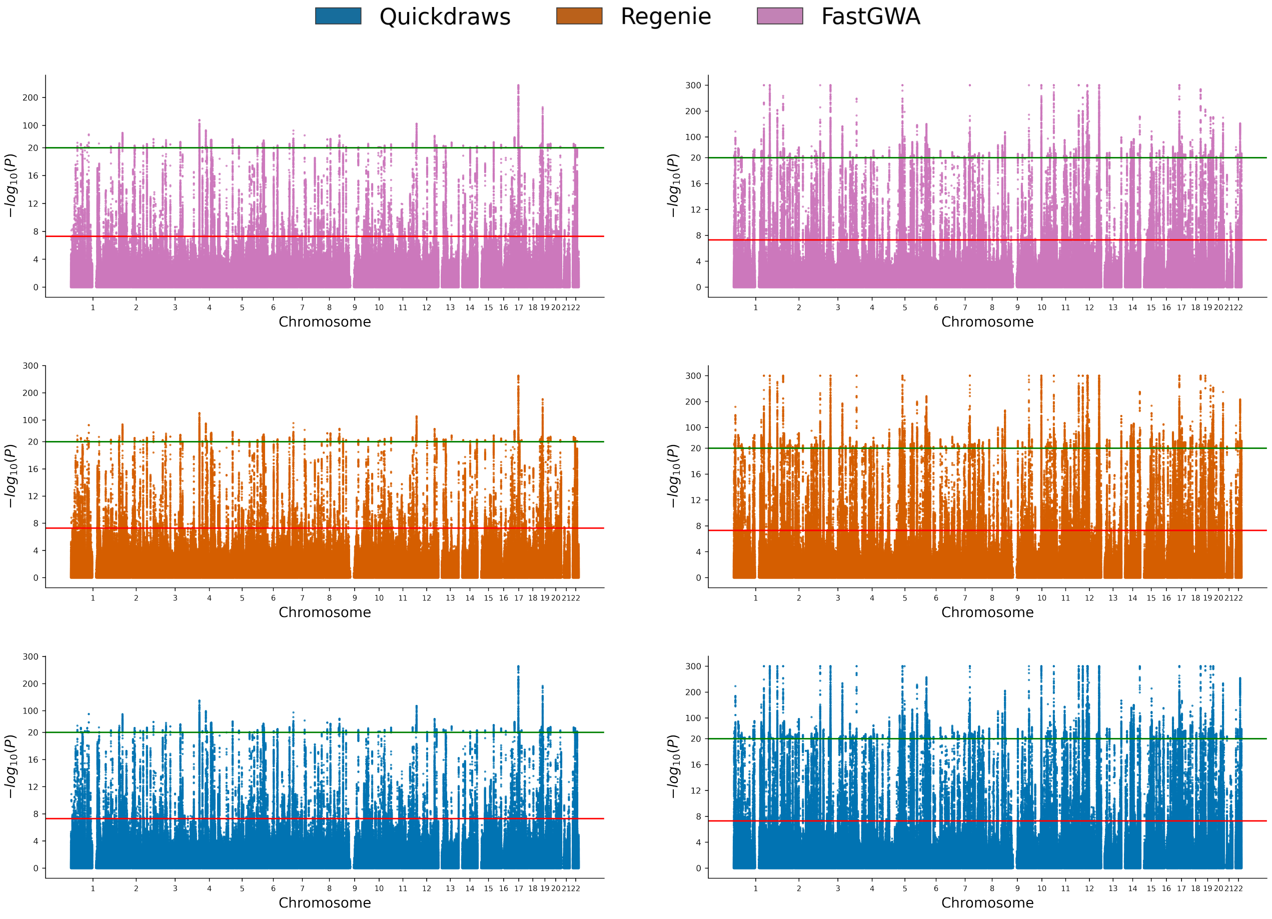

(a) (b)

Figure 12: **Manhattan plots for two quantitative traits.** (a) Lymphocyte percentage (RHE-mc $h^2 = 0.196$) and (b) Mean platelet volume (RHE-mc $h^2 = 0.448$). $\sim$13.3 million variants were tested. Red lines indicate a genome-wide significance threshold of $P = 5 \times 10^{-8}$ and the green line indicates $P = 1 \times 10^{-20}$. P-values from different GWAS methods are log-transformed, with $P = 1 \times 10^{-20}$ marking the change in y-axis scaling.

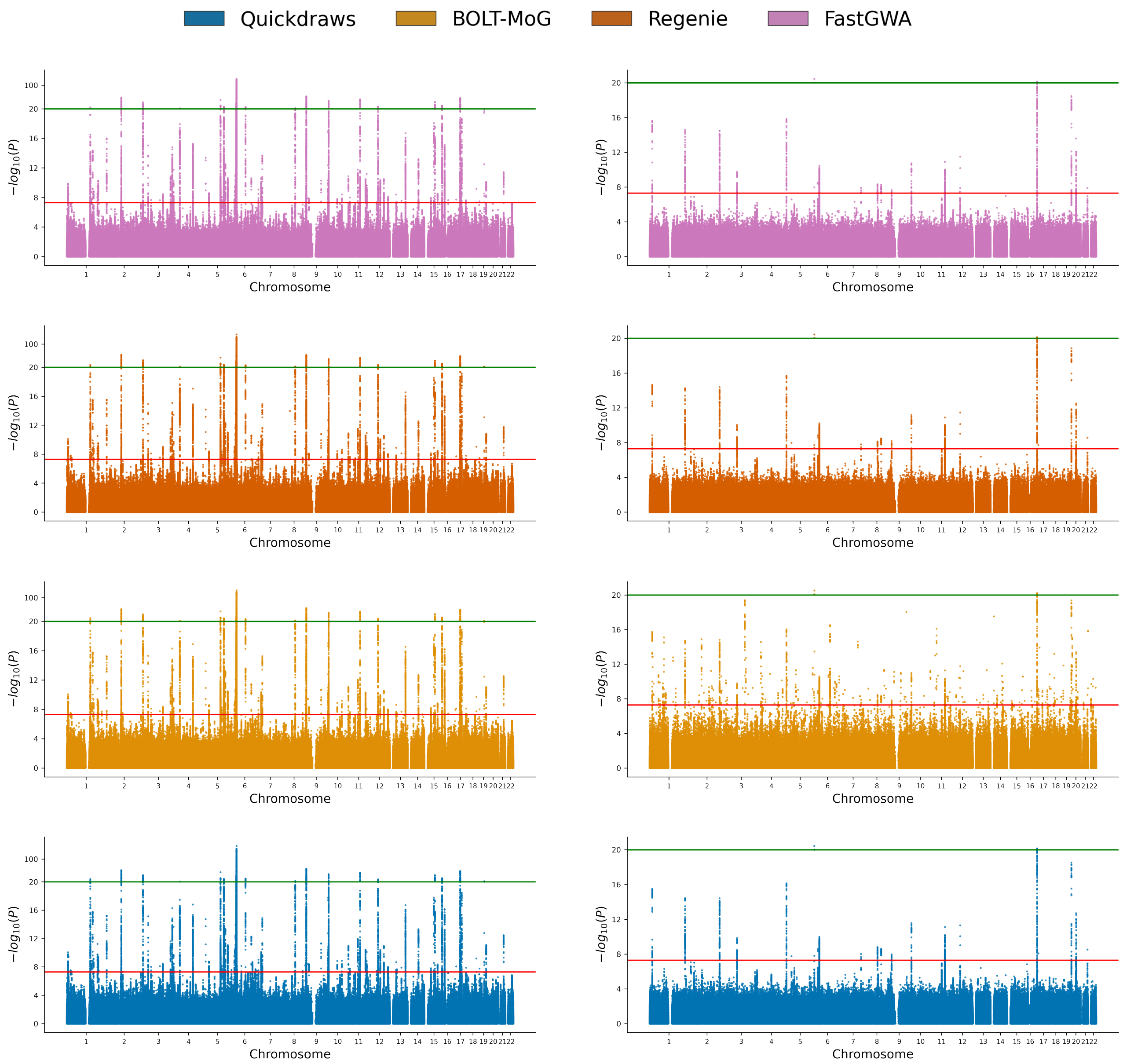

(a) (b)

Figure 13: **Manhattan plots for two binary traits.** (a) Asthma (prevalence = 0.119) and (b) Basal cell carcinoma (prevalence = 0.012). ~13.3 million variants were tested. Red lines indicate a genome-wide significance threshold of $P = 5 \times 10^{-8}$ and the green line indicates $P = 1 \times 10^{-20}$. P-values from different GWAS methods are log-transformed, with $P = 1 \times 10^{-20}$ marking the change in y-axis scaling.

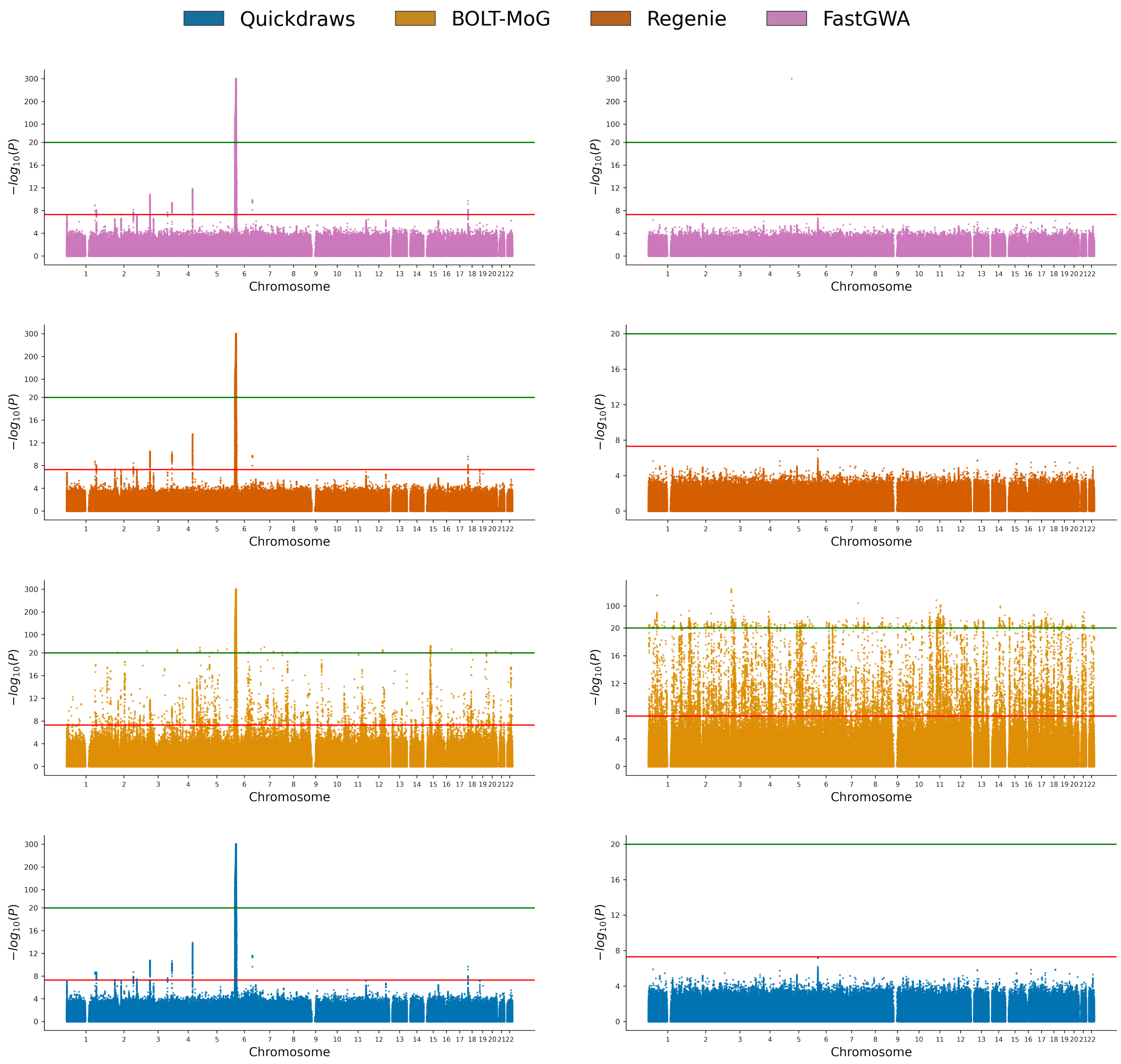

(a) (b)

Figure 14: **Manhattan plots for two binary traits.** (a) Celiac disease (prevalence = 0.0047) and (b) Vitiligo (prevalence = 0.0005). ∼13.3 million variants were tested. Red lines indicate a genome-wide significance threshold of $P = 5 \times 10^{-8}$ and the green line indicates $P = 1 \times 10^{-20}$. P-values from different GWAS methods are log-transformed, with $P = 1 \times 10^{-20}$ marking the change in y-axis scaling.

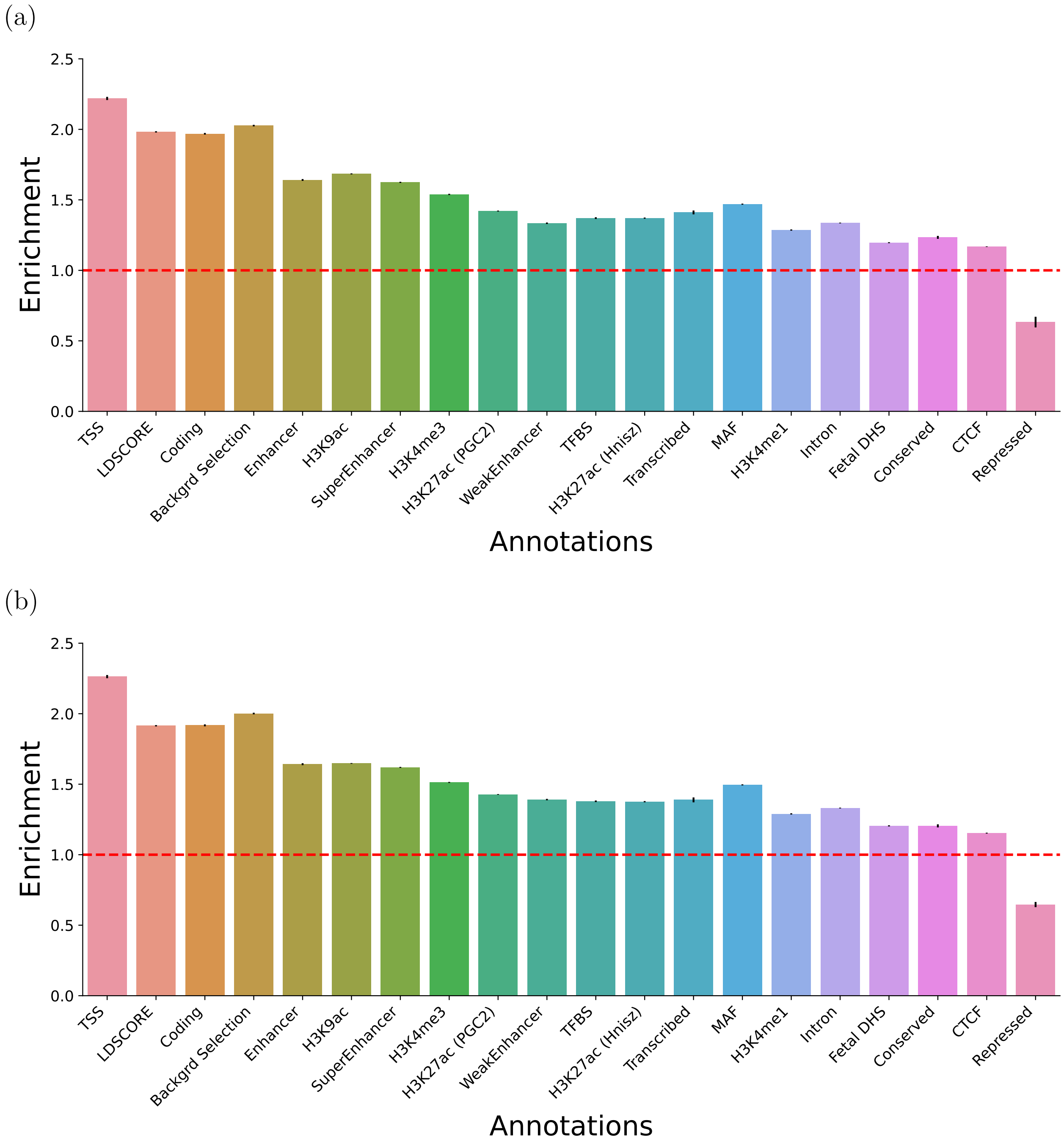

Figure 15: **Functional enrichment at associated variants for quantitative traits.** Functional enrichment profile of variants associated across 79 quantitative traits. We considered either (a) the set of variants significantly associated at genome-wide significance (GWAS $p = 5 \times 10^{-8}$) using both Quickdraws and Regenie, matching the p-value distribution (see Methods), or (b) the set of variants associated using Quickdraws but not Regenie. Error bars are presented as mean ± jackknife standard errors; the red dashed line corresponds to no enrichment.

(a)

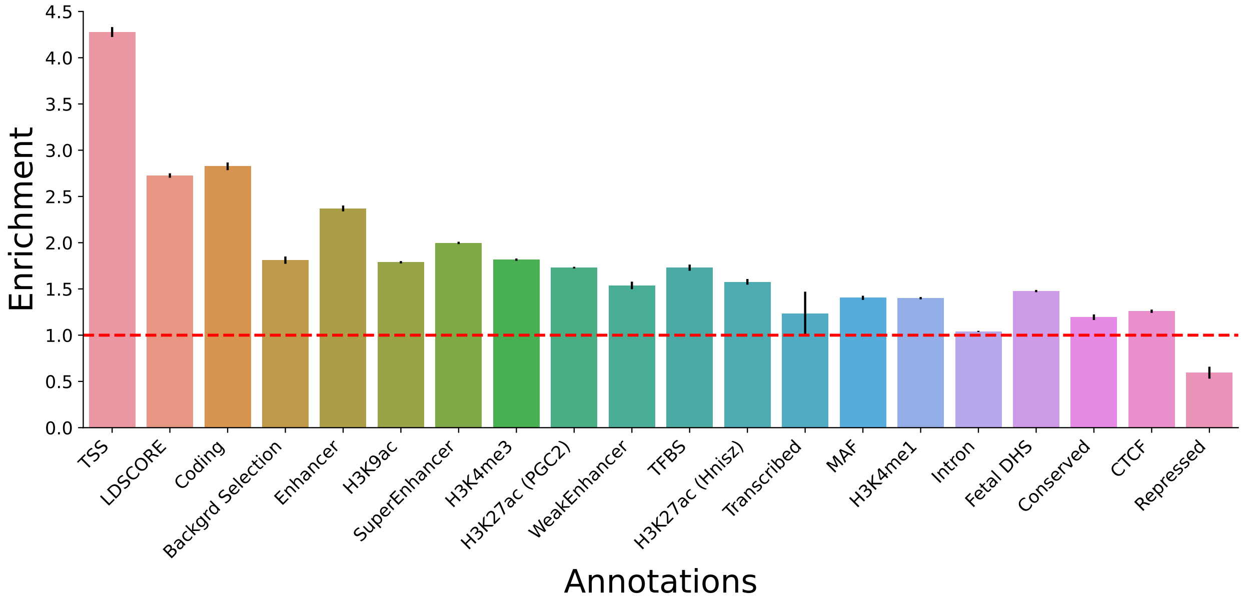

(b)

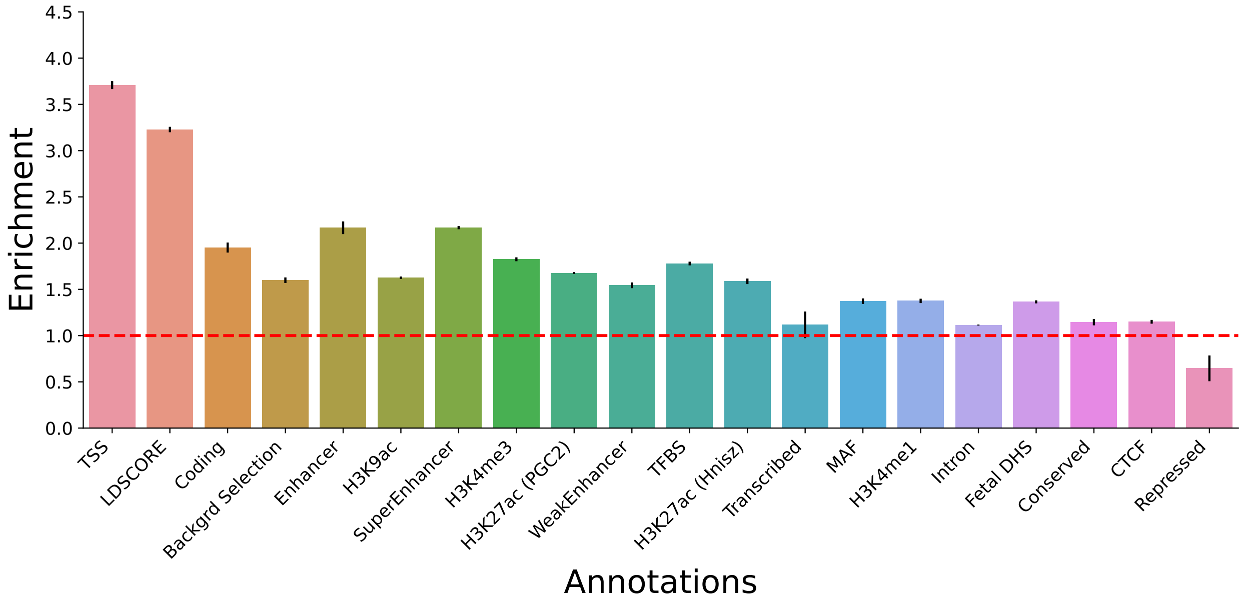

Figure 16: **Functional enrichment at associated variants for binary traits.** Functional enrichment profile of variants associated across 50 binary traits. We considered either (a) the set of variants significantly associated at genome-wide significance (GWAS $p = 5 \times 10^{-8}$) using both Quickdraws and Regenie, matching the p-value distribution (see Methods), or (b) the set of variants associated using Quickdraws but not Regenie. Error bars are presented as mean ± jackknife standard errors; the red dashed line corresponds to no enrichment.

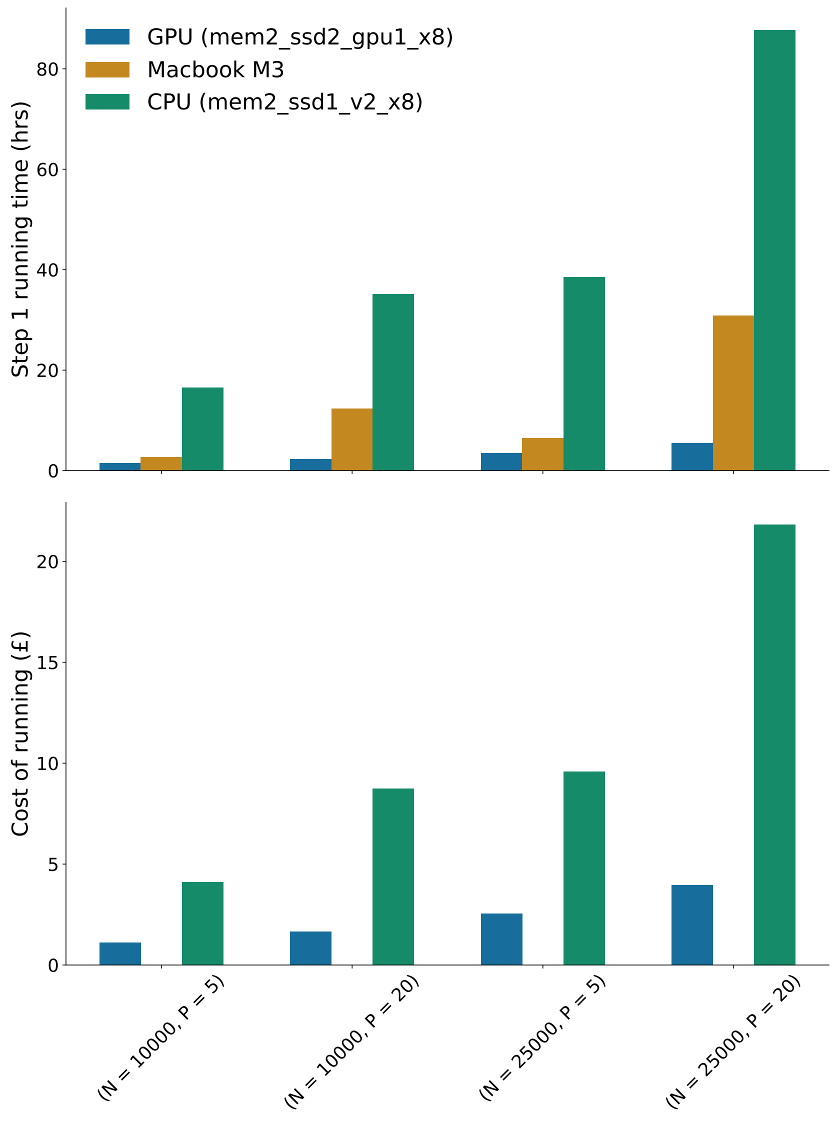

Figure 17: **Computational performance of Quickdraws on different computing architectures.** We assessed the running time and cost associated with Quickdraws' model fitting step (Step 1) across varying numbers of samples (N) and phenotypes (P). Performance was compared for three architectures: an Nvidia A10G GPU node on the UK Biobank Research Analysis Platform (RAP) (`mem2_ssd2_gpu1_x8`), a 14-core MacBook Pro equipped with the Apple M3 chip, and an 8-core CPU node on the UK Biobank RAP (`mem2_ssd1_v2_x8`). Running costs were calculated only for the GPU and CPU nodes on the RAP.

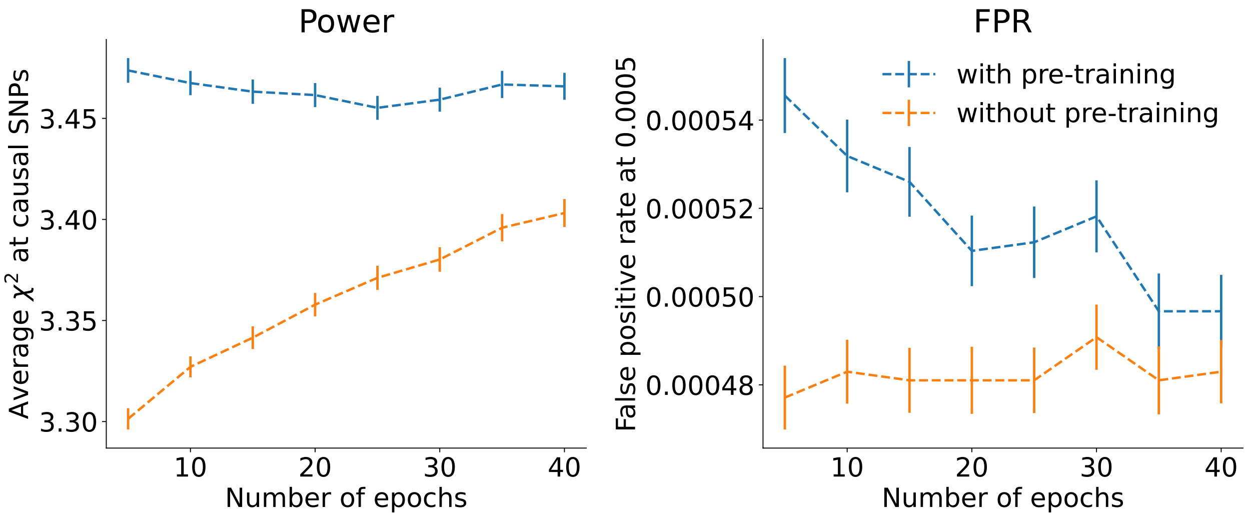

Figure 18: **Transfer-learning in Bayesian regression.** We measure the causal χ^2 and false positive rate at 5×10^{-4}, with and without pre-training (for 90 epochs), and varying the number of LOCO training epochs. Error bars represent the standard error of the mean χ^2 or false positive rate across 50 independent quantitative traits with GB-unrel structure and 2% polygenicity.

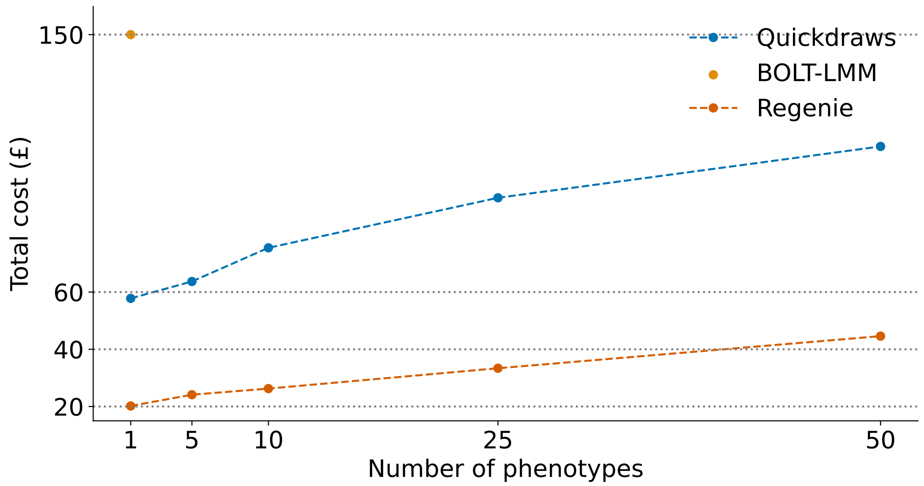

Figure 19: **Cost of running on UK Biobank RAP for Quickdraws compared with Regenie and BOLT-LMM, with increasing number of quantitative phenotypes.** Regenie and Quickdraws scale sub-linearly as they support parallelization across traits, whereas BOLT-LMM runs one phenotype at a time. The cost is reported for ∼13.3 million tests with N = 405,088 samples and M = 458,260 variants for model fitting on the mem1_ssd1_v2_x36 RAP instance.

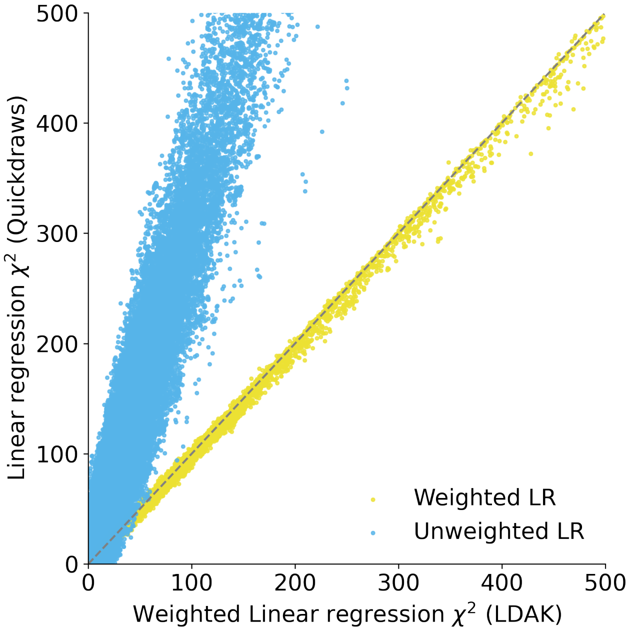

Figure 20: **Weighted linear regression in Quickdraws and LDAK.** The χ^2 statistics generated using weighted linear regression in LDAK approximately match those of a weighted linear regression implemented in Quickdraws ($R^2 = 99.2\%$) and differ from those from unweighted linear regression ($R^2 = 81.2\%$) on the same samples. Both methods were applied to the unrelated white British UK Biobank subgroup ($N = 337k$) and 79 quantitative traits. Axes are clipped at $\chi^2 = 500$.
