# Supplementary material for: A scalable variational inference approach for increased mixed-model association power
Source: Nat Genet. 2025 Jan 9;57(2):461–8. doi: 10.1038/s41588-024-02044-7 (PMC11821521; doi:10.1038/s41588-024-02044-7)
Supplement: Supplementary file 2 — Reporting Summary [file 41588_2024_2044_MOESM2_ESM.pdf]

nature portfolio

Corresponding author(s): Pier Palamara
Hrushikesh Loya

Last updated by author(s): Oct 13, 2024

Reporting Summary

Nature Portfolio wishes to improve the reproducibility of the work that we publish. This form provides structure for consistency and transparency in reporting. For further information on Nature Portfolio policies, see our Editorial Policies and the Editorial Policy Checklist.

Statistics

For all statistical analyses, confirm that the following items are present in the figure legend, table legend, main text, or Methods section.

n/a	Confirmed	
☐	☒	The exact sample size (n) for each experimental group/condition, given as a discrete number and unit of measurement
☐	☒	A statement on whether measurements were taken from distinct samples or whether the same sample was measured repeatedly
☐	☒	The statistical test(s) used AND whether they are one- or two-sided<br>*Only common tests should be described solely by name; describe more complex techniques in the Methods section.*
☐	☒	A description of all covariates tested
☐	☒	A description of any assumptions or corrections, such as tests of normality and adjustment for multiple comparisons
☐	☒	A full description of the statistical parameters including central tendency (e.g. means) or other basic estimates (e.g. regression coefficient) AND variation (e.g. standard deviation) or associated estimates of uncertainty (e.g. confidence intervals)
☐	☒	For null hypothesis testing, the test statistic (e.g. F, t, r) with confidence intervals, effect sizes, degrees of freedom and P value noted<br>*Give P values as exact values whenever suitable.*
☐	☒	For Bayesian analysis, information on the choice of priors and Markov chain Monte Carlo settings
☐	☒	For hierarchical and complex designs, identification of the appropriate level for tests and full reporting of outcomes
☐	☒	Estimates of effect sizes (e.g. Cohen's d, Pearson's r), indicating how they were calculated

Our web collection on statistics for biologists contains articles on many of the points above.

Software and code

Policy information about availability of computer code

Data collection: No software was used for data collection

Data analysis:
Quickdraws (v0.1.0) https://palamaralab.github.io/software/quickdraws
Quickdraws analysis scripts https://doi.org/10.5281/zenodo.13936055
Regenie (v3.1.1) https://rgcgithub.github.io/regenie/
PLINK (v1.9) https://www.cog-genomics.org/plink2/
BOLT-LMM (v2.4.1) https://alkesgroup.broadinstitute.org/BOLT-LMM
FastGWA (v1.94.1) https://yanglab.westlake.edu.cn/software/gcta/#Overview
FastGWA-GLMM (v1.94.1) https://yanglab.westlake.edu.cn/software/gcta/#Overview
Saige (v1.1.6) https://saigegit.github.io/SAIGE-doc/
RHE (PyPI version 1.0.0) https://pypi.org/project/rhe/
LDSC software (v1.0.0) https://github.com/bulik/ldsc
LDAK software (v5.2) http://dougspeed.com/downloads2/
Python package PySnpTools (v0.5.10) https://github.com/fastlmm/PySnpTools
Python package PyTorch (v2.4.0) https://pytorch.org/
Python package Numba (v0.55.1) https://numba.pydata.org/
Python package Numpy (v2.1.2) https://numpy.org/

For manuscripts utilizing custom algorithms or software that are central to the research but not yet described in published literature, software must be made available to editors and reviewers. We strongly encourage code deposition in a community repository (e.g. GitHub). See the Nature Portfolio guidelines for submitting code & software for further information.

Data

Policy information about availability of data

All manuscripts must include a data availability statement. This statement should provide the following information, where applicable:

- Accession codes, unique identifiers, or web links for publicly available datasets
- A description of any restrictions on data availability
- For clinical datasets or third party data, please ensure that the statement adheres to our policy

Summary statistics generated using Quickdraws for quantitative, binary, and plasma protein traits are available at https://www.stats.ox.ac.uk/publication-data/sge/ppg/quickdraws/. The individual-level genotype and phenotype data are available to approved researchers through the UK Biobank (http://www.ukbiobank.ac.uk). Summary association statistics for Biobank Japan were downloaded from https://pheweb.jp/. Summary association statistics for FinnGen were obtained, following approval, through https://www.finngen.fi/en/access_results. Baseline-LD annotations and HapMap3 filtered European LD scores for comparing attenuation ratios were downloaded from https://alkesgroup.broadinstitute.org/LDSCORE/.

Research involving human participants, their data, or biological material

Policy information about studies with human participants or human data. See also policy information about sex, gender (identity/presentation), and sexual orientation and race, ethnicity and racism.

Reporting on sex and gender	Sex was not considered in simulation experiments. In UK Biobank analyses, individuals of all sexes were included and only autosomal regions were analyzed. Sex, sex*age, and sex*age^2 were included as covariates.
Reporting on race, ethnicity, or other socially relevant groupings	We analyzed subsets of the UK Biobank dataset by grouping individuals using genetic data and self-reported ethnicity, relying on groups previously specified in Bycroft et al. 2018. We used the top 20 genetic principal components as covariates in several analyses.
Population characteristics	We included information on age, sex, genotype, smoking status, and 20 genetic principal components as covariates in several analyses. Genotype data was obtained from the UK Biobank.
Recruitment	We did not recruit participants for this study.
Ethics oversight	UK Biobank data were analyzed after approval of UK Biobank proposal no. 43206.

Note that full information on the approval of the study protocol must also be provided in the manuscript.

Field-specific reporting

Please select the one below that is the best fit for your research. If you are not sure, read the appropriate sections before making your selection.

☒ Life sciences ☐ Behavioural & social sciences ☐ Ecological, evolutionary & environmental sciences

For a reference copy of the document with all sections, see nature.com/documents/nr-reporting-summary-flat.pdf

Life sciences study design

All studies must disclose on these points even when the disclosure is negative.

Sample size	For real data analysis, we used 405,088 related white British individuals (as reported in Bycroft et al. Nature 2018) from the UK Biobank. For simulations, we used various subsets of self-reported European individuals in the UK Biobank (N = 462,428) to assess robustness and statistical power to detect associations across several population structure and relatedness settings. The sample sizes in the simulations ranged from 50,000 to the entire European subset of the UK Biobank (N = 462,428), with most simulations performed on 50,000 samples. This sample size was chosen to optimize the trade-off between computational scalability for multiple experiments and statistical power for assessing inflation and statistical power.
Data exclusions	For model fitting in GWAS, we considered the set of available autosomal variants to have minor allele frequency > 1%, Hardy-Weinberg equilibrium p > 1e-15, and a genotyping rate above 99%, obtaining a set of 458,620 markers. For the calculation of test statistics, we considered 13.3 million imputed variants, filtering based on MAF (MAF > 0.1%) and INFO score (INFO > 0.8). We grouped samples using genetic principal components, self-reported ethnicity, and genetic relatedness.
Replication	We performed replication analysis of associations found in 405,088 UK Biobank samples, using summary statistics from Biobank Japan, FinnGen, and other large-scale studies.
Randomization	Participants were not allocated into experimental groups for this study. In conducting phenotypic association analyses, we adjusted for covariates by initially stratifying by sex and applying quantile normalization. We then regressed out the top 20 principal components, age, sex, age^2, age*sex, age^2*sex, and smoking status.
Blinding	Blinding was not applicable to the analyses we performed within the UK Biobank, which analyzed all samples jointly. Data collection was performed previously by the UK Biobank.

Reporting for specific materials, systems and methods

We require information from authors about some types of materials, experimental systems and methods used in many studies. Here, indicate whether each material, system or method listed is relevant to your study. If you are not sure if a list item applies to your research, read the appropriate section before selecting a response.

Materials & experimental systems

n/a	Involved in the study
☒	☐ Antibodies
☒	☐ Eukaryotic cell lines
☒	☐ Palaeontology and archaeology
☒	☐ Animals and other organisms
☒	☐ Clinical data
☒	☐ Dual use research of concern
☒	☐ Plants

Methods

n/a	Involved in the study
☒	☐ ChIP-seq
☒	☐ Flow cytometry
☒	☐ MRI-based neuroimaging

Plants

Seed stocks	N/A
Novel plant genotypes	N/A
Authentication	N/A
